# Further Studies on the [1,2]-Wittig Rearrangement of 2-(2-Benzyloxy)aryloxazolines

**DOI:** 10.3390/molecules27103186

**Published:** 2022-05-17

**Authors:** R. Alan Aitken, Andrew D. Harper, Ryan A. Inwood

**Affiliations:** EaStCHEM School of Chemistry, University of St Andrews, North Haugh, St Andrews KY16 9ST, Fife, UK; andydharper@hotmail.com (A.D.H.); ri30@st-and.ac.uk (R.A.I.)

**Keywords:** oxazoline, Wittig rearrangement, phthalide

## Abstract

The behaviour of 14 ortho-functionalised 2-aryloxazolines (11 of them prepared and characterised for the first time) with butyllithium has been examined. Significant limitations to the Wittig rearrangement of such systems are revealed. In terms of asymmetric Wittig rearrangement, good diastereoselectivity is obtained with a valine-derived 4-isopropyl oxazoline, but this is compromised by racemisation upon hydrolysis. More encouraging selectivity is achieved in the Wittig rearrangement of an acyclic phenylalanine-derived *ortho*-benzyloxy benzamide.

## 1. Introduction

**Scheme 1:**
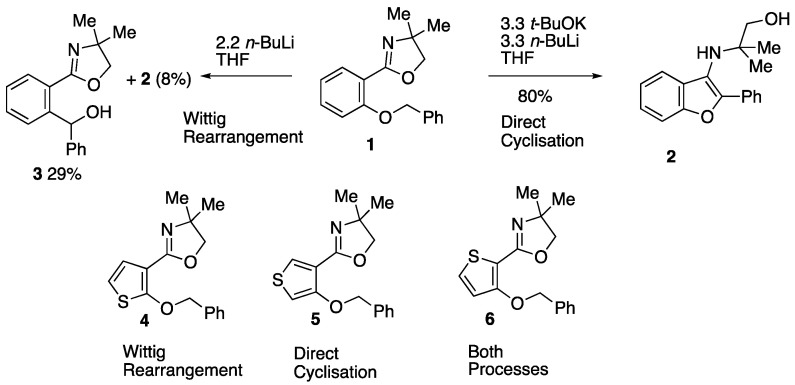
Competition between Wittig rearrangement and cyclisation in previously reported oxazoline systems [1,2].

**Scheme 2:**
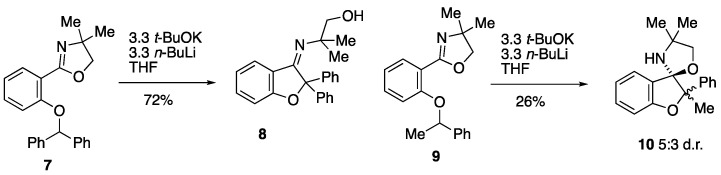
Previously reported cyclisation of α-branched 2-(2-benzyloxyphenyl)oxazolines [1].

**Scheme 3:**
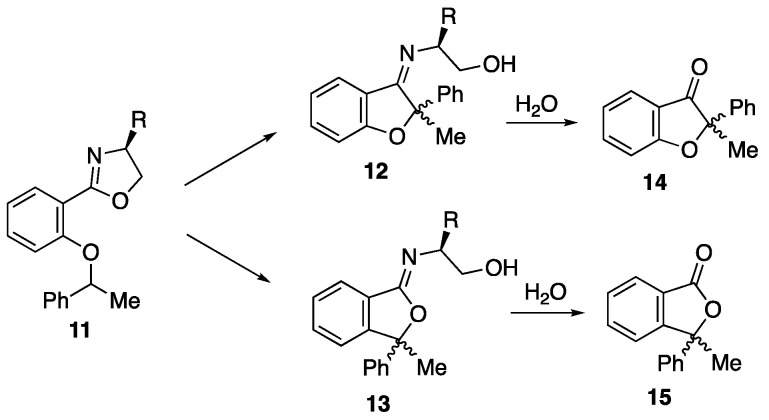
Potential products from cyclisation of chiral 2(2-(1-phenylethoxy)phenyl)oxazolines.

Some time ago we described the reaction of 2-(2-benzyloxyphenyl)oxazoline **1** with strong base to give either the 3-aminobenzofuran product **2** resulting from intramolecular nucleophilic ring-opening of the oxazoline by the benzyl anion, or the oxazoline **3** in which the benzyloxy group has undergone a Wittig rearrangement (Scheme 1) [1]. While the aminobenzofuran formation could be optimised by using 3.3 equiv. of Schlosser’s base (BuLi/KOBu^t^) and applied to a number of substituted examples [1], the Wittig rearrangement process was not so favourable and, under optimal conditions of 2.2 equiv. butyllithium (BuLi) in THF, an isolated yield of just 29% was obtained. More recently, we have studied the competition between Wittig rearrangement and direct anion cyclisation in a series of three isomeric (benzyloxythienyl)oxazolines **4**–**6** and found the outcome to depend upon the distance between the two groups in the starting compound [2]. In the meantime, we have found the *N*-butylamide group, CONHBu, to be far superior in promoting the Wittig rearrangement [3], but several aspects of the oxazoline chemistry remain unexplored. Specifically, although thia- and aza-analogues of the direct cyclisation of **1** giving benzothiophene and indole products were described [1], Wittig rearrangement of these substrates has not been examined until now. In addition, cyclisation of the α-branched benzhydryl ether **7** occurred to give the 3-iminodihydrobenzofuran **8** (Scheme 2) and for the α-methylbenzyl ether **9** cyclisation gave the spiro oxazolidine–dihydrobenzofuran **10** with some stereochemical control [1]. Given the illustrious history of chiral oxazolines in controlling a wide range of asymmetric processes [4,5,6], we were interested in exploring their ability to direct the reactions of an α-branched *ortho*-benzyloxy group. In general terms, we were interested to examine the stereoselective cyclisation of the anion derived from **11** to give either **12** from direct cyclisation or **13** from cyclisation following Wittig rearrangement (Scheme 3), with these products leading, respectively, to 2-substituted dihydrobenzofuran-3-ones **14** or 3-substituted dihydroisobenzofuranones (‘phthalides’) **15** following hydrolysis.

There have been few previous studies on diastereoselective Wittig rearrangement directed by either adjacent carbohydrate [7,8] or α-alkoxy amide [9] functions, and there is only one previous report of an enantioselective Wittig rearrangement mediated by a chiral bis(oxazoline) ligand [10].

In this paper we describe in detail the synthesis of a range of new chiral *ortho*-benzyloxyphenyl oxazolines and their base-induced cyclisation.

## 2. Results

### 2.1. Attempted Wittig Rearrangement of Benzylthio- and Benzylaminophenyloxazolines

**Scheme 4:**
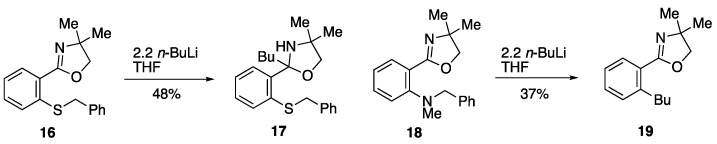
Outcome of attempted thia- and aza-Wittig rearrangements.

Treatment of the (benzylthiophenyl)oxazoline **16**, which reacted with Schlosser’s base to give the corresponding benzothiophene [1], with butyllithium in THF gave the oxazolidine **17** resulting from butyl addition at C-2 of the oxazoline (Scheme 4). This previously unknown compound was isolated in moderate yield, following preparative TLC. The corresponding reaction of the benzylmethylamino compound **18**, which with Schlosser’s base gave the indole, instead resulted in displacement of the amino group by butyl to give the previously known [11] oxazoline **19**.

### 2.2. Synthesis and Reactivity of 2-benzyloxy-3-pyridyloxazoline and N-butylamide

**Scheme 5:**
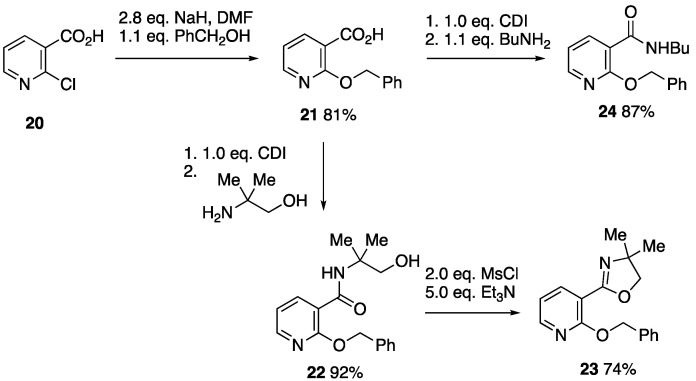
Synthesis of 2-benzyloxy-3-pyridyl compounds **23** and **24**.

2-Benzyloxypyridines readily undergo Wittig rearrangement on treatment with base [12,13,14], and so we expected that they would do so even more readily in the presence of an activating oxazoline or amide group at the 3-position. We therefore targeted compounds **23** and **24,** which were readily prepared starting from 2-chloronicotinic acid **20** (Scheme 5). Introduction of the 2-benzyloxy group by a S_N_Ar reaction to give **21** was followed by carbonyldiimidazole-mediated condensation with 2-amino-2-methylpropanol to afford **22**. Attempted cyclisation of this to oxazoline **23** using thionyl chloride resulted in loss of the *O*-benzyl group, but treatment with methanesulfonyl chloride in the presence of triethylamine yielded the required product. Similar condensation of **21** with butylamine gave the amide **24** in good yield. Unfortunately, treatment of both **23** and **24** with Schlosser’s base, butyllithium or LDA under a wide variety of conditions did not yield any useful products and it appears that, far from promoting the Wittig rearrangement as in benzene-based systems, the oxazoline and amide groups actually prevent it in the pyridine systems.

### 2.3. Synthesis and Reactivity of Chiral 2-(2-benzyloxyphenyl)oxazolines

**Scheme 6:**
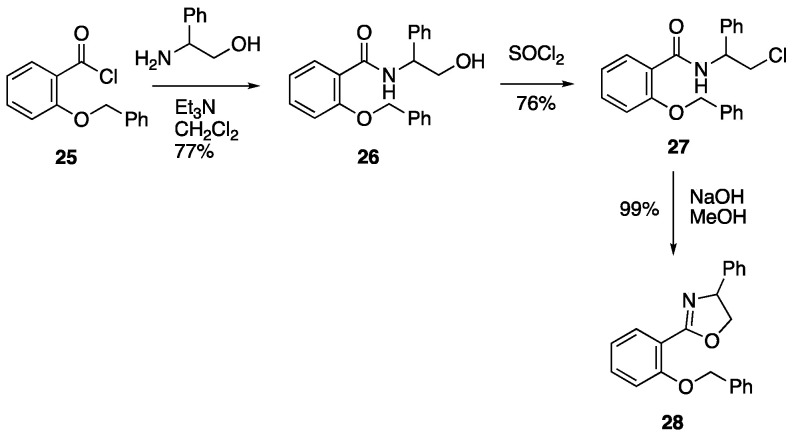
Synthesis of racemic oxazoline **28**.

Since all our previous studies have involved 4,4-dimethyloxazolines, we first prepared the racemic 4-phenyl compound **28,** starting from (±)-phenylglycinol and 2-benzyloxybenzoyl chloride **25** (Scheme 6). The resulting hydroxy amide **26** was expected to cyclise upon treatment with thionyl chloride, but instead the stable chloro amide **27** was formed. However, this could be cyclised to give the desired oxazoline **28** in essentially quantitative yield, using a literature method [15].

**Scheme 7:**
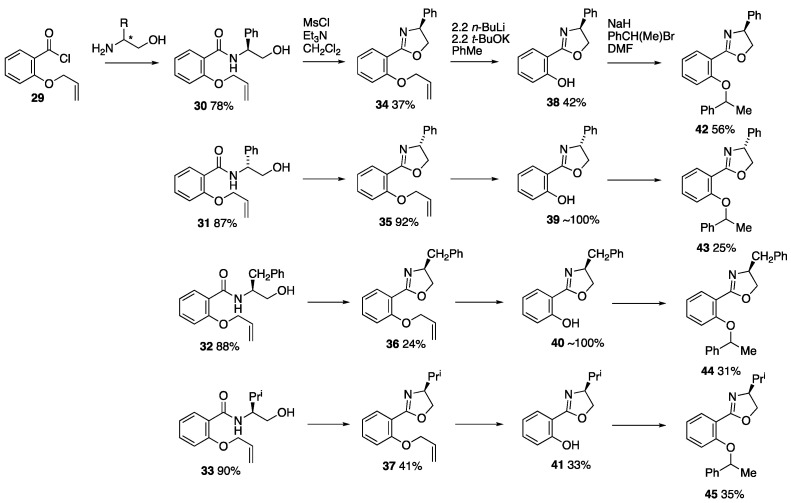
Synthesis of chiral oxazolines **42–45**.

For synthesis of the chiral oxazolines we made use of the fact that treatment of 2-(2-allyloxyphenyl)oxazolines with two equivalents of Schlosser’s base in toluene results in removal of the allyl group to give the corresponding phenol [1]. Thus, allyl could be used as a protecting group for the phenolic OH through the oxazoline synthesis (Scheme 7). Starting from 2-allyloxybenzoyl chloride **29**, and treating with (*S*)- and (*R*)-phenylglycinol, (*S*)-phenylalaninol and (*S*)-valinol, respectively, gave the hydroxy amides **30**–**33** in good yield, which were converted into oxazolines **34**–**37** using methanesulfonyl chloride and triethylamine. Deprotection to give hydroxyphenyl oxazolines **38**–**41** was then followed by *O*-alkylation, using α-methylbenzyl bromide to give the target oxazolines **42**–**45** as mixtures of diastereomers.

**Scheme 8:**
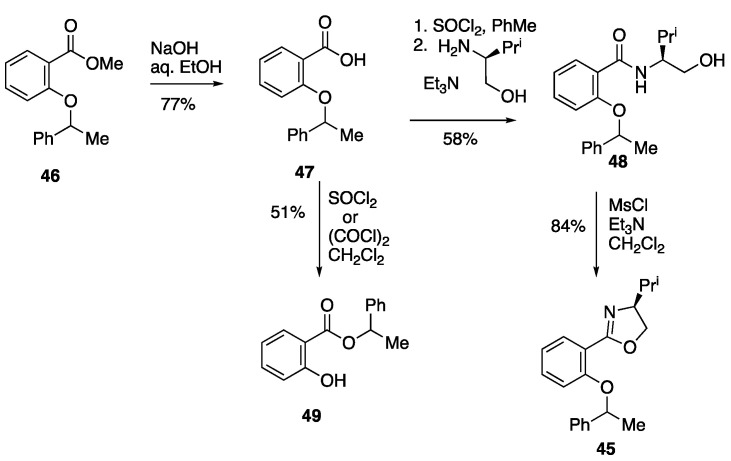
Alternative synthesis of oxazoline **45**.

Since some of the synthetic steps for the valinol-derived oxazoline **45**, which turned out to be the most synthetically useful, proceeded in low yield, an alternative synthesis involving installation of the α-methylbenzyl ether before oxazoline formation was examined and this did indeed give a higher overall yield (Scheme 8). Hydrolysis of the ester **46** formed by *O*-alkylation of methyl salicylate gave the acid **47** which was converted into its acid chloride and treated with valinol to give hydroxy amide **48** and this could be cyclised in the normal way to afford **45**. Attempted formation of the acid chloride from **47** in dichloromethane, as opposed to toluene, instead led to unexpected intramolecular transfer of the α-methylbenzyl group giving the known [16] salicylate **49**.

**Scheme 9:**
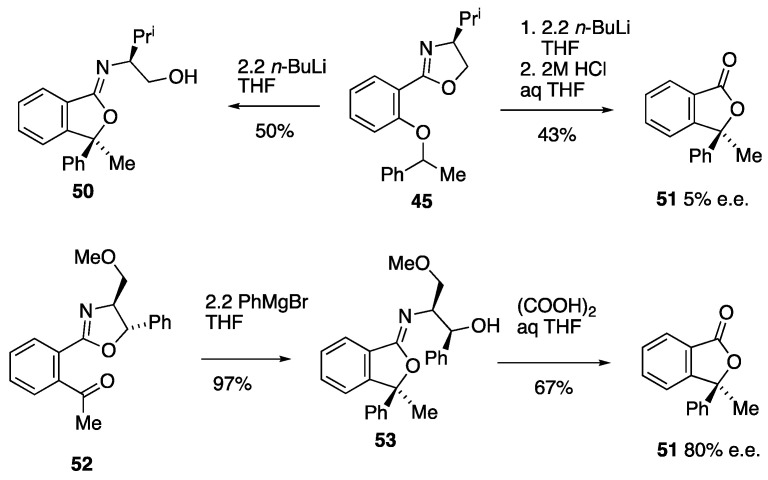
Products from Wittig rearrangement of **45** and an alternative approach to the same product [17].

When the four chiral oxazolines **42**–**45** were treated with 3.3 equivalents of Schlosser’s base, as used in the cyclisation of **9** to give **10**, there was complete decomposition and no useful products could be identified. It is clear that moving to the 4-monosubstituted oxazolines has opened the way to unwanted reaction pathways, and since deprotonation at a benzylic position may contribute to this in **42**–**44**, attention was focused on the valine-derived compound **45**, where this is not possible, to optimise the conditions. When oxazoline **45** was treated with 2.2 equivalents of butyllithium in the absence of potassium *tert*-butoxide, a cyclic product was formed which proved to have the iminophthalide structure **50** resulting from Wittig rearrangement followed by cyclisation (Scheme 9). Although this was isolated in only 50% yield after preparative TLC, it appeared to be a single stereoisomer so it was of interest to determine the stereochemistry at the newly formed centre by formation of **50,** followed by direct hydrolysis to the corresponding phthalide **51** for which the optical rotation is known. This proceeded in reasonable yield and showed that **51**, and thus also **50**, had the *S* configuration at the newly formed centre, but the rotation corresponded to only 5% e.e. It is interesting to compare the outcome here with the alternative oxazoline-based approach reported by Meyers [17], where reaction of the (2-acetylphenyl)oxazoline **52** with phenyl Grignard reagent gave the iminophthalide **53** which was then hydrolysed to afford **51** in 80% e.e. Application of the oxalic acid hydrolysis method in our case did not improve the e.e., and it seems that the iminophthalide **50** is much more susceptible to racemisation upon hydrolysis than **53**.

**Scheme 10:**
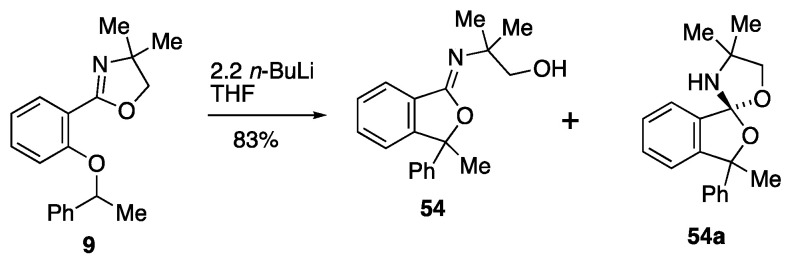
Formation of Wittig rearrangement products from oxazoline **9**.

It seemed likely that the change from direct cyclisation, as observed for **7** and **9,** to Wittig rearrangement followed by cyclisation in the case of **45** is a result of the change from Schlosser’s base to butyllithium, and this was confirmed by subjecting compound **9** [1] to the latter conditions which indeed produced the rearranged product **54** (Scheme 10). Interestingly, this product seems to exist in solution in equilibrium with a minor quantity of the spiro-oxazolidine form **54a,** for which separate signals are observed by NMR.

**Scheme 11:**
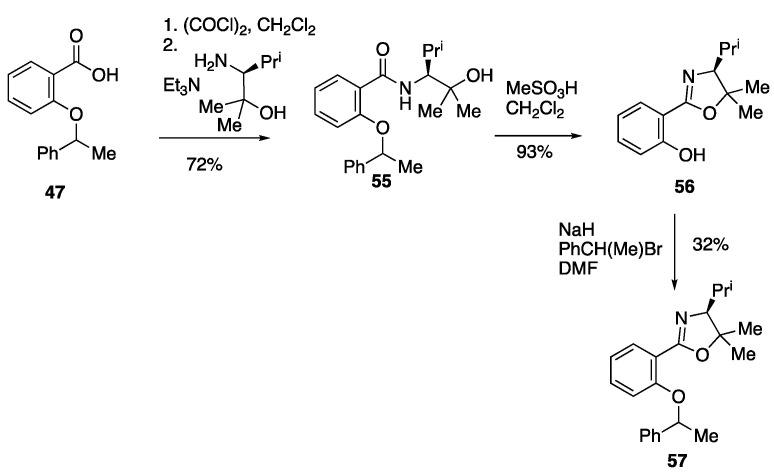
Synthesis of a 4-isopropyl-5,5-dimethyl oxazoline.

In an attempt to achieve higher selectivity, we examined the use of the 4-isopropyl-5,5-dimethyloxazoline group [18,19]. Reaction of the amino alcohol with the acid chloride derived from **47** led to the hydroxy amide **55** (Scheme 11). Unfortunately, the high yielding cyclisation of this to form the oxazoline was accompanied by loss of the α-methylbenzyl group to give **56,** which could be re-alkylated to afford the target oxazoline **57**.

**Scheme 12:**
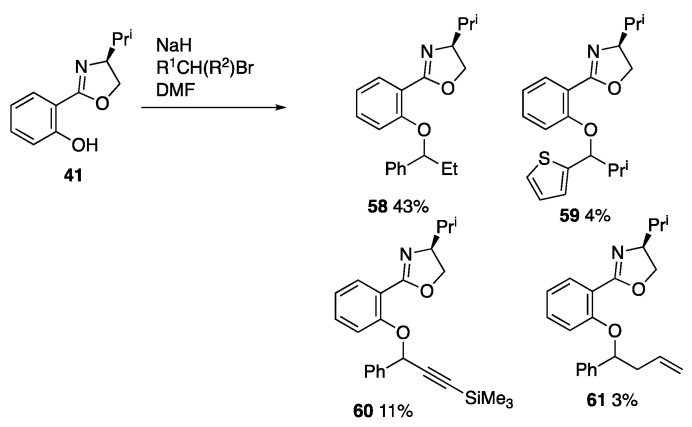
Variation of the secondary alkoxy substituent.

When this was treated with 2.2 equivalents of butyllithium there was extensive decomposition and no useful products could be separated. It therefore appeared that the simple valine-derived 4-isopropyloxazoline was the most promising auxiliary group and, with this in mind, the secondary alkoxy group was varied by alkylation of phenolic oxazoline **41** with the appropriate alkyl halides to give new chiral oxazolines **58–61** (Scheme 12). Although most of these were obtained in low yield they were fully characterised in each case.

Unfortunately, treatment of each of these compounds with 2.2 equivalents of butyllithium under the conditions optimised for **45** led only to decomposition and no useful products could be separated from the complex product mixtures.

**Scheme 13:**
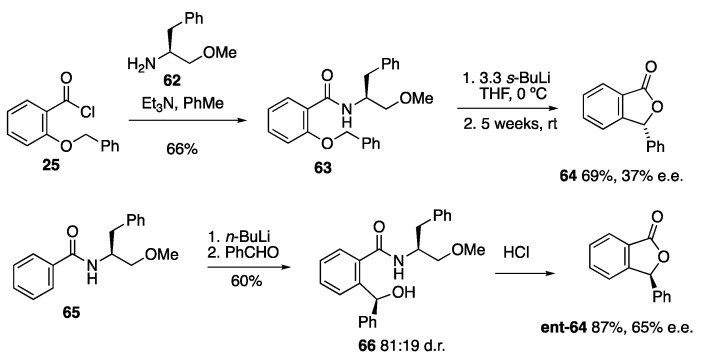
Wittig rearrangement of a chiral benzyloxy amide and an alternative approach to the same product [20].

In the light of the overall disappointing results obtained from Wittig rearrangement of the chiral oxazolines, and our discovery in the meantime [3] that a secondary benzamide could act as a much better promoter of the Wittig rearrangement, we decided to examine an asymmetric version of this process using the phenylalanine-derived alkoxy amine auxiliary **62** (Scheme 13). The amide **63** was readily prepared and treatment with 3.3. equivalents of an alkyllithium base did result in Wittig rearrangement. While use of *n*-butyllithium at either room temperature, 0 °C or −78 °C gave diastereomeric ratios of about 60:40, the best selectivity of 68:32 was obtained by using *s*-butyllithium at 0 °C. Furthermore, the resulting hydroxy amide underwent spontaneous cyclisation with regeneration of the chiral alkoxy amine auxiliary upon storage under normal laboratory conditions for a few weeks. Purification produced the phthalide **64** in good yield and with an e.e. of 37%.

It is interesting to compare this result with the work of Matsui and coworkers [20] who used the same secondary amide auxiliary group to direct *ortho*-lithiation of **65** and reaction with benzaldehyde to give **66**, the diastereomer of the Wittig rearrangement intermediate, which was then cyclised upon acid treatment to produce the enantiomer of **64** (Scheme 13). Given the much more encouraging result with amide **63,** future work will focus on secondary amides rather than oxazolines to direct the asymmetric Wittig rearrangement of an adjacent benzylic ether group.

## 3. Experimental

### 3.1. General Experimental Details

NMR spectra were recorded on solutions in CDCl_3_, unless otherwise stated, using Bruker instruments and chemical shifts are given in ppm to high frequency from Me_4_Si with coupling constants *J* in Hz. IR spectra were recorded using the ATR technique on a Shimadzu IRAffinity 1S instrument. The ionisation method used for high-resolution mass spectra is noted in each case. Column chromatography was carried out using silica gel of 40–63 μm particle size and preparative TLC was carried out using 1.0 mm layers of Merck alumina 60 G containing 0.5% Woelm fluorescent green indicator on glass plates. Melting points were recorded on a Gallenkamp 50 W melting point apparatus or a Reichert hot-stage microscope.

### 3.2. Attempted Thia- and Aza-Wittig Rearrangements

#### 3.2.1. Attempted [1,2]-Wittig Rearrangement of 2-(2-(Benzylthio)phenyl)-4,4-dimethyl-4,5-dihydrooxazole **16**

Under a nitrogen atmosphere, *n*-butyllithium (2.5 M in hexane, 0.44 cm^3^, 1.10 mmol) was added dropwise to a stirred −78 °C solution of 2-(2-(benzylthio)phenyl)-4,4-dimethyl-4,5-dihydrooxazole **16** (0.15 g, 0.50 mmol) in dry THF (5 cm^3^). After stirring at −78 °C for 2 h, the reaction mixture was quenched by addition of sat. aq. NH_4_Cl (20 cm^3^) and extracted with Et_2_O (3 × 20 cm^3^). The combined organic layers were dried and evaporated and the crude residue was purified by preparative TLC (SiO_2_, Et_2_O/hexane 4:1) to give, at R_f_ 0.95, 2-(2-(benzylthio)phenyl)-2-butyl-4,4-dimethyloxazolidine **17** (86.0 mg, 48%) as a yellow oil; ν_max_/cm^−1^ 1454, 1207, 1034, 972, 752 and 698; δ_H_ (500 MHz) 7.65 (1 H, d, *J* 8.0, ArH), 7.33 (1 H, dd, *J* 8.0, 1.0, ArH), 7.31–7.19 (6 H, m, ArH), 7.06 (1 H, ddd, *J* 7.8, 7.3, 1.3, ArH), 3.47 (1 H, half AB pattern, *J*_AB_ 14.0, SC***H***H), 3.38 (1 H, half AB pattern, *J*_AB_ 7.5, OC***H***H), 3.31 (1 H, half AB pattern, *J*_AB_ 14.0, SCH***H***), 3.27 (1 H, half AB pattern, *J*_AB_ 7.5, OCH***H***), 2.98 (2 H, t, *J* 7.5, C***H***_2_CH_2_CH_2_CH_3_), 2.08 (1 H, br s, NH), 1.75–1.69 (2 H, m, CH_2_C***H***_2_CH_2_CH_3_), 1.55–1.48 (2 H, m, CH_2_CH_2_C***H***_2_CH_3_), 0.95 (3 H, t, *J* 7.5, CH_2_CH_2_CH_2_C***H***_3_), 0.89 (3 H, s, oxazolidine CH_3_) and 0.81 (3 H, s, oxazolidine CH_3_); δ_C_ (125 MHz) 143.6 (C), 136.3 (C), 136.1 (C), 131.0 (2CH), 128.0 (2CH), 127.5 (CH), 127.3 (CH), 127.2 (CH), 126.6 (CH), 124.1 (CH), 99.4 (C), 76.8 (OCH_2_), 59.3 (C), 44.1 (SCH_2_), 33.2 (CH_2_), 30.9 (CH_2_), 27.7 (CH_3_), 27.2 (CH_3_), 22.3 (CH_2_) and 13.7 (CH_3_); HRMS (ESI^+^): found, M+H^+^, 356.2032. C_22_H_30_NOS^+^ requires *M*, 356.2043.

#### 3.2.2. Attempted [1,2]-Wittig Rearrangement of *N*-Benzyl-2-(4,4-dimethyl-4,5-dihydrooxazol-2-yl)-*N*-methylaniline **18**

Under a nitrogen atmosphere, *n*-butyllithium (2.5 M in hexane, 0.40 cm^3^, 1.00 mmol) was added dropwise to a stirred solution of *N*-benzyl-2-(4,4-dimethyl-4,5-dihydrooxazol-2-yl)-*N*-methylaniline **18** (0.15 g, 0.51 mmol) in dry THF (5 cm^3^). After stirring at rt for 2 h, the reaction mixture was quenched by addition of sat. aq. NH_4_Cl (20 cm^3^) and extracted with Et_2_O (3 × 25 cm^3^). The combined organic layers were dried and evaporated to give, after purification by preparative TLC (SiO_2_, Et_2_O/hexane 1:1), at R_f_ 0.85, 2-(2-butylphenyl)-4,4-dimethyl-4,5-dihydrooxazole **19** (43.9 mg, 37%) as an orange oil; δ_H_ (400 MHz) 7.68 (1 H, dd, *J* 7.6, 1.2, ArH), 7.32 (1 H, td, *J* 7.6, 1.2, ArH), 7.23–7.17 (2 H, m, ArH), 4.07 (2 H, s, oxazoline CH_2_), 2.94 (2 H, t, *J* 7.8, C***H***_2_CH_2_CH_2_CH_3_), 1.59–1.51 (2 H, m, CH_2_C***H***_2_CH_2_CH_3_), 1.40–1.31 (2 H, m, CH_2_CH_2_C***H***_2_CH_3_), 1.38 (6 H, s, oxazoline CH_3_) and 0.92 (3 H, t, *J* 7.4, CH_2_CH_2_CH_2_C***H***_3_); δ_C_ (100 MHz) 162.8 (C=N), 143.1 (C), 130.3 (CH), 130.2 (CH), 129.9 (CH), 127.5 (C), 125.5 (CH), 78.7 (OCH_2_), 67.7 (C), 33.80 (CH_2_), 33.78 (CH_2_), 28.4 (2CH_3_), 22.7 (CH_2_) and 13.9 (CH_3_). The ^1^H and ^13^C NMR spectral data (Supplementary Materials) were in accordance with those previously reported [11].

### 3.3. Synthesis of 2-Benzyloxy-3-pyridyl Systems

#### 3.3.1. 2-(Benzyloxy)nicotinic Acid **21**

Following a literature procedure [21], 2-chloronicotinic acid **20** (5.51 g, 35.0 mmol) was added in small portions to a stirred 0 °C suspension of sodium hydride (60% in mineral oil, 3.87 g, 96.8 mmol) in DMF (55 cm^3^). After stirring at 0 °C for 30 min, benzyl alcohol (4.19 g, 38.7 mmol) was added and the reaction mixture was heated at 75 °C for 18 h. After cooling to rt, the reaction mixture was poured into 1 M HCl (140 cm^3^) and extracted with Et_2_O (4 × 75 cm^3^). The combined organic extracts were washed with brine (5 × 100 cm^3^) before being dried and evaporated. The crude residue was recrystallised (EtOAc) to give **21** (6.52 g, 81%) as colourless crystals; mp 132–135 °C; (lit. [21] 134.5–136 °C); δ_H_ (500 MHz) 10.67 (1 H, br s, CO_2_H), 8.49 (1 H, dd, *J* 7.5, 2.0, ArH), 8.41 (1 H, dd, *J* 4.8, 2.0, ArH), 7.48 (2 H, d, *J* 7.0, Ph), 7.44–7.37 (3 H, m, Ph), 7.15 (1 H, dd, *J* 7.5, 4.8, ArH) and 5.64 (2 H, s, CH_2_). The ^1^H NMR spectral data were in accordance with those previously reported [21].

#### 3.3.2. 2-(Benzyloxy)-*N*-(1-hydroxy-2-methylpropan-2-yl)nicotinamide **22**

A literature procedure [21] was adapted as follows: 1,1′-Carbonyldiimidazole (2.94 g, 18.1 mmol) was added to a stirred suspension of 2-(benzyloxy)nicotinic acid **21** (4.16 g, 18.1 mmol) in CH_2_Cl_2_ (20 cm^3^). After stirring at rt for 15 min, the resultant solution was added to a solution of 2-amino-2-methylpropan-1-ol (1.79 g, 20.1 mmol) in CH_2_Cl_2_ (10 cm^3^) and the reaction mixture was stirred at rt for 18 h before being diluted with Et_2_O (75 cm^3^) and washed with 0.25 M HCl (2 × 50 cm^3^). The organic layer was dried and evaporated to give **22** (5.00 g, 92%) as a colourless oil which was used without further purification; ν_max_/cm^−1^ 3377, 1647, 1585, 1541, 1427, 1319, 1277, 984, 777 and 702; δ_H_ (500 MHz) 8.51 (1 H, dd, *J* 7.5, 2.0, ArH), 8.31 (1 H, dd, *J* 4.8, 2.0, ArH), 8.16 (1 H, br s, NH), 7.50–7.48 (2 H, m, Ph), 7.45–7.38 (3 H, m, Ph), 7.10 (1 H, dd, *J* 7.5, 4.8, ArH), 5.47 (2 H, s, OCH_2_Ph), 4.94 (1 H, t, *J* 5.5, OH), 3.56 (2 H, d, *J* 5.5, C*H*_2_OH) and 1.12 (6 H, s, CH_3_); δ_C_ (125 MHz) 164.2 (C), 160.0 (C), 149.7 (CH), 141.6 (CH), 135.7 (C), 128.9 (2CH), 128.80 (CH), 128.78 (2CH), 118.1 (CH), 116.0 (C), 70.9 (CH_2_), 69.4 (CH_2_), 56.3 (C) and 24.5 (2CH_3_); HRMS (NSI^+^): found 301.1545. C_17_H_21_N_2_O_3_ (M + H) requires 301.1547.

#### 3.3.3. 2-(2-(Benzyloxy)pyridin-3-yl)-4,4-dimethyl-4,5-dihydrooxazole **23**

Methanesulfonyl chloride (2.6 cm^3^, 3.85 g, 33.6 mmol) was added dropwise to a stirred 0 °C solution of 2-(benzyloxy)-*N*-(1-hydroxy-2-methylpropan-2-yl)nicotinamide **22** (5.00 g, 16.6 mmol) and triethylamine (11.6 cm^3^, 8.42 g, 83.2 mmol) in CH_2_Cl_2_ (40 cm^3^) and the reaction mixture was allowed to warm to rt for 18 h before being poured into water (50 cm^3^). The two layers were separated and the aqueous layer was re-extracted with CH_2_Cl_2_ (2 × 50 cm^3^). The combined organic layers were washed with 2 M HCl (50 cm^3^), 2 M NaOH (50 cm^3^) and water (50 cm^3^) before being dried and evaporated to give **23** (3.47 g, 74%) as a yellow oil which partially solidified on standing and was used without further purification; ν_max_/cm^−1^ 2963, 1647, 1585, 1435, 1360, 1321, 1292, 1036, 775, 735 and 698; δ_H_ (500 MHz) 8.25 (1 H, dd, *J* 5.0, 2.0, ArH), 8.07 (1 H, dd, *J* 7.5, 2.0, ArH), 7.54 (2 H, m, Ph), 7.37–7.34 (2 H, m, Ph), 7.29–7.26 (1 H, m, Ph), 6.94 (1 H, dd, *J* 7.5, 5.0, ArH), 5.53 (2 H, s, OCH_2_Ph), 4.12 (2 H, s, oxazoline CH_2_) and 1.41 (6 H, s, CH_3_); δ_C_ (125 MHz) 161.1 (C), 159.9 (C), 149.1 (CH), 140.1 (CH), 137.4 (C), 128.2 (2CH), 127.2 (CH), 126.8 (2CH), 116.5 (CH), 112.5 (C), 79.0 (OCH_2_), 67.6 (OCH_2_Ph), 46.0 (C) and 28.4 (2CH_3_); HRMS (NSI^+^): found 283.1440. C_17_H_19_N_2_O_2_ (M + H) requires 283.1441.

#### 3.3.4. 2-(Benzyloxy)-*N*-butylnicotinamide **24**

A literature procedure [21] was adapted as follows: 1,1′-Carbonyldiimidazole (2.48 g, 15.3 mmol) was added to a stirred suspension of 2-(benzyloxy)nicotinic acid **21** (3.50 g, 15.3 mmol) in CH_2_Cl_2_ (20 cm^3^). After stirring at rt for 15 min, the resultant solution was added to a solution of *n*-butylamine (1.7 cm^3^, 1.26 g, 17.2 mmol) in CH_2_Cl_2_ (5 cm^3^) and the reaction mixture was stirred at rt for 18 h before being diluted with Et_2_O (70 cm^3^) and washed with 0.25 M HCl (2 × 40 cm^3^). The organic layer was dried and evaporated to give **24** (3.77 g, 87%) as a colourless oil which was used without further purification; ν_max_/cm^−1^ 3406, 2930, 1647, 1585, 1524, 1425, 1306, 1231, 1155, 1098, 986, 750 and 698; δ_H_ (500 MHz) 8.55 (1 H, dd, *J* 7.5, 2.0, ArH), 8.28 (1 H, dd, *J* 4.5, 2.0, ArH), 7.93 (1 H, br s, NH), 7.48–7.46 (2 H, m, Ph), 7.44–7.35 (3 H, m, Ph), 7.09 (1 H, dd, *J* 7.5, 4.5, ArH), 5.50 (2 H, s, OCH_2_), 3.37 (2 H, td, *J* 7.0, 5.0, NCH_2_), 1.42–1.36 (2 H, m, NCH_2_C*H*_2_), 1.22–1.15 (2 H, m, C*H*_2_CH_3_) and 0.82 (3 H, t, *J* 7.5, CH_3_); δ_C_ (125 MHz) 163.6 (C), 160.2 (C), 149.2 (CH),141.6 (CH), 136.1 (C), 128.7 (2CH), 128.5 (CH), 128.4 (2CH), 118.0 (CH), 116.1 (C), 69.0 (OCH_2_), 39.4 (NCH_2_), 31.1 (CH_2_), 20.0 (CH_2_) and 13.7 (CH_3_); HRMS (NSI^+^): found 285.1596. C_17_H_21_N_2_O_2_ (M + H) requires 285.1598.

### 3.4. Synthesis of Chiral Oxazolines

#### 3.4.1. 2-(Benzyloxy)-*N*-(2-hydroxy-1-phenylethyl)benzamide **26**

A solution of 2-(benzyloxy)benzoyl chloride **25** (6.27 g, 25.4 mmol) in CH_2_Cl_2_ (40 cm^3^) was added dropwise to a stirred 0 °C solution of (±)-2-amino-2-phenylethan-1-ol [22] (3.77 g, 27.5 mmol) and triethylamine (3.9 cm^3^, 2.83 g, 28.0 mmol) in CH_2_Cl_2_ (40 cm^3^). Once the addition was complete, the reaction mixture was allowed to warm to rt for 20 h before being poured into water (100 cm^3^). The two layers were separated and the aqueous layer was re-extracted with CH_2_Cl_2_ (2 × 50 cm^3^). The combined organic layers were washed with 2 M HCl (100 cm^3^), 2 M NaOH (100 cm^3^) and water (100 cm^3^) before being dried and evaporated to give **26** (6.83 g, 77%) as a pale yellow solid which was used without further purification; mp 143–146 °C; ν_max_/cm^−1^ 3377, 3032, 2943, 1622, 1597, 1549, 1485, 1449, 1302, 1236, 1070, 989, 748 and 694; δ_H_ (400 MHz) 8.55 (1 H, d, *J* 6.8, NH), 8.23 (1 H, dd, *J* 7.8, 1.8, ArH), 7.48–7.38 (6 H, m, ArH), 7.21–7.14 (3 H, m, ArH), 7.12–7.07 (2 H, m, ArH), 7.01–6.98 (2 H, m, ArH), 5.24 (1 H, td, *J* 6.8, 4.4, NCH), 5.16 and 5.12 (2 H, AB pattern, *J*_AB_ 10.2, OCH_2_Ph), 3.76–3.67 (2 H, m, C***H***_2_OH) and 2.61 (1 H, br s, OH); δ_C_ (100 MHz) 165.7 (C=O), 156.9 (C–O), 138.9 (C), 135.2 (C), 133.0 (CH), 132.5 (CH), 129.0 (CH), 128.9 (2CH), 128.72 (2CH), 128.70 (2CH), 127.5 (CH), 126.6 (2CH), 121.6 (CH), 121.3 (C), 112.3 (CH), 71.5 (OCH_2_Ph), 67.4 (CH_2_OH) and 56.4 (NCH); HRMS (NSI^+^): found 348.1595. C_22_H_22_NO_3_ (M + H) requires 348.1594.

#### 3.4.2. 2-(Benzyloxy)-*N*-(2-chloro-1-phenylethyl)benzamide **27**

Thionyl chloride (1.7 cm^3^, 2.77 g, 23.3 mmol) was added to a solution of 2-(benzyloxy)-*N*-(2-hydroxy-1-phenylethyl)benzamide **26** (6.50 g, 18.7 mmol) in CH_2_Cl_2_ (100 cm^3^) and the reaction mixture was stirred at rt for 18 h. The reaction mixture was washed with 2 M NaOH (100 cm^3^) and water (100 cm^3^) before being dried and evaporated to give, after purification of the crude residue by column chromatography (SiO_2_, Et_2_O/hexane 3:2), at R_f_ 0.70, **27** (5.21 g, 76%) as a colourless solid; mp 90–93 °C; ν_max_/cm^−1^ 3368, 3059, 1645, 1595, 1522, 1479, 1285, 1217, 1157, 991, 752, 694 and 621; δ_H_ (500 MHz) 8.63 (1 H, d, *J* 8.0, NH), 8.25 (1 H, dd, *J* 8.0, 2.0, ArH), 7.51–7.40 (6 H, m, ArH), 7.35–7.27 (1 H, m, ArH), 7.23–7.18 (2 H, m, ArH), 7.13–7.10 (2 H, m, ArH), 7.03–7.00 (2 H, m, ArH), 5.51 (1 H, dt, *J* 8.0, 5.5, NCH), 5.21 and 5.17 (2 H, AB pattern, *J*_AB_ 10.0, OCH_2_Ph), 3.74 (1 H, half AB pattern of d, *J*_AB_ 11.0, *J*_AX_ 5.0, C***H***HCl), 3.62 (1 H, half AB pattern of d, *J*_AB_ 11.0, *J*_AX_ 5.5, CH***H***Cl); δ_C_ (125 MHz) 164.5 (C=O), 157.0 (C–O), 138.7 (C), 135.2 (C), 133.1 (CH), 132.6 (CH), 129.1 (CH), 129.0 (2CH), 128.9 (2CH), 128.6 (2CH), 127.7 (CH), 126.7 (2CH), 121.6 (CH), 121.0 (C), 112.3 (CH), 71.5 (OCH_2_), 54.1 (NCH) and 48.0 (CH_2_Cl); HRMS (NSI^+^): found 366.1250. C_22_H_21_^35^ClNO_2_ (M + H) requires 366.1255.

#### 3.4.3. 2-(2-(Benzyloxy)phenyl)-4-phenyl-4,5-dihydrooxazole **28**

A literature procedure [15] was adapted as follows: A mixture of 2-(benzyloxy)-*N*-(2-chloro-1-phenylethyl)benzamide **27** (4.74 g, 13.0 mmol) and sodium hydroxide (0.82 g, 20.5 mmol) in methanol (100 cm^3^) was heated at reflux for 3 h. After cooling to rt, the reaction mixture was diluted with Et_2_O (250 cm^3^) and washed with brine (3 × 100 cm^3^). The organic layer was dried and evaporated to give **28** (4.24 g, 99%) as a colourless oil which solidified on standing; mp 47–51 °C; ν_max_/cm^−1^ 3061, 3034, 1665, 1493, 1447, 1250, 1034, 1001, 750 and 696; δ_H_ (500 MHz) 7.84 (1 H, dd, *J* 7.8, 1.8, ArH), 7.49 (2 H, d, *J* 7.0, Ph), 7.44–7.40 (1 H, m, ArH), 7.35–7.27 (8 H, m, ArH), 7.05–7.00 (2 H, m, ArH), 5.42 (1 H, dd, *J* 10.0, 8.0, NCH), 5.22 and 5.20 (2 H, AB pattern, *J*_AB_ 12.0, OCH_2_Ph), 4.78 (1 H, dd, *J* 10.0, 8.0, oxazoline C***H***H) and 4.26 (1 H, t, *J* 8.0, oxazoline CH***H***); δ_C_ (125 MHz) 163.8 (C=N), 157.5 (C–O), 142.6 (C), 136.8 (C), 132.3 (CH), 131.4 (CH), 128.6 (2CH), 128.4 (2CH), 127.6 (CH), 127.4 (CH), 127.0 (2CH), 126.8 (2CH), 120.7 (CH), 118.0 (C), 113.5 (CH), 74.4 (CH_2_), 70.6 (CH_2_) and 70.2 (NCH); HRMS (NSI^+^): found 330.1488. C_22_H_20_NO_2_ (M + H) requires 330.1489.

#### 3.4.4. 2-(Allyloxy)benzoyl Chloride **29**

Thionyl chloride (6.51 cm^3^, 10.68 g, 89.8 mmol) was added dropwise to a stirred solution of 2-(allyloxy)benzoic acid [23] **2** (8.00 g, 44.9 mmol) in toluene (70 cm^3^) at rt and the mixture heated to reflux for 3 h then cooled to rt and concentrated. The crude residue was purified via Kugelrohr distillation to give **29** (5.79 g, 66%) as a colourless oil, bp 182 °C/20 Torr (lit. [24] bp 80−82 °C/0.2 Torr); δ_H_ (400 MHz) 8.12 (1H, dd, *J* 8.1, 1.8, ArH), 7.58 (1H, ddd, *J* 8.4, 7.4, 1.8, ArH), 7.08 (1H, ddd, *J* 8.1, 7.4 1.0, ArH), 7.01 (1H, dd, *J* 8.4, 1.0, ArH), 6.07 (1H, ddt, *J* 17.2, 10.6, 4.8, C***H***=CH_2_), 5.56 (1H, dq, *J* 17.3, 1.7, CH=C***H***H), 5.36 (1H, dq, *J* 10.6, 1.5, CH=CH***H***) and 4.68 (2H, dt, *J* 4.8, 1.7, OCH_2_); δ_C_ (100 MHz) 163.7 (C=O), 158.5 (C-O), 136.0 (CH), 134.5 (CH), 131.8 (CH), 122.4 (C) 120.4 (CH), 117.8 (=CH_2_), 113.2 (CH) and 69.3 (OCH_2_). The ^1^H spectral data were in accordance with those previously reported [25]. The ^13^C spectral are reported for the first time.

#### 3.4.5. (*S*)-2-(Allyloxy)-*N*-(2-hydroxy-1-phenylethyl)benzamide **30**

A solution of 2-(allyloxy)benzoyl chloride **29** (5.00 g, 25.4 mmol) in CH_2_Cl_2_ (15 cm^3^) was stirred at 0 °C while triethylamine (3.53 cm^3^, 2.57 g, 25.4 mmol) was added dropwise, followed by dropwise addition of a solution of (*S*)-2-amino-2-phenylethan-1-ol [26] (3.48 g, 25.4 mmol) in CH_2_Cl_2_ (15 cm^3^). After stirring at RT for 18 h, the mixture was poured into water (50 cm^3^) and the organic layer was separated. Extraction of the aqueous layer with CH_2_Cl_2_ (2 × 20 cm^3^) followed by drying and evaporation of the combined organic solutions gave, after recrystallisation from EtOAc/hexane, **30** (5.86 g, 78%), colourless crystals; mp 65−67 °C; ν_max_/cm^−1^ 3300, 2947, 1632, 1601, 1539, 1233, 993, 934, 752, 719, 694 and 529; δ_H_ (400 MHz) 8.72 (1H, d, *J* 6.7 NH), 8.18 (1H, dd, *J* 7.8, 1.9, ArH), 7.44 (1H, ddd, *J* 8.3, 7.4, 1.9 ArH), 7.39−7.33 (4H, m, ArH), 7.33−7.28 (1H, m, ArH), 7.05 (1H, ddd, *J* 8.1, 7.4, 1.0, ArH), 6.93 (1H, dd, *J* 8.4, 1.0, ArH), 5.98 (1H, ddt, *J* 17.2, 10.4, 5.9, C***H***=CH_2_), 5.38 (1H, dq, *J* 17.2, 1.4, CH=C***H***H), 5.34−5.26 (2H, m, CH=CH***H*** and CHN), 4.63−4.57 (2H, m, OCH_2_), 3.92−3.88 (2H, m, CHC***H***_2_OH) and 3.49 (1H, s, OH); δ_C_ (100 MHz) 165.8 (C=O), 156.6 (C-O), 139.2 (C), 132.9 (***C***H=CH_2_), 132.2 (CH), 131.8 (CH), 128.7 (2CH), 127.6 (CH), 126.8 (2CH), 121.4 (CH), 121.1 (C), 119.7 (=CH_2_), 112.4 (CH), 70.0 (OCH_2_), 67.2 (CH_2_OH) and 56.7 (***C***HNH); [α]_D_ −32.4 (*c* 1.002, CH_2_Cl_2_); HRMS (NSI^+^): found 298.1438. C_18_H_20_NO_3_ (M + H) requires 298.1443.

#### 3.4.6. (*R*)-2-(Allyloxy)-*N*-(2-hydroxy-1-phenylethyl)benzamide **31**

Following the procedure of 3.4.5 using 2-(allyloxy)benzoyl chloride **29** (5.00 g, 25.4 mmol) in CH_2_Cl_2_ (15 cm^3^) and (*R*)-2-amino-2-phenylethan-1-ol [27] (3.48 g, 25.4 mmol) in CH_2_Cl_2_ (15 cm^3^) gave **31** (6.59 g, 87%) as colourless crystals; mp 71−73 °C; ν_max_/cm^−1^ 3348, 1624, 1522, 1234, 1034, 1026, 989, 920, 754, 700, 530 and 521; δ_H_ (400 MHz) 8.72 (1H, d, *J* 6.6, NH), 8.20 (1H, dd, *J* 7.8, 1.9, ArH), 7.44 (1H, ddd, *J* 8.4, 7.3, 1.9, ArH), 7.39−7.34 (4H, m), 7.33−7.28 (1H, m, ArH), 7.07 (1H, ddd, *J* 8.1, 7.3, 1.0 ArH), 6.95 (1H, dd, *J* 8.4, 1.0, ArH), 5.99 (1H, ddt, *J* 17.2, 10.4, 5.9, C***H***=CH_2_), 5.39 (1H, dq, *J* 17.2, 1.4, CH=C***H***H), 5.34−5.27 (2H, m, CH=CH***H*** and CHN), 4.70−4.56 (2H, m, OCH_2_), 4.02−3.91 (2H, m, CHC***H***_2_OH) and 3.33 (1H, s, OH); δ_C_ (100 MHz) 165.9 (C=O), 156.7 (C-O), 139.2 (C), 133.0 (***C***H=CH_2_), 132.3 (CH), 131.8 (CH), 128.8 (2CH), 127.7 (CH), 126.8 (2CH), 121.5 (CH), 121.2 (C), 119.8 (=CH_2_), 112.4 (CH), 70.1 (OCH_2_), 67.5 (CH_2_OH) and 56.9 (***C***HNH); [α]_D_ +30.9 (*c* 1.00, CH_2_Cl_2_); HRMS (NSI^+^): found 298.1439. C_18_H_20_NO_3_ (M + H) requires 298.1443.

#### 3.4.7. (*S*)-2-Allyloxy-*N*-(1-hydroxy-3-phenylpropan-2-yl)benzamide **32**

Following the procedure of 3.4.5 using 2-(allyloxy)benzoyl chloride **29** (5.06 g, 25.7 mmol) in CH_2_Cl_2_ (15 cm^3^) and (*S*)-2-amino-3-phenylpropan-1-ol [26] (3.88 g, 25.7 mmol) in CH_2_Cl_2_ (15 cm^3^) gave **32** (7.05 g, 88%) as colourless crystals; mp 98−101 °C; ν_max_/cm^−1^ 3391, 3354, 1616, 1541, 1314, 1229, 1045, 760, 752, 706 and 500; δ_H_ (400 MHz) 8.24 (1H, d, *J* 7.1, NH), 8.19 (1H, dd, *J* 7.8, 1.9, ArH), 7.42 (1H, ddd, *J* 8.3, 7.3, 1.9, ArH), 7.30−7.19 (5H, m), 7.07 (1H, ddd, *J* 7.8, 7.3, 1.0, ArH), 6.93 (1H, dd, *J* 8.4, 1.0 ArH), 5.95 (1H, ddt, *J* 17.3, 10.5, 5.6, C***H***=CH_2_), 5.38 (1H, dq, *J* 17.3, 1.5, CH=C***H***H), 5.33 (1H, dq, *J* 10.5, 1.2. CH=H***H***), 4.59 (2H, dt, *J* 5.6, 1.4, OCH_2_), 4.42 (1H, qdd, *J* 7.1, 5.5, 3.4, C***H***CH_2_Ph), 3.80 (1H, dd, *J* 11.1, 3.5, CH***H***OH), 3.69 (1H, dd, *J* 11.1, 5.6, C***H***HOH) and 2.96 (2H, dd, *J* 7.2, 1.6, CH_2_Ph); δ_C_ (100 MHz) 166.0 (C=O), 156.6 (C-O), 137.8 (C), 132.8 (***C***H=CH_2_), 132.2 (CH), 132.0 (CH), 129.2 (2CH), 128.5 (2CH), 126.5 (CH), 121.5 (CH), 121.4 (C), 119.2 (=CH_2_), 112.7 (CH), 69.9 (OCH_2_), 64.9 (CH_2_OH), 53.6 (CHN) and 37.1 (***C***H_2_Ph); [α]_D_ −69.4 (*c* 1.00, CH_2_Cl_2_); HRMS (NSI^+^): found 312.1596. C_19_H_22_NO_3_ (M + H) requires 312.1600.

#### 3.4.8. (*S*)-2-(Allyloxy)-*N*-(1-hydroxy-3-methylbutan-2-yl)benzamide **33**

Following the procedure of 3.4.5 using 2-(allyloxy)benzoyl chloride **29** (5.00 g, 25.4 mmol) in CH_2_Cl_2_ (15 cm^3^) and (*S*)-2-amino-3-methylbutan-1-ol [28] (2.61 g, 25.4 mmol) in CH_2_Cl_2_ (15 cm^3^) gave **33** (6.05 g, 90%) as colourless crystals; mp 94−96 °C; ν_max_/cm^−1^ 3343, 2954, 2870, 1612, 1599, 1541, 1233, 1067, 1013, 986, 920, 760, 606 and 598; δ_H_ (400 MHz) 8.24−8.18 (2H, m, NH and ArH), 7.44 (1H, ddd, *J* 8.4, 7.3, 1.9, ArH), 7.10 (1H, ddd, *J* 7.9, 1.3, 1.0, ArH), 6.98 (1H, dd, *J* 8.4, 1.0, ArH), 6.11 (1H, ddt, *J* 17.2, 10.3, 5.9, C***H***=CH_2_), 5.47 (1H, dq, *J* 17.2, 1.4, CH=C***H***H), 5.40 (1H, dq, *J* 10.3, 1.1, CH=CH***H***), 4.68 (2H, dt, *J* 5.9, 1.3, OCH_2_), 4.01 (1H, tdd, *J* 7.2, 6.0, 3.3, C***H***CH_2_OH), 3.81 (1H, dd, *J* 11.1, 3.3, CH***H***OH), 3.72 (1H, dd, *J* 11.1, 6.8, C***H***HOH), 2.00 (1H, m, C*H*Me_2_), 1.02 (3H, d, *J* 6.8, Me) and 0.99 (3H, d, *J* 6.8, Me); δ_C_ (100 MHz) 166.6 (C=O), 156.6 (C-O), 132.9 (***C***H=CH_2_), 132.4 (CH), 131.9 (CH), 121.5 (CH), 121.4 (C), 120.1 (CH=***C***H_2_), 112.4 (CH), 70.1 (OCH_2_), 65.2 (CH_2_OH), 58.1 (CHN), 29.2 (CH), 19.7 (CH_3_) and 18.5 (CH_3_); [α]_D_ −24.2 (*c* 1.002, CH_2_Cl_2_); HRMS (NSI^+^): found 264.1596. C_15_H_22_NO_3_ (M + H) requires 264.1600.

#### 3.4.9. (*S*)-2-(2-(Allyloxy)phenyl)-4-phenyl-4,5-dihydrooxazole **34**

A solution of (*S*)-2-(allyloxy)-*N*-(2-hydroxy-1-phenylethyl)benzamide **30** (1.0 g, 3.4 mmol) in CH_2_Cl_2_ (20 cm^3^) was stirred at 0 °C while MsCl (0.31 cm^3^, 0.46 g, 4.0 mmol) and then Et_3_N (1.03 cm^3^, 0.75 g, 7.4 mmol) were added dropwise. The mixture was then heated under reflux for 3 d. It was then cooled and added to water (20 cm^3^). Separation of the organic layer, extraction of the aqueous layer with CH_2_Cl_2_ (2 × 10 cm^3^), drying and evaporation of the combined organic extracts gave, after purification via flash column chromatography (hexane/EtOAc 7:3) at R_f_ 0.29, compound **34** (350 mg, 37%) as a pale yellow oil; ν_max_/cm^−1^ 3381, 3063, 2928, 1728, 1638, 1599, 1449, 1229, 1074, 993, 752, 698 and 525; δ_H_ (400 MHz) 7.83 (1H, dd, *J* 7.7, 1.8, ArH), 7.41 (1H ddd, *J* 8.4, 7.4, 1.8, ArH), 7.38−7.32 (4H, m, ArH), 7.31−7.26 (1H, m, ArH), 7.04−6.96 (2H, m, ArH), 6.07 (1H, ddt, *J* 17.3, 10.6, 4.8 C***H***=CH_2_), 5.51 (1H, dq, *J* 17.2, 1.8, CH=C***H***H), 5.41 (1H, dd, *J* 10.2, 8.0, C***H***HOCN), 5.26 (1H, dq, *J* 10.6, 1.6, CH=CH***H***), 4.77 (1H, dd, *J* 10.2, 8.3, CH***H***OCN), 4.65 (2H, dt, *J* 4.9, 1.7, ArOCH_2_) and 4.25 (1H, t, *J* 8.2, C***H***Ph); δ_C_ (100 MHz) 163.8 (C=N), 157.3 (C-O), 142.5 (C), 132.7 (***C***H=CH_2_), 132.1 (CH), 131.2 (CH), 128.4 (2CH), 127.3 (CH), 126.6 (2CH), 120.4 (CH), 117.6 (C), 117.1 (=CH_2_), 113.2 (CH), 74.3 (OCH_2_), 69.9 (CHN) and 69.3 (OCH_2_); [α]_D_ −24.5 (*c* 1.00, CH_2_Cl_2_); HRMS (NSI^+^): found 280.1335. C_18_H_18_NO_2_ (M + H) requires 280.1338.

#### 3.4.10. (*R*)-2-(2-(Allyloxy)phenyl)-4-phenyl-4,5-dihydrooxazole **35**

Following the procedure of 3.4.9 using (*R*)-2-(allyloxy)-*N*-(2-hydroxy-1-phenylethyl)benzamide **31** (6.9 g, 22.2 mmol), MsCl (2.06 cm^3^, 3.05 g, 26.6 mmol) and Et_3_N (6.80 cm^3^, 4.93 g, 48.8 mmol) in CH_2_Cl_2_ (50 cm^3^) at 50 °C overnight gave, after purification via flash column chromatography (hexane/EtOAc 7:3) at R_f_ 0.29, compound **35** (5.67 g, 92%) as a pale yellow oil; ν_max_/cm^−1^ 3381, 3030, 2932, 2874, 1638, 1599, 1450, 1229, 993, 752, 698 and 525; δ_H_ (400 MHz) 7.82 (1H, dd, *J* 7.7, 1.8, ArH), 7.41 (1H, ddd, *J* 8.4, 7.4, 1.8, ArH), 7.37–7.33 (4H, m, ArH), 7.30−7.26 (1H, m, ArH), 7.02−6.95 (2H, m, ArH), 6.06 (1H, ddt, *J* 17.3, 10.6, 4.8, C***H***=CH_2_), 5.50 (1H, dq, *J* 17.3, 1.7, CH=C***H***H), 5.40 (1H, dd, *J* 10.2, 7.9, C***H***HOCN), 5.26 (1H, dq, *J* 10.6, 1.6, CH=CH***H***), 4.76 (1H, dd, *J* 10.2, 8.3, CH***H***OCN), 4.64 (2H, dt, *J* 4.8, 1.7, ArOCH_2_) and 4.24 (1H, t, *J* 8.1, CHPh); δ_C_ (125 MHz) 163.9 (C=N), 157.4 (C-O), 142.6 (C), 132.8 (***C***H=CH_2_), 132.2 (CH), 131.3 (CH), 128.5 (2CH), 127.4 (CH), 126.7 (2CH), 120.5 (CH), 117.7 (C), 117.2 (=CH_2_), 113.3 (CH), 74.5 (OCH_2_), 70.0 (CHN) and 69.4 (OCH_2_); [α]_D_ +22.7 (*c* 0.93, CH_2_Cl_2_); HRMS (NSI^+^): found 280.1334. C_18_H_18_NO_2_ (M + H) requires 280.1338.

#### 3.4.11. (*S*)-2-(2-(Allyloxy)phenyl)-4-benzyl-4,5-dihydrooxazole **36**

Following the procedure of 3.4.9 using (*S*)-*N*-(1-hydroxy-3-phenylpropan-2-yl)-2-propoxybenzamide **32** (1.0 g, 3.2 mmol), MsCl (0.30 cm^3^, 0.44 g, 3.9 mmol) and Et_3_N (0.98 cm^3^, 0.71 g, 7.1 mmol) in CH_2_Cl_2_ (20 cm^3^) at rt overnight gave, after purification via flash column chromatography (hexane/EtOAc 7:3) at R_f_ 0.34, **36** (220 mg, 24%) as a pale-yellow oil; ν_max_/cm^−1^ 3393, 3026, 2893, 1643, 1601, 1493, 1450, 1256, 1227, 995, 750 and 698; δ_H_ (400 MHz) 7.74 (1H, dd, *J* 7.7, 1.8, ArH), 7.39 (1H, ddd, *J* 8.3, 7.4, 1.8, ArH), 7.34−7.27 (4H, m, ArH), 7.25−7.20 (1H, m, ArH), 7.01−6.93 (2H, m, ArH), 6.06 (1H, ddt, *J* 17.2, 10.6, 4.7, C***H***=CH_2_), 5.53 (1H, dq, *J* 17.2, 1.7, CH=C***H***H), 5.28 (1H, dq, *J* 10.6, 1.7, CH=CH***H***), 4.64 (2H, dt, *J* 4.7, 1.4, ArOCH_2_), 4.62−4.56 (1H, m, C***H***CH_2_Ph), 4.33 (1H, dd, *J* 9.4, 8.4, C***H***HO), 4.11 (1H, dd, *J* 8.5, 7.4, CH***H***O), 3.25 (1H, dd, *J* 13.7, 5.2, C***H***HPh) and 2.77 (1H, dd, *J* 13.7, 8.7, CH***H***Ph); δ_C_ (100 MHz) 162.7 (C=N), 157.2 (C-O), 137.9 (C), 132.7 (CH), 131.9 (CH), 131.1 (CH), 129.1 (2CH), 128.3 (2CH), 126.2 (CH), 120.3 (CH), 117.6 (C), 116.9 (C=***C***H_2_), 113.2 (CH), 71.1 (OCH_2_), 69.2 (OCH_2_), 67.8 (CHN) and 41.6 (CH_2_); [α]_D_ −30.89 (*c* 1.01, CH_2_Cl_2_); HRMS (NSI^+^): found 294.1491. C_19_H_20_NO_2_ (M + H) requires 294.1494.

#### 3.4.12. (*S*)-2-(-2(Allyloxy)phenyl)-4-isopropyl-4,5-dihydrooxazole **37**

Following the procedure of 3.4.9 using (*S*)-2-(allyloxy)-*N*-(1-hydroxy-3-methylbutan-2-yl)benzamide **33** (1.0 g, 3.8 mmol), MsCl (0.38 cm^3^, 0.56 g, 4.6 mmol) and Et_3_N (1.17 cm^3^, 0.85 g, 8.4 mmol) in CH_2_Cl_2_ (20 cm^3^) at rt overnight gave, after purification via flash column chromatography (hexane/EtOAc 7:3) at R_f_ 0.35, **37** (380 mg, 41%) as a pale-yellow oil; ν_max_/cm^−1^ 3399, 2961, 2874, 1645, 1599, 1449, 1292, 1227, 1037, 995, 924, 752, 552 and 527; δ_H_ (400 MHz) 7.72 (1H, dd, *J* 7.6, 1.8 ArH), 7.37 (1H, ddd, *J* 8.3, 7.4, 1.8, ArH), 7.02−6.91 (2H, m, ArH), 6.05 (1H, ddt, *J* 17.2, 10.6, 4.7, C***H***=CH_2_), 5.53 (1H, dq, *J* 17.2, 1.8, CH=***H***H), 5.27 (1H, dq, *J* 10.6, 1.6, CH=CH***H***), 4.61 (2H, dt, *J* 4.7, 1.7, ArOCH_2_), 4.45−4.35 (1H, m, C***H***CH(CH_3_)_2_), 4.14−4.07 (2H, m, CH_2_OCN), 1.95−1.84 (1H, m, C***H***(CH_3_)_2_), 1.04 (3H, d, *J* 6.8, CH_3_) and 0.95 (3H, d, *J* 6.7, CH_3_); δ_C_ (100 MHz) 162.0 (C=N), 156.9 (C-O), 132.5 (***C***H=CH_2_), 131.5 (CH), 130.8 (CH), 120.1 (CH), 117.8 (C), 116.6 (C=***C***H_2_), 112.8 (Ar CH), 72.3 (CHN), 69.3 (OCH_2_), 68.9 (OCH_2_), 32.4 (CH), 18.4 (CH_3_) and 17.8 (CH_3_); [α]_D_ −43.44 (*c* 1.006, CH_2_Cl_2_); HRMS (NSI^+^): found 246.1486. C_15_H_21_NO_2_ (M + H) requires 246.1494.

#### 3.4.13. (*S*)-2-(4-Phenyl-4,5-dihydrooxazol-2-yl)phenol **38**

A solution of (*S*)-2-(2-(allyloxy)phenyl)-4-phenyl-4,5-dihydrooxazole **34** (4.58 g, 16.4 mmol) and KOBu^t^ (4.05 g, 36.1 mmol) in PhMe (100 cm^3^) was stirred at rt under a nitrogen atmosphere while *n*-butyllithium (14.4 cm^3^, 36.1 mmol) was added. After 2 h, the solution was added to aqueous ammonium chloride solution (50 cm^3^) and the mixture was extracted with Et_2_O (3 × 50 cm^3^). Drying and evaporation of the combined organic extracts gave crude **38** (3.80 g, 97%) as a dark-red oil, which was purified via flash column chromatography (hexane/Et_2_O 1:1) to give, at R_f_ 0.67, **38** (1.65 g, 42%) as a yellow oil; δ_H_ (400 MHz) 7.72 (1H, dd, *J* 7.8, 1.7, ArH), 7.43−7.40 (1H, m, ArH), 7.38−7.36 (2H, m, ArH), 7.34−7.28 (3H, m, ArH), 7.04 (1H, dd, *J* 8.4, 1.2, 0.4 ArH), 6.91 (1H, ddd, *J* 7.8, 7.3, 1.2, ArH), 5.47 (1H, dd, *J* 10.1, 8.3, oxazoline NCH), 4.82 (1H, dd, *J* 10.1, 8.3, oxazoline OC***H***H) and 4.27 (1H, t, *J* 8.3, oxazoline OCH***H***); δ_C_ (100 MHz) 166.4 (C=N), 160.2 (C-O), 141.7 (C), 133.8 (CH), 129.0 (2CH), 128.4 (CH), 128.0 (CH), 126.6 (CH), 118.9 (CH), 117.0 (CH), 110.6 (C), 74.3 (CH_2_) and 68.7 (CH); HRMS (NSI^+^): found 240.1020. C_15_H_14_NO_2_ (M + H) requires 240.1025. The ^1^H and ^13^C spectral data were in accordance with those previously reported [29].

#### 3.4.14. (*R*)-2-(4-Phenyl-4,5-dihydrooxazol-2-yl)phenol **39**

Following the procedure of 3.4.13 using (*R*)-2-(2-(allyloxy)phenyl)-4-phenyl-4,5-dihydrooxazole **35** (3.10 g, 11.1 mmol), KOBu^t^ (2.74 g, 24.4 mmol) and *n*-butyllithium (9.8 cm^3^, 24.4 mmol) in PhMe (70 cm^3^) at rt for 2 h gave **39** (2.90 g, quant) as a dark-red oil which was used without further purification; δ_H_ (400 MHz) 12.14 (1H, br s, OH), 7.72 (1H, dd, *J* 7.8, 1.7, ArH), 7.46−7.40 (1H, m, ArH), 7.34−7.36 (2H, m, ArH), 7.34−7.28 (3H, m, ArH), 7.06 (1H, ddd, *J* 8.4, 1.1, 0.4, ArH), 6.91 (1H, ddd, *J* 7.8, 7.3, 1.2, ArH), 5.49 (1H, dd, *J* 10.1, 8.3, oxazoline NCH), 4.82 (1H, dd, *J* 10.1, 8.3, OC***H***H) and 4.27 (1H, t, *J* 8.3, OCH***H***); δ_C_ (100 MHz) 166.6 (C=N), 160.2 (C-O), 141.5 (C), 134.0 (CH), 129.0 (2CH), 128.4 (CH), 128.1 (CH), 126.7 (2CH), 118.9 (CH), 117.0 (CH), 110.4 (ArC), 74.3 (CH_2_) and 68.7 (CH); HRMS (NSI^+^): found 240.1021. C_15_H_14_NO_2_ (M + H) requires 240.1025. The ^1^H and ^13^C spectral data were in accordance with those previously reported [30].

#### 3.4.15. (*S*)-2-(4-Benzyl-4,5-dihydrooxazol-2-yl)phenol **40**

Following the procedure of 3.4.13 using (*S*)-2-(2-(allyloxy)phenyl)-4-benzyl-4,5-dihydrooxazole **36** (1.59 g, 5.42 mmol), KOBu^t^ (1.34 g, 11.9 mmol) and *n*-butyllithium (4.8 cm^3^, 11.9 mmol) in PhMe (30 cm^3^) at rt for 2 h gave **40** (1.37 g, quant) as a dark-red oil which was used without further purification; δ_H_ (400 MHz) 12.18 (1H, br s, OH), 7.62 (1H, dd, *J* 7.8, 1.7, ArH), 7.37 (1H, ddd, *J* 8.3, 7.3, 1.7, ArH), 7.32–7.27 (2H, m, ArH), 7.27–7.22 (3H, m, ArH), 7.01 (1H, dd, *J* 8.4, 1.1, ArH), 6.86 (1H, ddd, *J* 8.3, 7.3, 1.1, ArH), 4.65–4.58 (1H, m, OC***H***H), 4.38 (1H, dd, *J* 9.3, 8.5, OCH***H***), 4.13 (1H, dd, *J* 8.6, 7.4, NC***H***Ph), 3.10 (1H, dd, *J* 13.7, 6.4, C***H***HPh) and 2.81 (1H, dd, *J* 13.7, 7.5, CH***H***Ph); δ_C_ (100 MHz); 165.4 (C=N), 160.0 (C-O), 137.6 (C), 133.5 (CH), 129.3 (2CH), 128.7 (2CH), 128.1 (CH), 126.8 (CH), 118.7 (CH), 116.8 (CH), 110.7 (C), 71.3 (CH_2_), 66.8 (CH) and 42.0 (CH_2_); HRMS (NSI^+^): found 254.1171. C_16_H_16_NO_2_ (M + H) requires 254.1181. The ^1^H and ^13^C spectral data were in accordance with those previously reported [31].

#### 3.4.16. (*S*)-2-(4-Isopropyl-4,5-dihydrooxazol-2-yl)phenol **41**

Following the procedure of 3.4.13 using (*S*)-2-(-2(allyloxy)phenyl)-4-isopropyl-4,5-dihydrooxazole **37** (19.85 g, 80.9 mmol), KOBu^t^ (19.97 g, 178.0 mmol) and *n*-butyllithium (81 cm^3^, 178.0 mmol) in PhMe (450 cm^3^) at rt for 2 h gave, after purification via flash column chromatography (hexane/Et_2_O 9:1) at R_f_ 0.47, **41** (5.47 g, 33%) as a yellow oil; δ_H_ (400 MHz) ^1^H NMR 12.40 (1H, s, OH), 7.67 (1H, dd, *J* 7.7, 1.8, ArH), 7.39 (1H, ddd, *J* 8.4, 7.3, 1.8, ArH), 7.04 (1H, dd, *J* 8.4, 1.0, ArH), 6.89 (1H, ddd, *J* 7.7, 7.3, 1.0, ArH), 4.47–4.39 (1H, m OC***H***HCHN), 4.17–4.09 (2H, m OCH***H***C***H***N), 1.86–1.77 (1H, m, C***H***(CH_3_)_2_), 1.02 (3H, d, *J* 6.7, CHC***H_3_***CH_3_) and 0.95 (3H, d, *J* 6.7, CHCH_3_C***H_3_***); δ_C_ (100 MHz) 165.2 (C=N), 160.1 (C-O), 133.3 (CH), 128.1 (CH), 118.6 (CH), 116.8 (CH), 110.8 (C), 71.6 (CH_2_), 70.0 (CH), 33.1 (CH), 18.8 (CH_3_) and 18.7 (CH_3_); [α]_D_ −30.95, (*c* 1.076, CHCl_3_); (lit. [32] −35.4 (*c* 1.07, CHCl_3_)); HRMS (NSI^+^): found 206.1174. C_12_H_16_NO_2_ (M + H) requires 206.1181. The ^1^H and ^13^C spectral data were in accordance with those previously reported [32].

#### 3.4.17. (*S*)-4-Phenyl-2-(2-(1-phenylethoxy)phenyl)-4,5-dihydrooxazole **42**

To a stirred suspension of NaH (60% dispersion in mineral oil, 0.14 g, pre-washed with hexane, 3.5 mmol) in DMF (15 cm^3^) at rt was added (*S*)-2-(4-phenyl-4,5-dihydrooxazol-2-yl)phenol **38** (0.83 g, 3.5 mmol) and the mixture was stirred for 15 min before addition of (1-bromoethyl)benzene (0.48 cm^3^, 0.65 g, 3.5 mmol). After stirring for 18 h, the mixture was poured into water and extracted with CH_2_Cl_2_ followed by Et_2_O (×3). The combined organic extracts were washed with water (×5), dried and evaporated to give, after purification via column chromatography (hexane/Et_2_O 1:1) at R_f_ 0.32, **42** (0.67 g, 56%) as a pale-yellow oil as an inseparable 1:1 mixture of diastereomers; ν_max_/cm^−1^ 3383, 3028, 2978, 1638, 1599, 1491, 1449, 1067, 1028, 754, 698 and 542; δ_H_ (400 MHz) 7.74 (1H, dd, *J* 7.7, 1.5, ArH), 7.44–7.37 (4H, m, ArH), 7.37–7.32 (1H, m, ArH), 7.32−7.25 (3H, m, ArH), 7.25−7.20 (2H, m, ArH), 6.94−6.88 (1H, m, ArH), 6.83−6.79 (1H, m, ArH), 5.44–5.37 (2H, m, C***H***(CH_3_) and OC***H***HCHN)), 4.79 (1H, app ddd, *J* 10.2, 8.3, 2.8, OCH***H***CHN), 4.31−4.25 (1H, m, C***H***N) and 1.66 (3H, app t, *J* 6.4, OCHC***H***_3_); δ_C_ (100 MHz) 164.41 and 164.35 (C=N), 156.67 and 156.64 (C-O), 142.9 and 142.8 (C), 131.8 (CH), 131.1 (CH), 128.6 (2CH), 128.5 (2CH), 127.4 (CH), 126.7 (2CH), 125.6 (2CH), 120.43 and 120.36 (CH), 118.7 and 118.6 (C) 115.1 and 114.9 (CH), 77.1 and 76.9 (CH), 74.62 and 74.56 (CH_2_), 70.00 and 69.97 (CH) and 24.3 (CH_3_); HRMS (NSI^+^): found 344.1649. C_23_H_22_NO_2_ (M + H) requires 344.1651.

#### 3.4.18. (*R*)-4-Phenyl-2-(2-(1-phenylethoxy)phenyl)-4,5-dihydrooxazole **43**

Following the procedure of 3.4.17 using (*R*)-2-(4-phenyl-4,5-dihydrooxazol-2-yl)phenol **39** (0.53 g, 2.2 mmol), NaH (89 mg, 2.2 mmol) and (1-bromoethyl)benzene (0.30 cm^3^, 0.41 g, 2.2 mmol) in DMF (10 cm^3^) at rt for 18 h gave, after purification via column chromatography (hexane/Et_2_O 1:1) at R_f_ 0.32, **43** (0.19 g, 25%) as a pale yellow oil as an inseparable 1:1 mixture of diastereomers; ν_max_/cm^−1^ 3381, 3030, 1643, 1599, 1491, 1449, 1246, 1067, 1028, 750 and 698; δ_H_ (400 MHz); 7.74 (1H, app ddd, *J* 7.7, 1.9, 0.7, ArH), 7.45–7.20 (11H, m, ArH), 6.90 (1H, app tdd, *J* 7.5, 2.1, 1.0, ArH), 6.81 (1H, app ddd, *J* 8.5, 3.5, 1.0, ArH), 5.42−5.33 (2H, m, C***H***(CH_3_) and OC***H***HCHN)), 4.77 (1H, ddd, *J* 10.2, 8.3, 2.8, OCH***H***CHN), 4.27 (1H, td, *J* 8.1, 1.5, C***H***N) and 1.66 (3H, app t, *J* 6.4, CH_3_); δ_C_ (100 MHz) 164.34 and 164.29 (C=N), 156.61 and 156.57 (C-O), 142.8 and 142.7 (C), 131.8 (CH), 131.1 (CH), 128.5 (2CH), 128.4 (2CH), 127.3 (CH), 126.7 (2CH), 125.6 (2CH), 120.4 and 120.3 (CH), 118.6 and 118.5 (C), 115.1 and 114.9 (CH), 77.0 and 76.8 (CH), 74.6 and 74.5 (CH_2_), 69.94 and 69.91 (CH) and 24.3 (CH_3_); HRMS (NSI^+^): found 344.1649. C_23_H_22_NO_2_ (M + H) requires 344.1651.

#### 3.4.19. (*S*)-4-Benzyl-2-(2-(1-phenylethoxy)phenyl)-4,5-dihydrooxazole **44**

Following the procedure of 3.4.17 using (*R*)-2-(4-phenyl-4,5-dihydrooxazol-2-yl)phenol **40** (0.28 g, 1.1 mmol), NaH (44 mg, 1.1 mmol) and (1-bromoethyl)benzene (0.15 cm^3^, 0.20 g, 1.1 mmol) in DMF (10 cm^3^) at rt for 18 h gave, after purification via column chromatography (hexane/Et_2_O 1:1) at R_f_ 0.27, **44** (0.12 g, 31%) as a pale yellow oil as an inseparable 1:1 mixture of diastereomers; ν_max_/cm^−1^ 3028, 2928, 1643, 1599, 1491, 1450, 1240, 1066, 1028, 750, 698 and 519; δ_H_ (400 MHz) 7.68 (1H, dd, *J* 7.8, 1.8, ArH), 7.40 (2H, dt, *J* 8.0, 1.7, ArH), 7.33–7.27 (6H, m, ArH), 7.25–7.18 (3H, m, ArH), 6.89 (1H, tdd, *J* 7.5, 1.9, 1.0, ArH), 6.78 (1H, dd, *J* 8.4, 3.6, 1.0 ArH), 5.33 (1H, app quintet, *J* 6.2, OC***H***CH_3_), 4.60 (1H, ddddd, *J* 9.3, 8.4, 7.3, 5.4, 0.9, CHN), 4.35 (1H, dd, *J* 9.4, 8.4, OC***H***HCN), 4.13 (1H, ddd, *J* 8.5, 7.3, 1.4, OCH***H***CN), 3.25 (1H, dd, *J* 13.7, 5.3, CH_2_Ph), 2.80 (1H, ddd, *J* 13.8, 10.0, 8.4, CH_2_Ph) and 1.63 and 1.62 (3H, 2xd, *J* 6.4, OCHC***H***_3_); δ_C_ (100 MHz) 163.57 and 163.50 (C=N), 156.6 (C-O), 142.9 (C), 138.09 and 138.06 (C), 131.7 (CH), 131.0 (CH), 129.28 and 129.25 (2CH), 128.4 (4CH), 127.3 (CH), 126.3 (CH), 125.6 (2CH), 120.47 and 120.42 (CH), 118.9 and 118.8 (C), 115.4 and 115.3 (CH), 77.2 and 77.1 (CHN), 71.54 and 71.46 (CH_2_O), 67.7 (CH), 41.7 (CH_2_Ph) and 24.2 (CH_3_); HRMS (NSI^+^): found 358.1803. C_24_H_24_NO_2_ (M + H) requires 358.1807.

#### 3.4.20. (*S*)-4-Isopropyl-2-(2-(1-phenylethoxy)phenyl)-4,5-dihydrooxazole **45**

Following the procedure of 3.4.17 using (*S*)-2-(4-isopropyl-4,5-dihydrooxazol-2-yl)phenol **41** (1.0 g, 4.87 mmol), NaH (0.20 g, 4.87 mmol) and (1-bromoethyl)benzene (0.66 cm^3^, 0.89 g, 4.87 mmol) in DMF (20 cm^3^) at rt for 18 h gave, after purification via column chromatography (hexane/Et_2_O 1:1) at R_f_ 0.28, **45** (0.53 g, 35%) as a colourless oil and an inseparable 1:1 mixture of diastereomers; ν_max_/cm^−1^ 2959, 2928, 2872, 1643, 1599, 1490, 1479, 1449, 1236, 1067, 752 and 698; δ_H_ (400 MHz) 7.64 (1H, dt, *J* 7.6, 1.9, ArH), 7.43–7.36 (2H, m, ArH), 7.32–7.25 (2H, m, ArH), 7.24–7.14 (1H, m, ArH), 7.13–7.08 (1H, m, ArH), 6.86 (1H, tdd, *J* 7.6, 2.9, 1.0, ArH), 6.75 (1H, dd, *J* 8.4, 2.9, 1.0, ArH), 5.34 (1H, dq, *J* 9.3, 6.3,OCH), 4.43−4.34 (1H, m, OC***H***HCHN), 4.17–4.07 (2H, m, OCH***H****C**H***N), 1.93−1.83 (1H, m, C***H***(CH_3_)_2_), 1.62 (3H, app t, *J* 6.3, OCHC***H***_3_), 1.061 and 1.055 (3H, 2 × d, *J* 6.8, CHC***H***_3_CH_3_), and 0.989 and 0.983 (3H, 2 × d, *J* 6.8, CHCH_3_C***H***_3_); δ_C_ (100 MHz) 162.8 and 162.7 (C=N), 156.33 and 156.30 (C-O), 142.8 (C), 131.3 (CH), 130.80 and 130.78 (CH), 128.3 (2CH), 127.2 (CH), 125.48 and 125.45 (2CH), 120.24 and 120.16 (CH), 119.0 and 118.9 (C), 115.0 and 114.8 (CH), 76.8 and 76.6 (CHO), 72.41 and 72.36 (CH), 69.7 (CH_2_), 32.62 and 32.57 (CH), 24.16 and 24.13 (CH_3_), 18.55 and 18.48 (CH_3_) and 18.11 and 18.06 (CH_3_); HRMS (NSI^+^): found 310.1805. C_20_H_24_NO_2_ (M + H) requires 310.1807.

#### 3.4.21. Methyl 2-(1-phenylethoxy)benzoate 46

Following the procedure of 3.4.17 using methyl 2-hydroxybenzoate (17.04 cm^3^, 131.4 mmol), sodium hydride (5.27 g, 131.4 mmol) and (1-bromoethyl)benzene (17.93 cm^3^, 24.31 g, 131.4 mmol) in DMF (400 cm^3^) gave **46** (29.98 g, 89%) as a yellow oil; δ_H_ (300 MHz) 7.75 (1H, dd, *J* 7.8, 1.8, ArH), 7.46–7.19 (6H, m, ArH), 6.88 (1H, td, *J* 7.5, 1.0, ArH), 6.83–6.77 (1H, m, ArH), 5.37 (1H, q, *J* 6.4, OCH), 3.91 (3H, s, OCH_3_) and 1.66 (3H, d, *J* 6.4 CH***C***H_3_); δ_C_ (100 MHz) 166.2 (C=O), 156.7 (C-O), 142.3 (C), 132.4 (CH), 130.9 (CH), 128.1 (2CH), 127.0 (CH), 125.1 (2CH), 120.9 (C), 119.7 (CH), 114.8 (CH), 76.3 (OCHPh), 51.2 (O**C**H_3_) and 23.7 (CH_3_); The ^1^H spectral data were in accordance with those previously reported [33]. The ^13^C spectral data are reported for the first time.

#### 3.4.22. 2-(1-Phenylethoxy)benzoic Acid **47**

Following a literature procedure [34], NaOH (13.58 g, 339.45 mmol) was added to a stirred solution of methyl 2-(2-phenylethoxy)benzoate **46** (29.00 g, 113.15 mmol) in an EtOH/water mixture (9:1, 140 cm^3^) and the reaction mixture was heated at reflux for 3 h. After cooling to rt, the mixture was acidified to pH 1 by addition of 2 M HCl and extracted with PhMe (3 × 100 cm^3^). The combined organic layers were dried over MgSO_4_ and concentrated to give **47** (21.13 g, 77%) as a pale-yellow oil which slowly crystallised to give colourless crystals; mp 57−59 °C; ν_max_/cm^−1^ 3238, 2978, 1730, 1692, 1601, 1454, 1375, 1240, 1217, 1065, 750, 698 and 527; δ_H_ (400 MHz) 8.12 (1H, dd, *J* 7.7, 1.9, ArH), 7.36–7.31 (5H, m, ArH), 7.29−7.25 (1H, m, ArH), 6.98 (1H, td, *J* 7.7, 1.0, ArH), 6.90 (1H, dd, *J* 8.5, 1.0, ArH), 5.56 (1H, q, *J* 6.4, OCH) and 1.74 (3H, d, *J* 6.4, CH_3_); δ_C_ (100 MHz) 165.7 (C=O), 156.5 (C-O), 140.4 (C), 134.7 (CH), 133.5 (CH), 129.0 (2CH), 128.4 (CH), 125.3 (2CH), 122.0 (CH), 118.2 (C), 114.4 (CH), 79.1 (OC***H***Ph) and 23.9 (CH_3_); HRMS (NSI^−^): found 241.0871, C_15_H_13_O_3_ (M–H) requires 241.0865.

#### 3.4.23. *N*-((*S*)-1-Hydroxy-3-methylbutan-2-yl)-2-(1-phenylethoxy)benzamide **48**

To a stirred solution of 2-(1-phenylethoxy)benzoic acid **47** (21.00 g, 86.7 mmol) in PhMe (190 cm^3^) was added SOCl_2_ (12.57 cm^3^, 20.61 g, 173.4 mmol) and the mixture heated to reflux for 2 h then cooled to rt and concentrated.

To a stirred solution of the acid chloride prepared as above (12.65 g, 48.5 mmol) in CH_2_Cl_2_ at 0 °C was added Et_3_N (6.76 cm^3^, 4.91 g, 48.5 mmol) and (S)-2-amino-3-methylbutan-1-ol (5.00 g, 48.5 mmol) and the mixture stirred at rt for 18 h. The reaction mixture was poured into water (100 cm^3^) and extracted with CH_2_Cl_2_ (2 × 100 cm^3^), the combined organic layers were dried over MgSO_4_ and concentrated to give, after purification via flash column chromatography (Et_2_O/hexane 4:1) at R_f_ 0.18, 48 (9.13 g, 58%) as a colourless oil as an inseparable 1:1 mixture of diastereomers; ν_max_/cm^−1^ 3375, 2961, 2874, 1632, 1599, 1531, 1477, 1225, 1065, 932, 752, 700 and 583; δ_H_ (400 MHz) 8.40 (1H, d, *J* 8.3, NH), 8.18 (1H, dd, *J* 7.8, 1.9, ArH), 7.34 (4H, d, *J* 4.4, ArH), 7.32−7.26 (1H, m, ArH), 7.22 (1H, ddd, *J* 8.3, 7.3, 1.9, ArH), 6.96 (1H, dd, *J* 8.1, 7.3, 1.0, ArH), 6.78 (1H, dd, *J* 8.6, 1.0, ArH), 5.48 (1H, q, *J* 6.4, OCHCH_3_), 4.07 (1H, dtd, *J* 8.3, 5.9, 3.8, CHN), 3.84–3.73 (2H, m, CH_2_OH), 3.62 (1H, s, OH), 2.00 (1H, septet, *J* 6.8, (CH(CH_3_)_2_), 1.74 (3H, d, *J* 6.4, CH(CH_3_)Ph), 1.02 (3H, d, *J* 6.8, CH(CH_3_)(CH_3_)) and 0.99 (3H, d, *J* 6.8, CH(CH_3_)(CH_3_)); δ_C_ (125 MHz) 166.3 (C=O), 155.8 (C-O), 141.4 (C), 132.5 (CH), 132.2 (CH), 128.9 (2CH), 128.0 (CH), 125.2 (2CH), 121.5 (C), 121.0 (CH), 113.8 (CH), 77.3 (OCHPh), 64.2 (CH_2_OH), 57.5 (CHN), 29.2 (CH_3_), 24.2 (CH), 19.5 (CH_3_) and 18.6 (CH_3_); HRMS (NSI^+^): found 328.1911. C_20_H_27_NO_3_ (M + H) requires 328.1913

#### 3.4.24. (*S*)-4-Isopropyl-2-(2-(1-phenylethoxy)phenyl)-4,5-dihydrooxazole **45** from **48**

Following the procedure of 3.4.9 using *N*-((*S*)-1-Hydroxy-3-methylbutan-2-yl)-2-(1-phenylethoxy)benzamide **48** (9.13 g, 28.4 mmol), MsCl (2.61 cm^3^, 3.86 g, 33.7 mmol) and Et_3_N (8.62 cm^3^, 6.26 g, 62.0 mmol) in CH_2_Cl_2_ (80 cm^3^) gave **45** (7.37 g, 84%) as a colourless oil as an inseparable 1:1 mixture of diastereomers, spectroscopic data as in 3.4.20.

#### 3.4.25. 1-Phenylethyl 2-hydroxybenzoate **49**

To a stirred solution of 2-(1-phenylethoxy)benzoic acid **47** (1.00 g, 4.1 mmol) in CH_2_Cl_2_ (10 cm^3^) at 0 °C was added (COCl)_2_ (0.43 cm^3^, 0.63 g, 5.0 mmol) and the solution stirred at rt for 2 h. The reaction was concentrated and the crude residue, purified via flash column chromatography (hexane/EtOAc 4:1) to give, at R_f_ 0.83, **49** (0.51g, 51%) as colourless crystals; mp 57−60 °C; δ_H_ (400 MHz) 10.78 (1H, s, OH), 7.94 (1H, ddd, *J* 8.0, 1.8. 0.5, ArH), 7.47−7.41 (3H, m, ArH), 7.40−7.35 (2H, m, ArH), 7.34−7.29 (1H, m, ArH), 6.96 (1H, ddd, *J* 8.4, 1.2, 0.5, ArH), 6.89 (1H, ddd, *J* 8.2, 7.2, 1.2, ArH), 6.14 (1H, q, *J* 6.6, OC***H***CH_3_) and 1.69 (3H, d, *J* 6.6, OCHC***H***_3_); δ_C_ (100 MHz) 169.4 (C=O), 161.7 (C-O), 141.1 (C), 135.7 (CH), 129.9 (CH), 128.6 (2CH), 128.1 (CH), 126.0 (2CH), 119.1 (CH), 117.6 (CH), 112.7 (C), 73.6 (O***C***H) and 22.3 (CH_3_). The ^1^H spectral data were in accordance with those previously reported [16], and the ^13^C data are reported for the first time.

### 3.5. Reactivity of Oxazolines with Base

#### 3.5.1. (*S*)-3-Methyl-2-(((*S*)-3-methyl-3-phenylisobenzofuran-1(3*H*)-ylidene)amino)butan-1-ol **50**

To a stirred solution of (*S*)-4-isopropyl-2-(2-(1-phenylethoxy)phenyl)-4,5-dihydrooxazole **45** (62.0 mg, 0.2 mmol) in THF (2 cm^3^) at rt under a nitrogen atmosphere was added *n*-butyllithium (2.5 M in hexane, 0.18 cm^3^, 0.44 mmol) and the mixture stirred for 2 h. The reaction mixture was quenched by addition of sat. NH_4_Cl solution (10 cm^3^), extracted with Et_2_O (3 × 10 cm^3^), dried over MgSO_4_ and concentrated. The crude residue was purified via preparative TLC (Et_2_O/hexane 1:1) to give**,** at R_f_ 0.2, **27** (31.4 mg, 50%) as a pale-yellow oil; ν_max_/cm^−1^ 2959, 2924, 2872, 1763, 1686, 1466, 1269, 1026, 932, 766, 752, 698 and 583; δ_H_ (400 MHz) 7.90 (1H, m, ArH), 7.50–7.25 (8H, m), 3.90 (1H, m, CHN), 3.81−3.73 (2H, m, CH_2_O), 2.22 (1H, br s, OH), 1.98 (3H, s, C(Ph)C***H***_3_), 1.94 (1H, octet, C***H***(CH_3_)_2_), 1.01 (3 H, d, *J* 6.8, CH(C***H***_3_)CH_3_) and 0.96 (3 H, d, *J* 6.8, CH(CH_3_)C***H***_3_); δ_C_ (125 MHz) 151.1 (C=N), 142.3 (C), 131.8 (CH), 129.0 (C), 128.65 (2CH), 128.62 (CH), 128.0 (CH), 125.0 (C), 124.9 (2CH), 123.8 (CH), 121.5 (CH), 89.3 (C), 64.6 (CH_2_O), 63.9 (CHNH), 30.4 (***C***H_3_(C)Ph), 27.5 (CH), 19.7 (CH_3_) and 19.5 (CH_3_); HRMS (NSI^+^): found 310.1804. C_20_H_24_NO_2_ (M + H) requires 310.1807.

#### 3.5.2. 3-Methyl-3-phenylisobenzofuran-1(3*H*)-one **51**

To a stirred solution of (*S*)-4-isopropyl-2-(2-(1-phenylethoxy)phenyl)-4,5-dihydrooxazole **45** (62.0 mg, 0.2 mmol) in THF (2 cm^3^) at rt under N_2_ was added *n*-butyllithium (0.18 cm^3^, 0.44 mmol) and the solution stirred at rt for 2 h. The reaction was quenched by addition of sat. NH_4_Cl solution (5 cm^3^) and the layers separated. The aqueous layer was extracted with Et_2_O (3 × 10 cm^3^) and the organics dried over MgSO_4_, filtered, and concentrated. The crude residue was redissolved in a 1:1 THF/H_2_O mixture (2 cm^3^), 2 M HCl solution (0.2 cm^3^, 0.4 mmol) was added and the solution heated to reflux for 1 h. The reaction was cooled to rt and quenched with H_2_O (10 cm^3^) then transferred to a separating funnel. The mixture was extracted with pentane (3 × 10 cm^3^) and the combined organic layers dried over MgSO_4_ and concentrated to give, after purification via preparative TLC (hexane/Et_2_O 1:1) at R_f_ 0.24, **51** (19.0 mg, 43%, 2 steps) as a colourless oil; δ_H_ (400 MHz) 7.91 (1H, dt, *J* 7.5, 1.0, ArH), 7.66 (1H, td, *J* 7.5, 1.1, ArH), 7.52 (1H, td, *J* 7.5, 1.0, ArH), 7.48–7.43 (3H, m, ArH), 7.38–7.30 (3H, m, ArH) and 2.05 (3H, s, CH_3_); δ_C_ (100 MHz) 169.9 (C=O), 154.1 (C), 140.6 (C), 134.3 (CH), 129.1 (CH), 128.7 (2CH), 128.3 (CH), 125.8 (CH), 125.02 (2CH), 125.00 (C), 122.0 (CH), 87.5 (CO) and 27.2 (CH_3_); [α]_D_ +3.79 (*c* 0.376, CHCl_3_) (lit. [17] +69 (*c* 1.2, CHCl_3_)); HRMS (NSI^+^): found 225.0914. C_15_H_13_O_2_ (M + H) requires 225.0916. The ^1^H and ^13^C spectral data were in accordance with those previously reported [35].

#### 3.5.3. 2-Methyl-2-((3-methyl-3-phenylisobenzofuran-1(3*H*)-ylidene)amino)propan-1-ol **54**

To a stirred solution of 4,4-dimethyl-2-(2-(1-phenylethoxy)phenyl)-4,5-dihydrooxazole **9** (59.1 mg, 0.2 mmol) in THF (2 cm^3^) at rt under N_2_ was added *n*-butyllithium (0.26 cm^3^, 0.66 mmol) and the solution stirred at rt for 2 h. The reaction was quenched by addition of sat. NH_4_Cl solution (5 cm^3^) and the layers separated. The aqueous layer was extracted with Et_2_O (3 × 10 cm^3^) and the combined organic layers dried over MgSO_4_ and concentrated. The crude residue was purified via preparative TLC to give, at R_f_ 0.25, **54** (48.9 mg, 83%) as a slightly-yellow oil; ν_max_/cm^−1^ 2968, 2930, 2870, 1694, 1466, 1447, 1290, 1271, 1053, 1024, 934, 770, 750, 696, 679 and 583; δ_H_ (300 MHz) 7.80−7.74 (1H, m, ArH), 7.45−7.40 (3H, m, ArH), 7.38−7.34 (2H, m, ArH), 7.33−7.28 (2H, m, ArH), 7.19−7.13 (1H, m, ArH), 3.46 (2H, s, C***H***_2_OH), 2.00 (3H, s, CH_3_CPh), 1.42 (3H, s, CMe_2_) and 1.41 (3H, s, CMe_2_); δ_C_ (125 MHz) 150.1 (C=N), 147.0 (C), 128.57 (2CH), 128.53 (CH), 127.9 (CH), 127.5 (CH), 125.7 (CH), 125.0 (2CH), 121.4 (CH), 78.7 (CH_2_OH), 72.9 (CH_3_(***C***)O), 67.7 (C), 27.4 (***C***H_3_(C)O), 23.3 (CH_3_) and 23.0 (CH_3_); HRMS (ESI^+^) found 296.1639. C_19_H_22_NO_2_ (M + H) requires 296.1651.

The ^1^H NMR spectrum also showed signals suggesting the presence of around 20% of the spiro isomer (1*S*)-3,4′,4′-trimethyl-3-phenyl-3*H*-spiro[isobenzofuran-1,2′-oxazolidine] **54a** δ_H_ (300 MHz) (aliphatic signals only) 3.86 and 3.59 (2H, AB pattern, *J* 8.0, CH_2_O), 1.87 (3H, s, Me), 1.22 (3H, s, Me) and 0.74 (3H, s, Me).

### 3.6. Synthesis of Further More Highly Substituted Oxazolines

#### 3.6.1. *N*-((*S*)-2-Hydroxy-2,4-dimethylpentan-3-yl)-2-(1-phenylethoxy)benzamide **55**

To a stirred solution of 2-(1-phenylethoxy)benzoic acid **47** (4.0 g, 16.5 mmol) in CH_2_Cl_2_ (165 cm^3^) was added (COCl)_2_ (2.97 cm^3^, 4.40 g, 34.65 mmol) and two drops of DMF, and the mixture stirred at rt for 2 h, then cooled to rt and concentrated.

To a stirred solution of the resulting acid chloride (1.99 g, 7.62 mmol) in CH_2_Cl_2_ (20 cm^3^) at 0 °C was added Et_3_N (1.06 cm^3^, 0.77 g, 7.62 mmol) and (*S*)-3-amino-2,4-dimethylpentan-2-ol [36] (1.0 g, 7.62 mmol) and the mixture stirred at rt for 18 h. The reaction mixture was poured into H_2_O (50 cm^3^) and extracted with CH_2_Cl_2_ (2 × 50 cm^3^), the combined organic layers were dried over MgSO_4_ and concentrated. The crude residue was purified via flash column chromatography (Et_2_O/hexane 4:1) to give, at R_f_ 0.42, **55** (1.95 g, 72%) as a colourless oil as an inseparable mixture 2:1 of diastereomers whose spectra were sufficiently different to be separately identified; ν_max_/cm^−1^ 3385, 3356, 2972, 2957, 1690, 1624, 1533, 1479, 1219, 1177, 1161, 752, 702, 675 and 529; δ_H_ (400 MHz, major diastereomer) 8.52 (1H, d, *J* 9.8, NH), 8.24 (1H, ddd, *J* 7.8, 1.9, 1.0, ArH), 7.42−7.37 (3H, m, ArH), 7.32−7.26 (3H, m, ArH), 7.05−6.98 (1H, m, ArH), 6.83 (1H, d, *J* 8.2, ArH), 5.57 (1H, q, *J* 6.4, CH_3_C***H***OPh), 4.17 (1H, m, CHN), 2.25 (1H, octet, *J* 6.8, C***H***(CH_3_)_2_), 1.77 (3H, d, *J* 6.5, C***H***_3_CH(OAr)Ph), 1.36 (3H, s, *gem* dimethyl), 1.32 (3H, s, *gem* dimethyl) and 1.04−1.01 (6H, m, CH(C***H***_3_)_2_); δ_H_ (400 MHz, minor diastereomer) 8.44 (1H, d, *J* 10.0, NH), 8.24 (1H, ddd, *J* 7.8, 1.9, 1.0, ArH), 7.42−7.37 (3H, m, ArH), 7.32−7.26 (3H, m, ArH), 7.05−6.98 (1H, m, ArH), 6.80 (1H, d, *J* 8.3, ArH), 5.53 (1H, q, *J* 6.4, CH_3_C***H***OPh), 4.18 (1H, m, CHN), 2.27 (1H, octet, *J* 6.8, C***H***(CH_3_)_2_), 1.76 (3H, d, *J* 6.5, C***H***_3_CH(OAr)Ph), 1.35 (3H, s, *gem* dimethyl), 1.31 (3H, s, *gem* dimethyl) and 1.04−1.01 (6H, m, CH(C***H***_3_)_2_); δ_C_ (125 MHz, major diastereomer) 166.1 (C=O), 155.8 (C-O), 141.5 (C), 132.4 (CH), 132.3 (CH), 128.9 (2CH), 128.0 (CH), 125.5 (2CH), 121.73 (C), 120.93 (CH), 113.55 (CH), 76.6 (OCH), 73.95 (COH), 60.8 (NHCH), 29.32 (CH_3_), 28.42 (CH), 27.3 (CH_3_), 24.5 (CH_3_), 22.5 (CH_3_) and 17.5 (CH_3_); δ_C_ (125 MHz, minor diastereomer) 166.2 (C=O), 155.9 (C-O), 141.7 (C), 132.6 (CH), 132.4 (CH), 128.95 (2CH), 128.0 (CH), 125.2 (2CH), 121.67 (C), 120.98 (CH), 113.65 (CH), 77.0 (OCH), 73.92 (COH), 60.9 (NHCH), 29.26 (CH_3_), 28.38 (CH), 27.1 (CH_3_), 24.8 (CH_3_), 22.6 (CH_3_) and 17.6 (CH_3_); HRMS (NSI^+^): found 356.2223. C_22_H_31_NO_3_ (M + H) requires 356.2226.

#### 3.6.2. (*S*)-2-(4-Isopropyl-5,5-dimethyl-4,5-dihydrooxazol-2-yl)phenol **56**

A stirred solution of *N*-((*S*)-2-hydroxy-2,4-dimethylpentan-3-yl)-2-(1-phenylethoxy)benzamide **55** (0.80 g, 2.25 mmol) and MeSO_3_H (0.89 cm^3^, 1.31 g, 13.7 mmol) in CH_2_Cl_2_ (30 cm^3^) was heated to reflux using a Soxhlet extractor with CaH_2_ in the thimble for 12 h. The reaction mixture was cooled to rt, poured into H_2_O (10 cm^3^), extracted with CH_2_Cl_2_ (3 × 20 cm^3^), dried over MgSO_4_ and concentrated. The crude residue was purified via flash column chromatography (9:1 Hexane: EtOAc) to give, at R_f_ 0.45, **56** (480 mg, 93%) as a colourless oil; ν_max_/cm^−1^ 2972, 2872, 1640, 1491, 1350, 1261, 1236, 1069, 1040, 820, 752, 696, 681 and 540; δ_H_ (400 MHz) 12.63 (1H, br, OH), 7.60 (1H, dd, *J* 7.8, 1.8, ArH), 7.34 (1H, ddd, *J* 8.3, 7.3, 1.8, ArH), 6.98 (1H, dd, *J* 8.3, 1.1, ArH), 6.84 (1H, ddd, *J* 7.8, 7.3, 1.1, ArH), 3.50 (1H, d, *J* 8.6, C***H***-N), 1.87 (1H, d of septets, *J* 8.6, 6.6, C***H***(CH_3_)_2_), 1.54 (3H, s, CH_3_), 1.38 (3H, s, CH_3_), 1.11 (3H, d, *J* 6.6, CH_3_), 1.01 (3H, d, *J* 6.6, CH_3_); δ_C_ (100 MHz) 163.7 (C=N), 160.1 (C-O), 133.0 (CH), 127.9 (CH), 118.3 (CH), 116.6 (CH), 111.1 (C), 86.5 (C), 79.2 (CH-N), 28.93 (CH_3_), 28.88 (CH_3_), 21.2 (CH), 20.8 (CH_3_), 20.7 (CH_3_); HRMS (NSI^+^): found 234.1910. C_14_H_21_NO_2_ (M + H) requires *M*, 234.1914.

#### 3.6.3. (*S*)-4-Isopropyl-5,5-dimethyl-2-(2-(1-phenylethoxy)phenyl)-4,5-dihydrooxazole **57**

Following the procedure of 3.4.17 using (*S*)-2-(4-isopropyl-5,5-dimethyl-4,5-dihydrooxazol-2-yl)phenol **56** (390 mg, 1.67 mmol), NaH (67 mg, 1.67 mmol) and (1-bromoethyl)benzene (0.23 cm^3^, 0.31 g, 1.67 mmol) in DMF (10 cm^3^) at rt for 18 h gave, after purification via flash column chromatography (hexane/Et_2_O 3:1) at R_f_ 0.25, **57** (180 mg, 32%) as a slightly yellow oil as an inseparable 3:2 mixture of diastereomers; ν_max_/cm^−1^ 2972, 1643, 1601, 1489, 1450, 1242, 1042, 1069, 750 and 700; δ_H_ (400 MHz) 7.59 (1H, app dt, *J* 7.6, 2.0, ArH), 7.43−7.38 (2H, m, ArH), 7.34−7.28 (2H, m, ArH), 7.25−7.19 (1H, m, ArH), 7.18−7.14 (2H, m, ArH), 6.85 (1H, app tdd, *J* 7.5, 2.9, 1.0, ArH), 6.72 (1H, app dd, *J* 8.5, 1.0, ArH), 5.35 (1H, quintet, *J* 6.4, OC***H***CH_3_), 3.50 (1H, d, *J* 7.6, oxazoline CH), 1.93 (1H, app d of septets, *J* 7.5, 6.5, C***H***(CH_3_)_2_), 1.626 and 1.620 (3H, 2xd, *J* 6.4, OCHC***H***_3_), 1.54 (3H, s, *gem* dimethyl), 1.44 (3H, s, *gem* dimethyl), 1.182 and 1.180 (3H, 2xd, *J* 6.5, CH(C***H***_3_)CH_3_) and 1.06 (3H, d, *J* 6.5, CH(CH_3_)C***H***)_3_); δ_C_ (100 MHz) 161.7 (C=N), 156.41 and 156.38 (C-O), 143.0 (C), 131.2 (CH), 130.8 (CH), 128.42 (CH), 128.41 (CH), 128.3 (CH), 127.28 (CH), 127.27 (CH), 125.60 and 125.56 (CH), 125.3 (CH), 120.2 and 120.1 (CH), 119.7 and 119.6 (C) 114.6 and 114.4 (CH), 86.2 (C), 80.19 and 80.15 (CH), 76.6 and 76.4 (CH), 29.16 and 29.14 and 29.11 and 29.09 (CH and CH_3_), 24.37 and 24.34 (CH_3_), 21.2 (2CH_3_) and 20.3 (CH_3_); HRMS (NSI^+^): found 338.2120. C_22_H_29_NO_2_ (M + H) requires 338.2120.

#### 3.6.4. (*S*)-4-Isopropyl-2-(2-(1-phenylpropoxy)phenyl)-4,5-dihydrooxazole **58**

Following the procedure of 3.4.17 using (*S*)-2-(4-isopropyl-4,5-dihydrooxazol-2-yl)phenol **41** (1.0 g, 4.87 mmol), (1-bromopropyl)benzene [37] (970 mg, 4.87 mmol) and NaH (195 mg, 4.87 mmol) in DMF (20 cm^3^) gave, after purification via flash column chromatography (hexane/Et_2_O 4:1) at R_f_ 0.21, **58** (670 mg, 43%) as a pale-yellow oil as an inseparable 1:1 mixture of diastereomers; ν_max_/cm^−1^ 3061, 3030, 2961, 2932, 2876, 1645, 1601, 1450, 1491, 1250, 1238, 1040, 976, 748, 733 and 700; δ_H_ (400 MHz) 7.62 (1H, app ddd, *J* 7.6, 1.9, 1.0, ArH), 7.39−7.27 (4H, m, ArH), 7.27−7.20 (1H, m, ArH), 7.16 (1H, app ddd, *J* 8.4, 7.4, 1.8, ArH), 6.85 (1H, app tdd, *J* 7.5, 2.1, 1.0, ArH), 6.70 (1H, app ddd, *J* 8.4, 3.1, 1.0, ArH), 5.12 and 5.11 (1H, 2x t, *J* 7.2, PhC***H***(O)CH_2_CH_3_), 4.46−4.38 (1 H, m, OC***H***HCHN), 4.20−4.10 (2 H, m, OCH***H****C**H***N), 2.07−1.79 (3H, m, C***H***(CH_3_)_2_ and CH(OAr)C***H***_2_CH_3_), 1.07 (3H, d, *J* 6.8, CH(C***H***_3_)CH_3_), 1.01 (3H, d, *J* 6.4, CH(CH_3_)C***H***_3_) and 0.98 (3H, t, *J* 7.2, CH_2_C***H***_3_); δ_C_ (100 MHz) 163.2 and 163.1 (C=N), 156.73 and 156.65 (C-O), 141.5 and 141.4 (C), 131.4 (CH), 130.97 and 130.92 (CH), 128.35 and 128.34 (2CH), 127.3 (CH), 126.15 and 126.13 (2CH), 120.06 and 120.04 (CH), 118.87 and 118.84 (C), 114.48 and 114.43 (CH), 81.7 and 81.6 (OCH), 72.5 (CH), 70.0 and 69.9 (CH_2_), 32.78 and 32.76 (CH), 31.37 and 31.33 (CH_2_), 18.75 and 18.73 (CH_3_), 18.22 and 18.17 (CH_3_) and 9.65 and 9.63 (CH_3_); HRMS (ESI^+^): found 324.1950. C_21_H_26_NO_2_ (M + H) requires 324.1950.

#### 3.6.5. 2-(1-Bromo-2-methylpropyl)thiophene

A solution of 2-methyl-1-(thiophen-2-yl)-propan-1-ol [38] (1.00 g, 6.4 mmol) and pyridine (cat.) in CH_2_Cl_2_ (30 cm^3^) was stirred at 0 °C while PBr_3_ (0.30 cm^3^, 0.87 g, 3.2 mmol) was added dropwise. After 1 h, aqueous Na_2_CO_3_ was added dropwise and the mixture was warmed to RT before being separated, with the aqueous layer being further extracted with CH_2_Cl_2_, and the combined organic extracts were dried and evaporated to give the title product (1.30 g, 93%) as a dark brown oil, which was used without further purification; δ_H_ (400 MHz) 7.27 (1H, ddd, *J* 5.1, 1.3, 0.5, ArH), 7.03 (1H, ddd, *J* 3.5, 1.3, 0.5, ArH), 6.91 (1H, dd, *J* 5.1, 3.5, ArH), 5.10 (1H, d, *J* 7.5, ArC***H***(Br)*^i^*Pr), 2.29 (1H, d of septets, *J* 7.6, 6.6, C***H***(CH_3_)_2_), 1.18 (3 H, d, *J* 6.6, CH_3_) and 0.99 (3 H, d, *J* 6.6, CH_3_).

#### 3.6.6. (*S*)-4-Isopropyl-2-(2-(2-methyl-1-(thiophen-2-yl)propoxy)phenyl)-4,5-dihydrooxazole **59**

Following the procedure of 3.4.17 using (*S*)-2-(4-isopropyl-4,5-dihydrooxazol-2-yl)phenol **41** (1.0 g, 4.87 mmol), 2-(1-bromo-2-methylpropyl)thiophene (1.07 g, 4.87 mmol) and NaH (195 mg, 4.87 mmol) in DMF (20 cm^3^) gave, after purification via flash column chromatography (hexane/Et_2_O 4:1) at R_f_ 0.13, **59** (70 mg, 4%) as a pale-yellow oil as an inseparable 1:1 mixture of diastereoisomers; ν_max_/cm^−1^ 2959, 2930, 2872, 1645, 1601, 1491, 1352, 1250, 1233, 1040, 1001, 961, 750 and 696; δ_H_ (400 MHz) 7.63 (1H, app dd, *J* 7.6, 1.8, ArH), 7.24−7.18 (2H, m, ArH), 6.98−6.94 (1H, m, ArH), 6.94−6.85 (2H, m, ArH), 6.85−6.81 (1H, m, ArH), 5.21 and 5.20 (1H, 2x d, *J* 5.4, ArC***H***O(Ar), 4.46−4.37 (1H, m, OC***H***HCHN), 4.18−4.08 (2H, m, OCH***H****C**H***N), 2.26−2.17 (1H, m, C***H***(CH_3_)_2_), 1.95−1.85 (1H, m, oxazoline C***H***(CH_3_)_2_), 1.10−1.05 (6H, m, CH(C***H***_3_)_2_) and 1.01−0.96 (6H, m, oxazoline CH(C***H***_3_)_2_); δ_C_ (100 MHz) 163.33 and 163.25 (C=N), 156.7 and 156.6 (4ry, ArC-O), 143.2 and 143.1 (C), 131.5 (CH), 131.1 and 131.0 (CH), 126.3 (CH), 125.45 and 125.41 (CH), 124.6 (CH), 120.4 (CH), 119.0 (C), 114.2 (CH), 81.8 and 81.7 (***C***H(OAr), 72.5 (oxazoline CH), 70.1 and 70.0 (CH_2_), 35.6 and 35.5 (CH), 32.8 (CH), 18.86 and 18.85 (CH_3_), 18.4 (CH_3_), 18.22 and 18.17 (CH_3_) and 18.07 and 18.02 (CH_3_); HRMS (ESI^+^): found 366.1489. C_20_H_2_NaNO_25_S (M + Na) requires 366.1504.

#### 3.6.7. (*S*)-4-Isopropyl-2-(2-((1-phenyl-3-(trimethylsilyl)prop-2-yn-1-yl)oxy)phenyl)-4,5-dihydrooxazole **60**

Following the procedure of 3.4.17 using (*S*)-2-(4-isopropyl-4,5-dihydrooxazol-2-yl)phenol **41** (1.0 g, 4.87 mmol), (3-bromo-3-phenylprop-1-yn-1-yl)trimethylsilane [39] (1.30 g, 4.87 mmol) and NaH (195 mg, 4.87 mmol) in DMF (20 cm^3^) gave, after purification via flash column chromatography (hexane/Et_2_O 4:1) at R_f_ 0.26, **60** (210 mg, 11%) as a pale-yellow oil as an inseparable 1:1 mixture of diastereomers; ν_max_/cm^−1^ 2959, 2899, 1645, 1601, 1493, 1450, 1354, 1248, 1036, 908, 841, 754, 731 and 696; δ_H_ (400 MHz) 7.87 (1H, ddd, *J* 7.7, 2.8, 1.8, ArH), 7.84−7.79 (2H, m, ArH), 7.52−7.44 (4H, m, ArH), 7.42−7.37 (1H, m, ArH), 7.16 (1H, tt, *J* 7.5, 1.2, ArH), 6.03 and 6.01 (1H, s, ArC***H***O(Ar)), 4.49−4.41 (1H, m, OC***H***HCHN), 4.24−4.12 (2H, m, OCH***H****C**H***N), 1.99−1.89 (1H, m, C***H***(CH_3_)_2_, 1.11 (3H, d, *J* 6.7, CH(C***H***_3_)CH_3_), 1.03 and 1.02 (3H, 2 × d, *J* 6.7, CH(CH_3_)C***H***_3_) and 0.27 (9H, s, SiMe_3_); δ_C_ (100 MHz) 162.4 and 162.1 (C=N), 156.1 and 156.0 (C), 137.7 and 137.6 (C), 131.4 (CH), 130.9 (CH), 128.40 (CH), 128.2 (2CH), 127.2 (2CH), 121.8 (CH), 119.7 and 119.6 (C), 117.6 and 117.5 (CH), 102.39 and 102.35 (≡***C***-CHOAr), 94.02 and 93.98 (≡C-SiMe_3_), 72.7 and 72.5 (CH), 72.1 and 72.0 (CH), 69.8 and 69.7 (CH_2_), 32.68 and 32.61 (CH), 18.8 (CH_3_), 18.2 and 18.1 (CH_3_) and –0.4 (SiMe_3_); HRMS (ESI^+^): found 414.1850. C_24_H_29_NaNO_2_Si (M + Na) requires 414.1865.

#### 3.6.8. (*S*)-4-Isopropyl-2-(2-((1-phenylbut-3-en-1-yl)oxy)phenyl)-4,5-dihydrooxazole **61**

Following the procedure of 3.4.17 using (*S*)-2-(4-isopropyl-4,5-dihydrooxazol-2-yl)phenol **41** (1.0 g, 4.87 mmol), (1-bromobut-3-en-1-yl)benzene [40] (1.03 g, 4.87 mmol) and NaH (195 mg, 4.87 mmol) in DMF (20 cm^3^) gave, after purification via flash column chromatography (hexane/Et_2_O 4:1) at R_f_ 0.19, **61** (50 mg, 3%) as a pale-yellow oil as an inseparable 1:1 mixture of diastereomers; ν_max_/cm^−1^ 2956, 1643, 1601, 1582, 1450, 1491, 1385, 1250, 1040, 750 and 700; δ_H_ (400 MHz) 7.63 (1H, app dt, *J* 7.6, 1.5, ArH), 7.40−7.34 (2H, m, ArH), 7.34−7.28 (2H, m, ArH), 7.24–7.20 (1H, m, ArH), 7.19−7.14 (1H, m, ArH), 6.86 (1H, app tdd, *J* 7.5, 18.8, 1.0, ArH), 6.69 (1H, app ddd, *J* 8.4, 3.9, 1.0, ArH), 5.90 (1H, ddt, *J* 17.2, 10.3, 7.0, C***H***=CH_2_), 5.20 (1H, q, *J* 6.2, OC***H***Ph), 5.08−5.01 (2H, m, CH=C***H***_2_), 4.45−4.38 (1H, m, OC***H***HCHN), 4.19−4.10 (2H, m, OCH***H***C***H***N), 2.84−2.72 (1H, m, C***H***HCH=CH_2_), 2.67−2.57 (1H, m, CH***H***CH=CH_2_), 1.96−1.86 (1H, m, C***H***(CH_3_)_2_), 1.07 (3H, d, *J* 6.8, CH(C***H***_3_)CH_3_) and 1.00 (3H, d, *J* 6.8, CH(CH_3_)C***H***_3_)); δ_C_ (100 MHz) 163.2 and 163.1 (C=N), 156.5 and 156.4 (ArC-O), 140.94 and 140.88 (ArC), 134.1 and 134.0 (CH=), 131.45 and 131.43 (CH), 131.05 and 130.96 (CH), 128.41 and 128.39 (2CH), 127.5 (CH), 126.20 and 126.17 (2CH), 120.3 (CH), 118.96 and 118.90 (C), 117.48 and 117.42 (=CH_2_), 114.64 and 114.61 (CH), 80.4 and 80.3 (CH), 72.5 (CH), 70.0 and 69.9 (CH_2_), 42.81 and 42.78 (CH_2_), 32.78 and 32.74 (CH), 18.8 (CH_3_) and 18.24 and 18.18 (CH_3_); HRMS (ESI^+^): found 358.1768. C_22_H_25_NaNO_2_ (M + Na) requires 358.1783.

### 3.7. Synthesis and Reactivity of a Chiral Amide

#### 3.7.1. (*S*)-2-Amino-3-phenylpropan-1-ol

Following a literature procedure [41], a solution of sodium borohydride (21.89 g, 0.579 mol) in EtOH/H_2_O (1:1, 100 cm^3^) was added dropwise to a stirred solution of (*S*)-phenylalanine methyl ester hydrochloride (36.69 g, 0.170 mol) in EtOH/H_2_O (1:1, 330 cm^3^). The reaction mixture was heated at reflux for 18 h before being concentrated in vacuo. The residue was adjusted to pH 14 by addition of 2 M NaOH and extracted with EtOAc (5 × 100 cm^3^). The combined organic extracts were dried and evaporated and the crude residue was recrystallised (EtOAc/hexane) to give the title compound (10.50 g, 41%) as pale yellow crystals; mp 87–90 °C; (lit. [42] 91–93 °C); ${\left[a\right]}_{D}^{25}$–24.2 (c 1.011, EtOH); (lit. [43] ${\left[a\right]}_{D}^{20}$–24.1 (c 1, EtOH)); δ_H_ (400 MHz) 7.33–7.28 (2 H, m, Ph), 7.25–7.17 (3 H, m, Ph), 3.65 (1 H, dd, *J* 10.8, 4.0, C***H***HOH), 3.40 (1 H, dd, *J* 10.8, 7.2, CH***H***OH), 3.16–3.10 (1 H, m, NCH), 2.80 (1 H, dd, *J* 13.6, 5.2, PhC***H***H), 2.55 (1 H, dd, *J* 13.6, 8.6, PhCH***H***) and 2.34 (3 H, br s, NH_2_ and OH). The ^1^H NMR spectral data were in accordance with those previously reported [41].

#### 3.7.2. (*S*)-1-Methoxy-3-phenylpropan-2-amine **62**

Following a literature procedure [20], a solution of (*S*)-2-amino-3-phenylpropan-1-ol (17.52 g, 0.116 mol) in dry THF (150 cm^3^) was added dropwise to a stirred suspension of sodium hydride (60% in mineral oil, 8.40 g, 0.210 mol) in dry THF (110 cm^3^). The reaction mixture was stirred at rt for 4 h before methyl iodide (6.9 cm^3^, 15.73 g, 0.111 mol) was added and the reaction mixture was stirred for a further 18 h before being quenched by addition of water (150 cm^3^) and extracted with Et_2_O (3 × 100 cm^3^). The combined organic layers were washed with brine (100 cm^3^) before being dried and evaporated. The crude residue was purified by Kugelrohr distillation (120 °C/20 Torr; (lit. [44] bp 52 °C/0.1 Torr)) to give **62** (16.20 g, 88%) as a pale yellow oil; ${\left[a\right]}_{D}^{25}$–9.8 (c 1.809, CHCl_3_); (lit. [45] ${\left[a\right]}_{D}^{22}$–10.3 (c 1.80, CHCl_3_)); δ_H_ (300 MHz) 7.34–7.28 (2 H, m, Ph), 7.25–7.18 (3 H, m, Ph), 3.39–3.33 (1 H, m, NCH), 3.37 (3 H, s, CH_3_), 3.27–3.18 (2 H, m, CH_2_OMe), 2.78 (1 H, dd, *J* 13.5, 5.0, PhC***H***H), 2.55 (1 H, dd, *J* 13.5, 7.7, PhCH***H***) and 1.46 (2 H, br s, NH_2_). The ^1^H NMR spectral data were in accordance with those previously reported [44].

#### 3.7.3. (*S*)-2-(Benzyloxy)-*N*-(1-methoxy-3-phenylpropan-2-yl)benzamide **63**

A solution of 2-(benzyloxy)benzoyl chloride **25** (2.17 g, 8.80 mmol) in toluene (20 cm^3^) was added dropwise to a stirred 0 °C solution of (*S*)-1-methoxy-3-phenylpropan-2-amine **62** (1.54 g, 9.32 mmol) and triethylamine (1.5 cm^3^, 1.09 g, 10.8 mmol) in toluene (20 cm^3^). Once the addition was complete, the reaction mixture was allowed to warm to rt over 1 h before being washed with 2 M HCl (50 cm^3^), 2 M NaOH (50 cm^3^) and brine (50 cm^3^). The organic layer was dried and evaporated and the crude residue purified by Kugelrohr distillation (205 °C/5 Torr) to give **63** (2.18 g, 66%) as a viscous orange oil; ${\left[a\right]}_{D}^{25}$–44.2 (c 0.104, CH_2_Cl_2_); ν_max_/cm^−1^ 3392, 3029, 2925, 1652, 1533, 1382, 1296, 1228, 1123, 1004, 916, 857, 750 and 699; δ_H_ (400 MHz) 8.25–8.22 (1 H, m, ArH), 8.19 (1 H, d, *J* 7.6, NH), 7.44–7.38 (5 H, m, ArH), 7.26–7.02 (8 H, m, ArH), 5.15 and 5.13 (2 H, AB pattern, *J*_AB_ 11.2, OCH_2_Ph), 4.49–4.41 (1 H, m, NCH), 3.27–3.17 (2 H, m, CH_2_OMe), 3.17 (3 H, s, CH_3_) and 2.80 (2 H, d, *J* 7.2, CHC***H***_2_Ph); δ_C_ (100 MHz) 164.6 (C=O), 156.8 (C–O), 138.2 (C), 135.6 (C), 132.6 (CH), 132.3 (CH), 129.4 (2CH), 128.8 (2CH), 128.6 (CH), 128.2 (2CH), 128.0 (2CH), 126.2 (CH), 121.8 (C), 121.5 (CH), 112.5 (CH), 72.2 (OCH_2_), 71.2 (OCH_2_), 58.7 (OCH_3_), 50.5 (NCH) and 37.2 (CH***C***H_2_Ph); HRMS (ESI^+^): found 398.1713. C_24_H_25_NaNO_3_ (M + Na) requires 398.1727.

#### 3.7.4. Reaction of **63** to Give (*R*)-3-Phenylphthalide **64**

Under a nitrogen atmosphere, *sec*-butyllithium (1.4 M in cyclohexane, 1.2 cm^3^, 1.68 mmol) was added dropwise to a stirred solution of (*S*)-2-(benzyloxy)-*N*-(1-methoxy-3-phenylpropan-2-yl)benzamide **63** (0.1884 g, 0.50 mmol) in dry THF (5 cm^3^). After stirring at rt for 2 h, the reaction mixture was quenched by addition of sat. aq. NH_4_Cl (20 cm^3^) and extracted with Et_2_O (3 × 20 cm^3^). The combined organic layers were washed with 2 M NaOH (20 cm^3^) and water (20 cm^3^), before being dried and evaporated to give an inseparable 68:32 mixture of diastereomers. On standing for 5 weeks at rt, an intramolecular cyclisation occurred, to give, after purification by preparative TLC (SiO_2_, Et_2_O/hexane 1:1) at R_f_ 0.70, **64** (72.9 mg, 69%) as a pale yellow solid; ${\left[a\right]}_{D}^{25}$–18.1 (*c* 0.061, CHCl_3_); (lit. [46] ${\left[a\right]}_{D}^{25}$–48.6 (*c* 2.1, CHCl_3_)).

## 4. Conclusions

Further study on the base-induced Wittig rearrangement of 2-(2-benzyloxy)aryloxazolines has revealed its limitations. Aza- and thia-analogues are unsuccessful, as competing processes intervene and the rearrangement is suppressed completely in both 2-benzyloxy-3-pyridyl-oxazolines and -amides. In attempts to carry out the reaction with chiral oxazolines, the valine-derived 4-isopropyloxazoline group is most effective but although the rearrangement takes place with high diastereoselectivity, removal of the oxazoline is accompanied by significant racemisation leading to a final phthalide product of low e.e. More encouraging results have been achieved using a phenylalanine-derived secondary alkoxy amide directing group and this should be the focus for further studies.

## Supplementary Materials

The following supporting information can be downloaded at: https://www.mdpi.com/article/10.3390/molecules27103186/s1, Figures S1–S65: NMR spectra of new compounds.
