# Supplementary material for: Further Studies on the [1,2]-Wittig Rearrangement of 2-(2-Benzyloxy)aryloxazolines
Source: Molecules. 2022 May 17;27(10):3186. doi: 10.3390/molecules27103186 (PMC9144599; doi:10.3390/molecules27103186)

Further studies on the [1,2]-Wittig rearrangement of 2-(2-benzyloxy)aryloxazolines

R. Alan Aitken*, Andrew D. Harper and Ryan A. Inwood

EaStCHEM School of Chemistry, University of St Andrews, North Haugh, St Andrews, Fife, KY16 9ST, UK.

Supplementary Material	Figure
^{1}H NMR and DEPTQ ^{13}C NMR spectra of **17**	S1, S2
^{1}H NMR and DEPTQ ^{13}C NMR spectra of **22**	S3, S4
^{1}H NMR and DEPTQ ^{13}C NMR spectra of **23**	S5, S6
^{1}H NMR and DEPTQ ^{13}C NMR spectra of **24**	S7, S8
^{1}H NMR and DEPTQ ^{13}C NMR spectra of **26**	S9, S10
^{1}H NMR and DEPTQ ^{13}C NMR spectra of **27**	S11, S12
^{1}H NMR and DEPTQ ^{13}C NMR spectra of **28**	S13, S14
DEPTQ ^{13}C NMR spectrum of **29**	S15
^{1}H NMR and DEPTQ ^{13}C NMR spectra of **30**	S16, S17
^{1}H NMR and DEPTQ ^{13}C NMR spectra of **31**	S18, S19
^{1}H NMR and DEPTQ ^{13}C NMR spectra of **32**	S20, S21
^{1}H NMR and DEPTQ ^{13}C NMR spectra of **33**	S22, S23
^{1}H NMR and DEPTQ ^{13}C NMR spectra of **34**	S24, S25
^{1}H NMR and DEPTQ ^{13}C NMR spectra of **35**	S26, S27
^{1}H NMR and DEPTQ ^{13}C NMR spectra of **36**	S28, S29
^{1}H NMR and DEPTQ ^{13}C NMR spectra of **37**	S30, S31

^{1}H NMR and DEPTQ ^{13}C NMR spectra of **42** S32, S33
^{1}H NMR and DEPTQ ^{13}C NMR spectra of **43** S34, S35
^{1}H NMR and DEPTQ ^{13}C NMR spectra of **44** S36, S37
^{1}H NMR and DEPTQ ^{13}C NMR spectra of **45** S38, S39
DEPTQ ^{13}C NMR spectrum of **46** S40
^{1}H NMR and DEPTQ ^{13}C NMR spectra of **47** S41, S42
^{1}H NMR and DEPTQ ^{13}C NMR spectra of **48** S43, S44
DEPTQ ^{13}C NMR spectrum of **49** S45
^{1}H NMR and DEPTQ ^{13}C NMR spectra of **50** S46, S47
^{1}H NMR and DEPTQ ^{13}C NMR spectra of **54** S48, S49
^{1}H NMR and DEPTQ ^{13}C NMR spectra of **55** S50, S51
^{1}H NMR and DEPTQ ^{13}C NMR spectra of **56** S52, S53
^{1}H NMR and DEPTQ ^{13}C NMR spectra of **57** S54, S55
^{1}H NMR and DEPTQ ^{13}C NMR spectra of **58** S56, S57
^{1}H NMR and DEPTQ ^{13}C NMR spectra of **59** S58, S59
^{1}H NMR and DEPTQ ^{13}C NMR spectra of **60** S60, S61
^{1}H NMR and DEPTQ ^{13}C NMR spectra of **61** S62, S63
^{1}H NMR and DEPTQ ^{13}C NMR spectra of **63** S64, S65

Figure S1. 500 MHz DEPTQ ^1H NMR spectrum of **17**

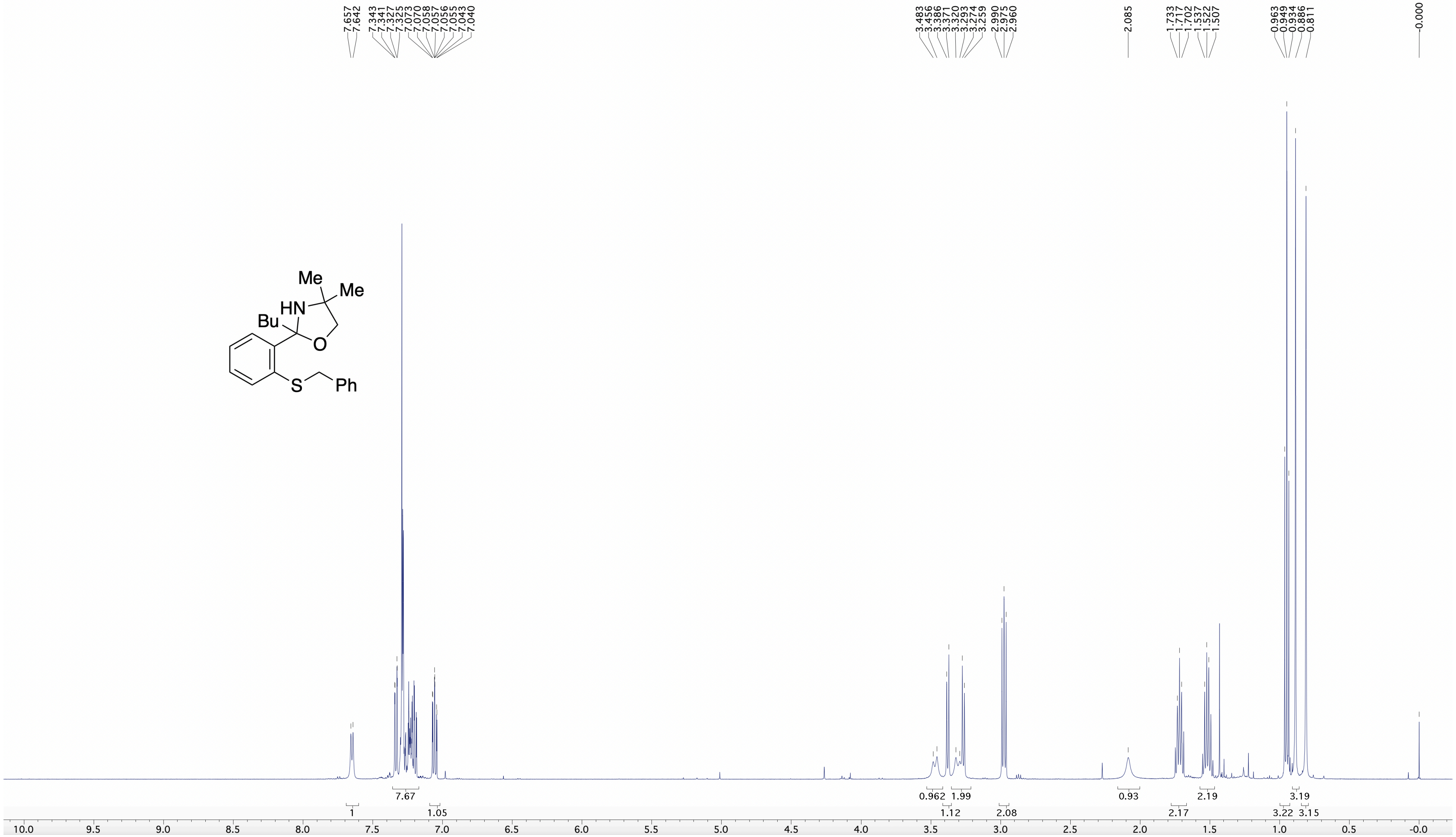

Figure S2. 125 MHz DEPTQ ^{13}C NMR spectrum of **17**

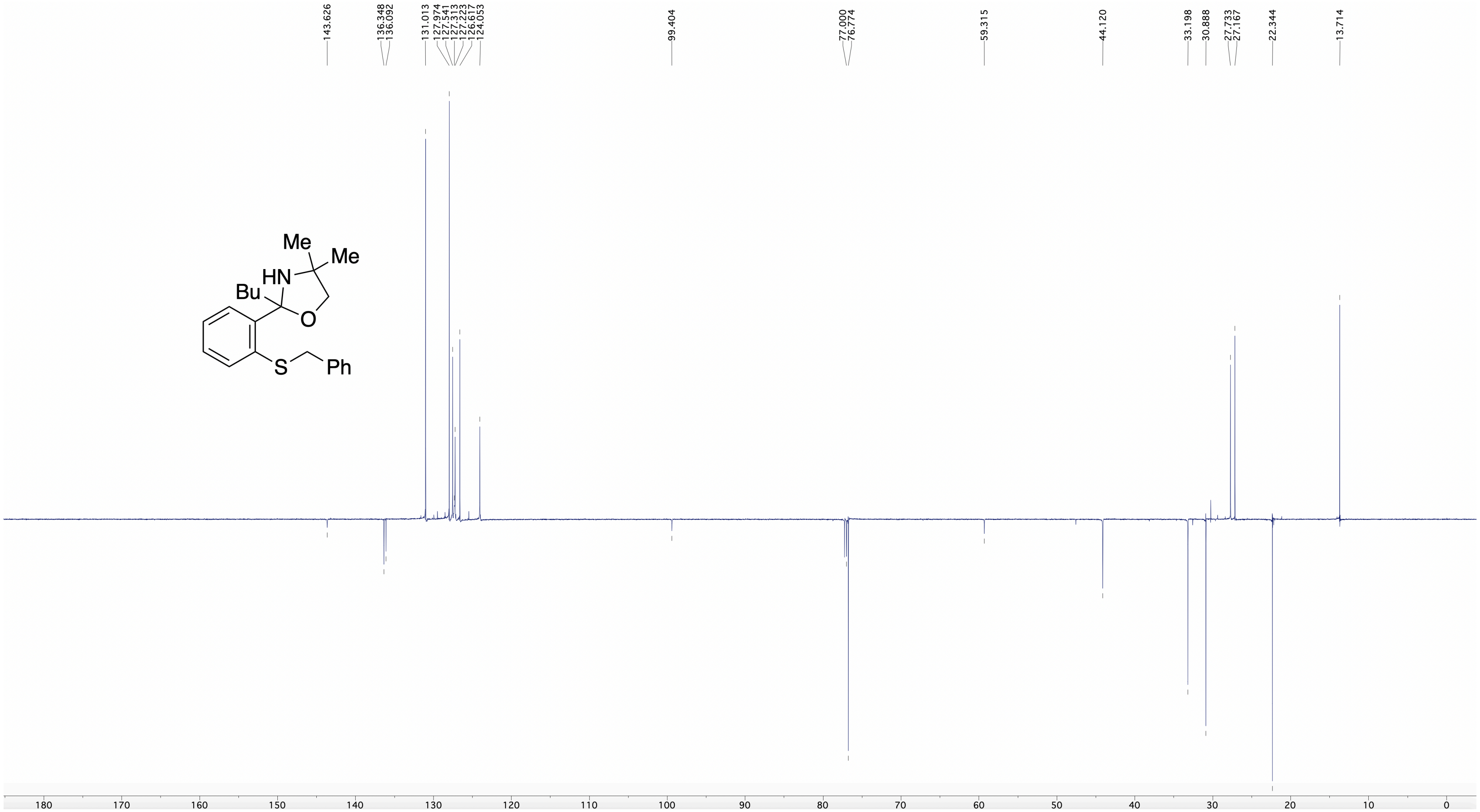

Figure S3. 500 MHz ^{1}H NMR spectrum of **22**

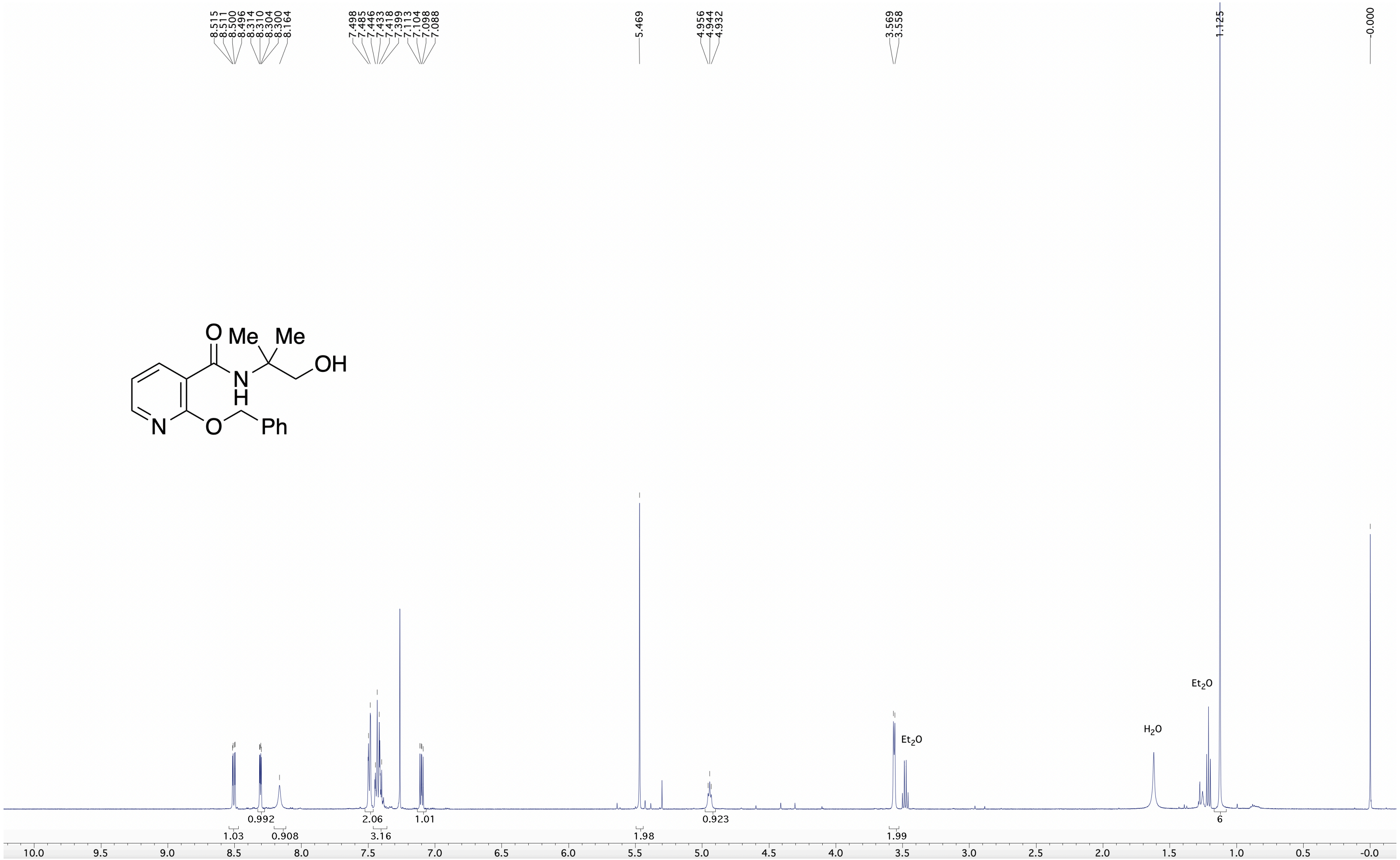

Figure S4. 125 MHz DEPTQ ^{13}C NMR spectrum of **22**

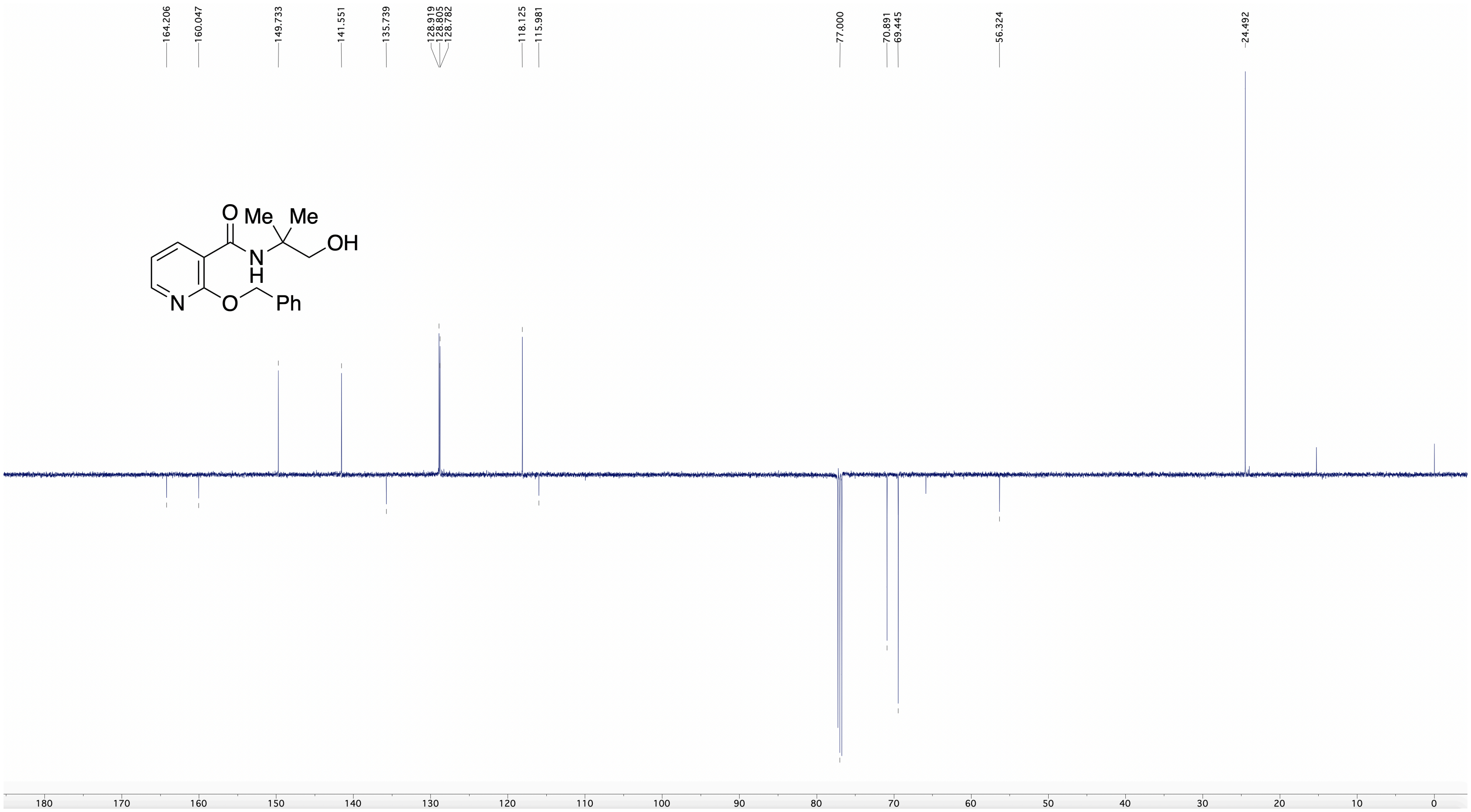

Figure S5. 500 MHz ^{1}H NMR spectrum of **23**

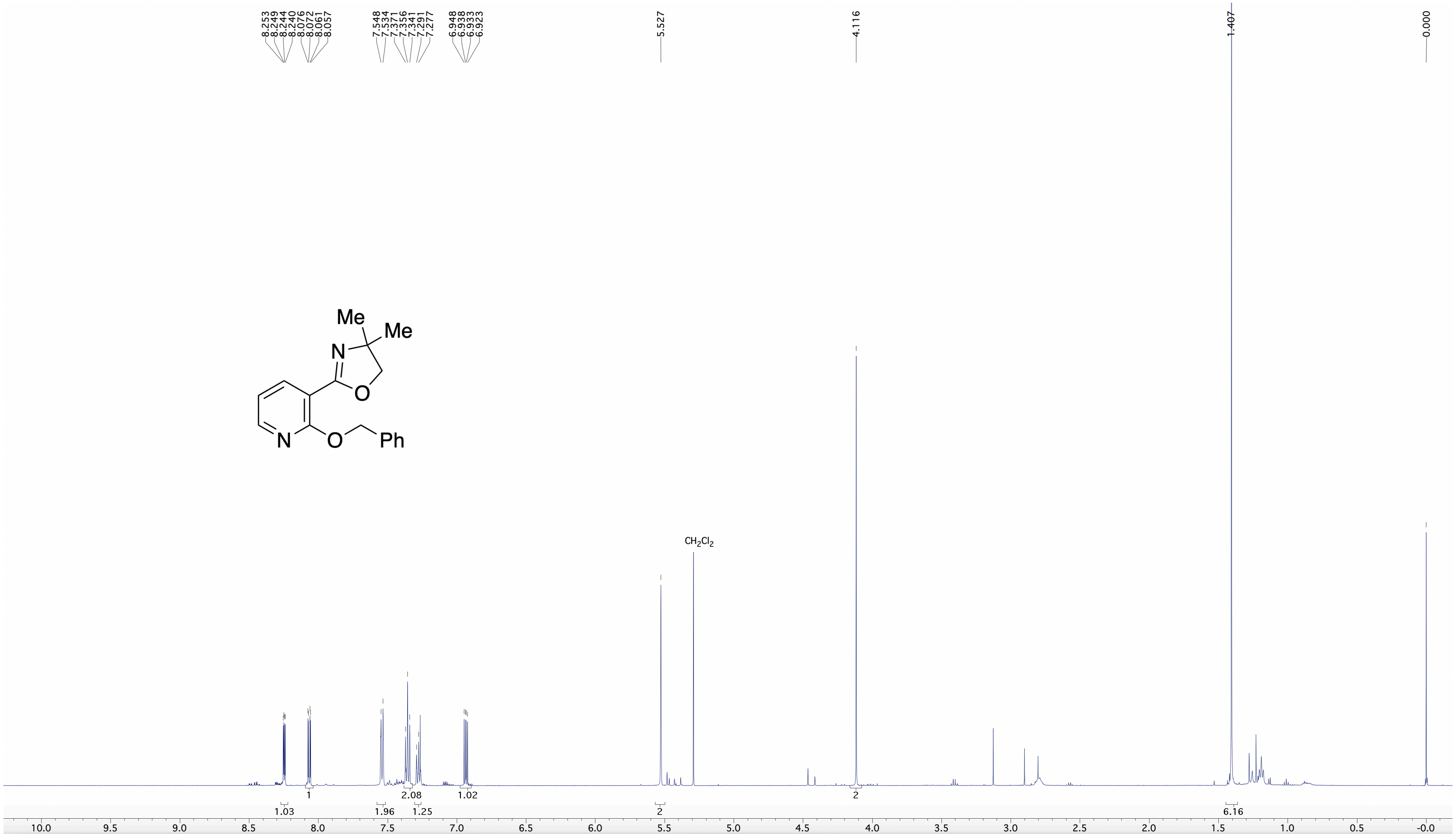

Figure S6. 125 MHz DEPTQ ^{13}C NMR spectrum of **23**

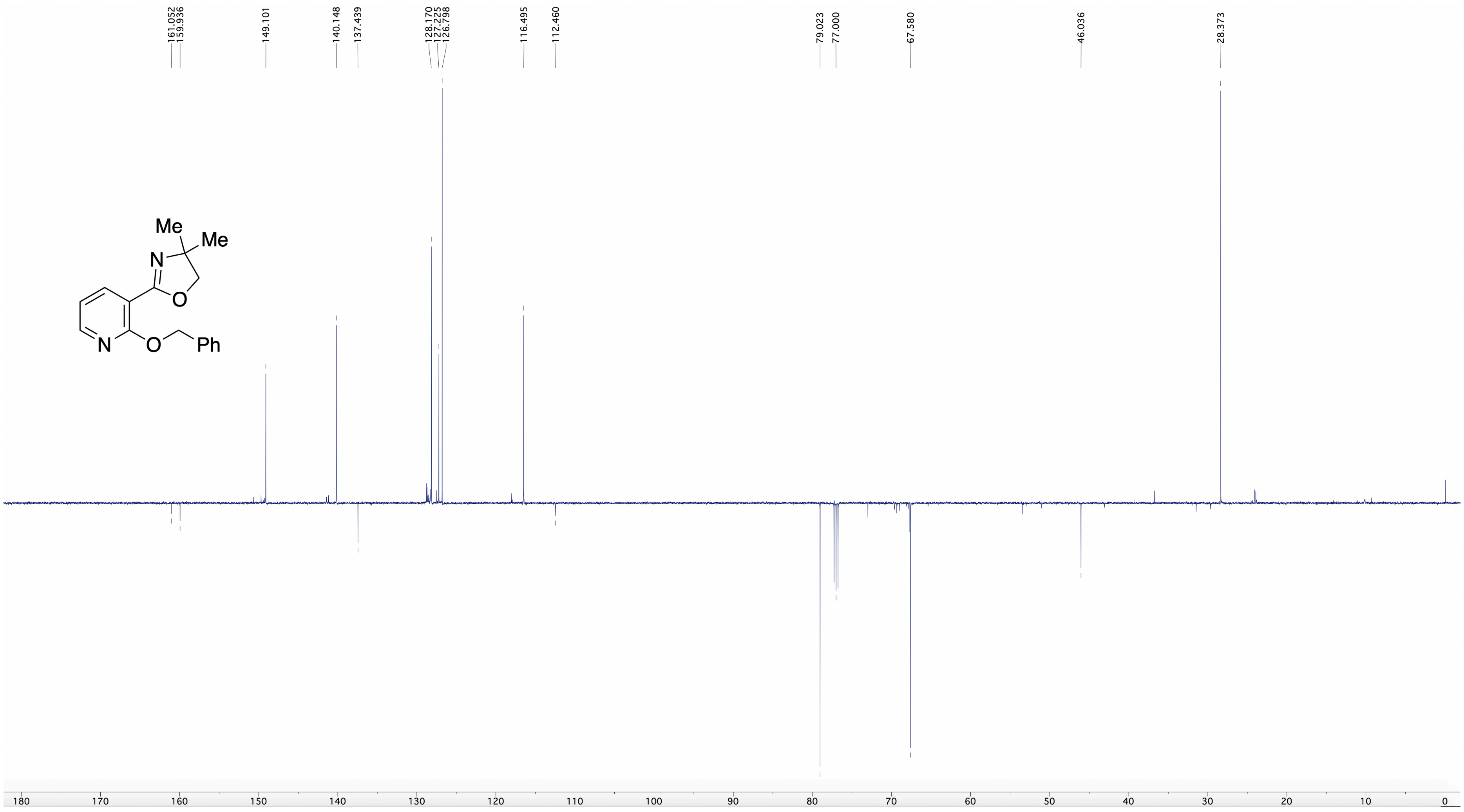

Figure S7. 500 MHz ^{1}H NMR spectrum of **24**

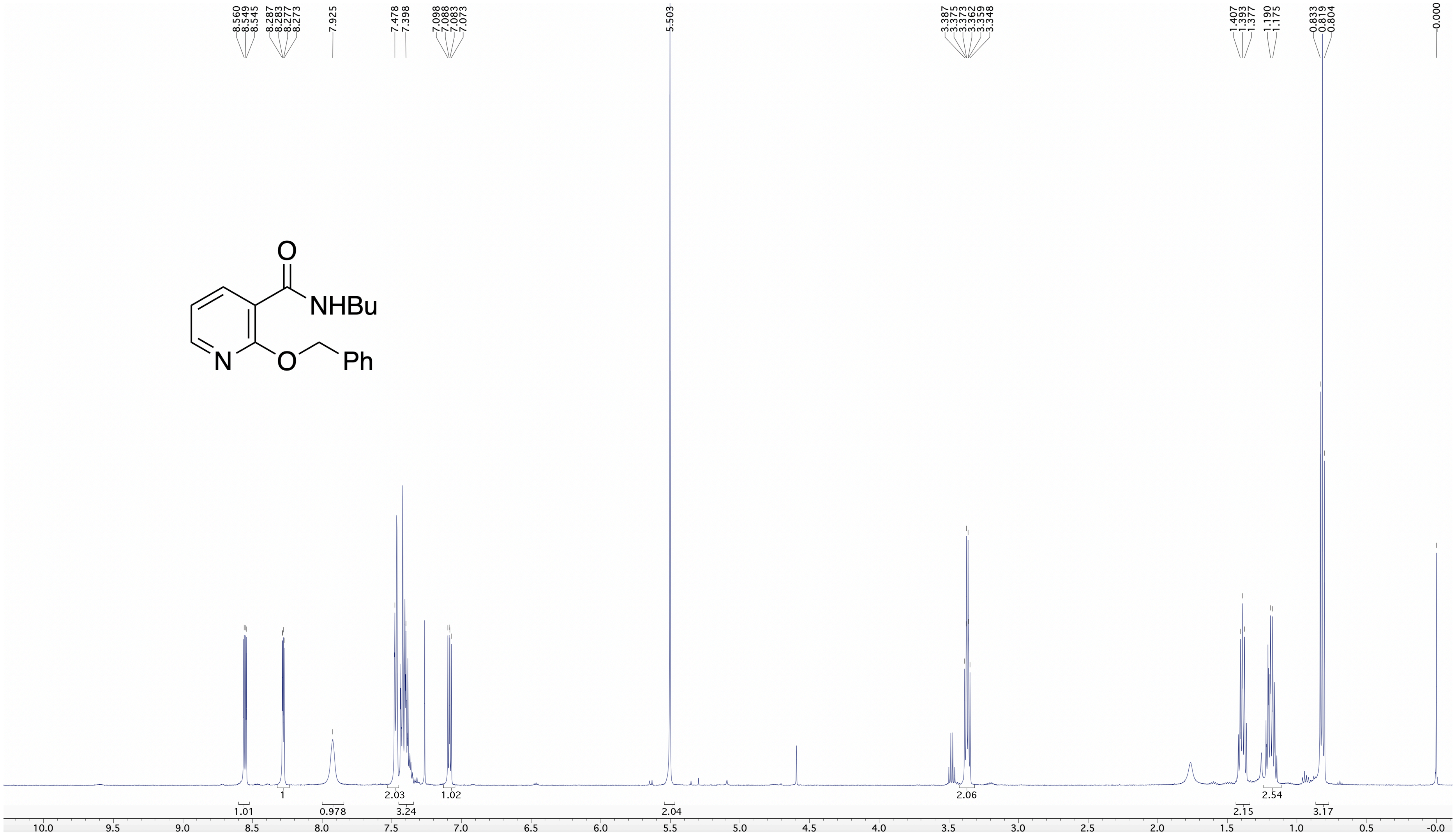

Figure S8. 125 MHz DEPTQ ^{13}C NMR spectrum of **24**

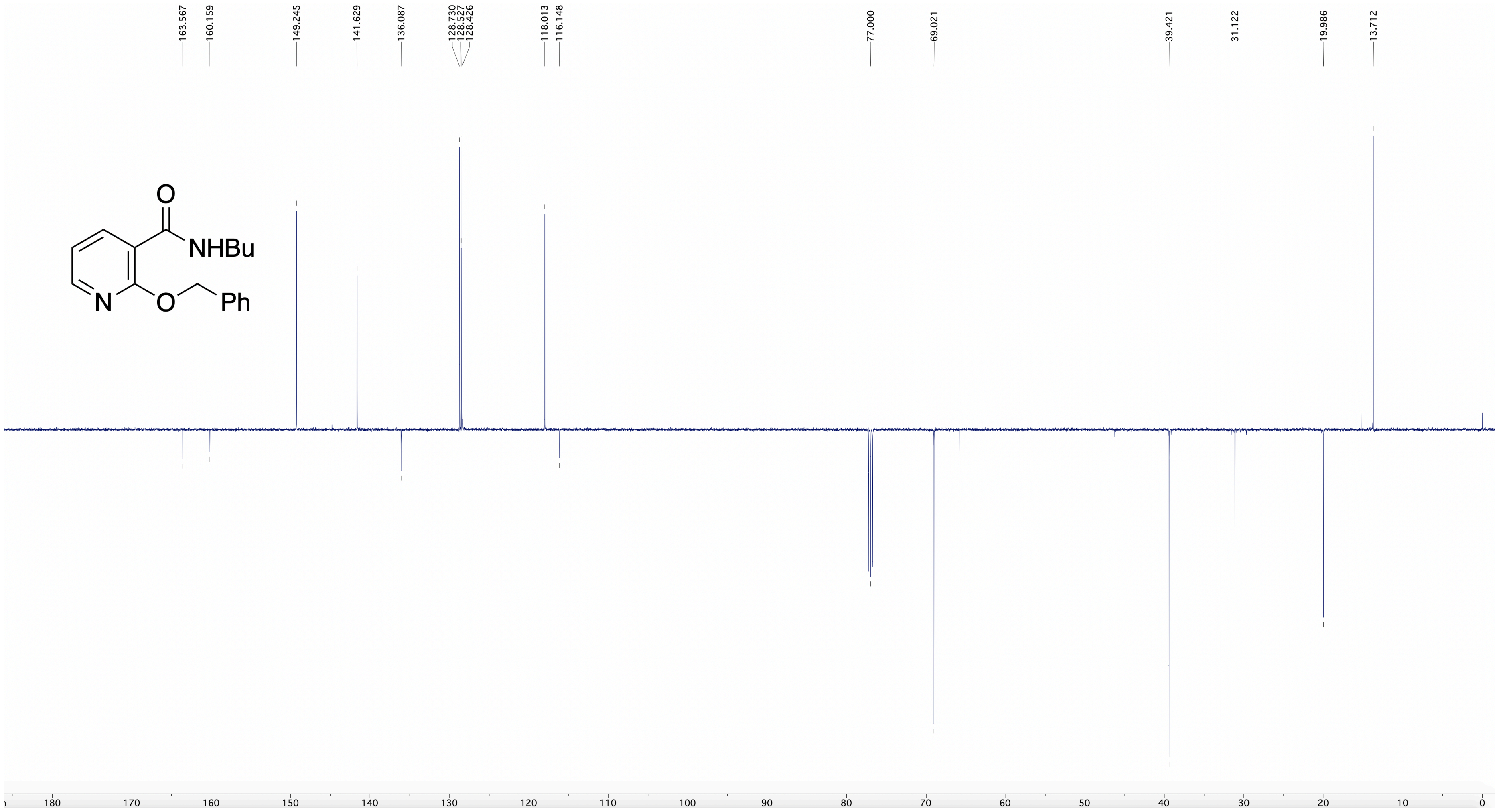

Figure S9. 400 MHz ^{1}H NMR spectrum of **26**

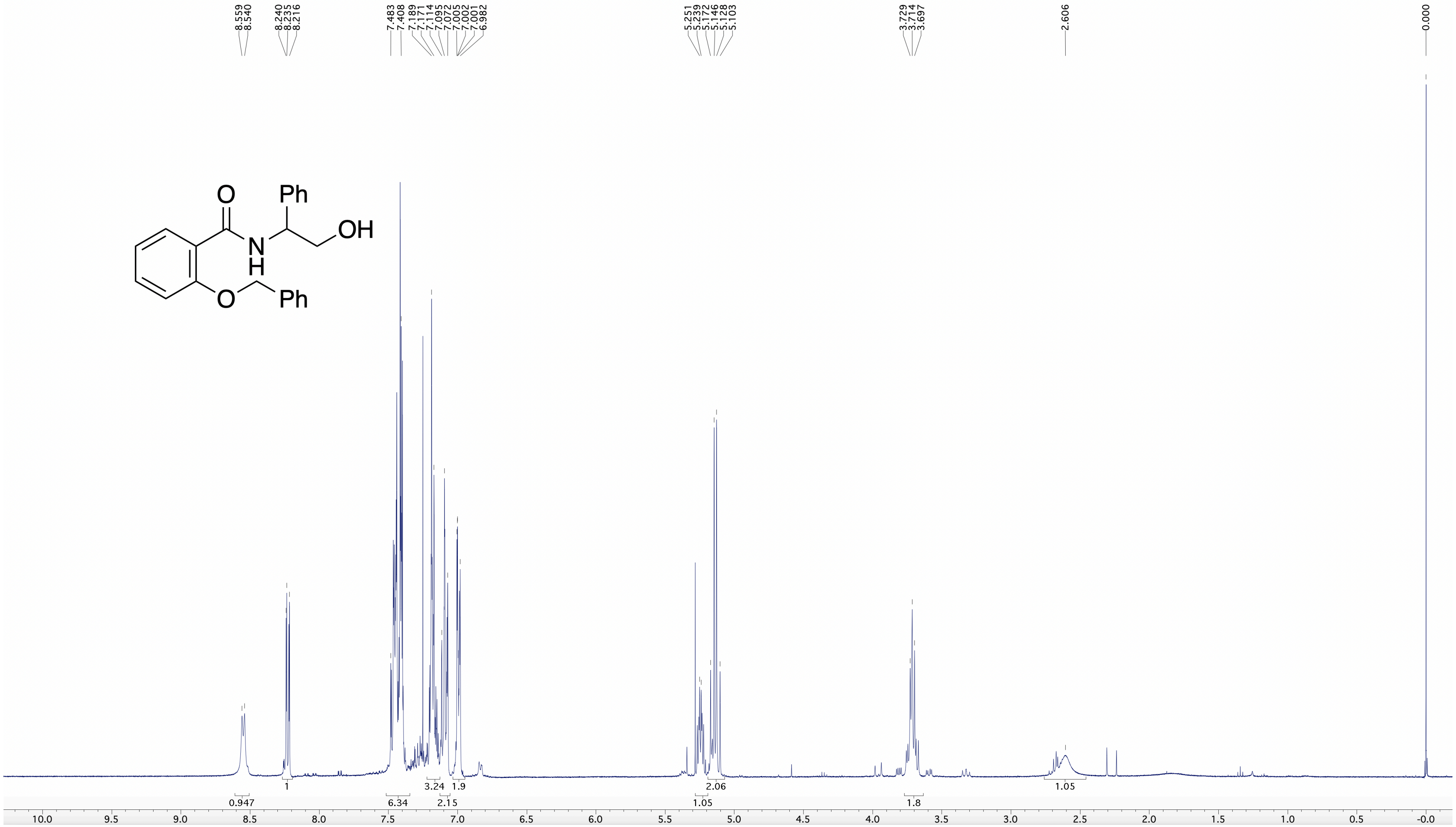

Figure S10. 100 MHz DEPTQ ^{13}C NMR spectrum of **26**

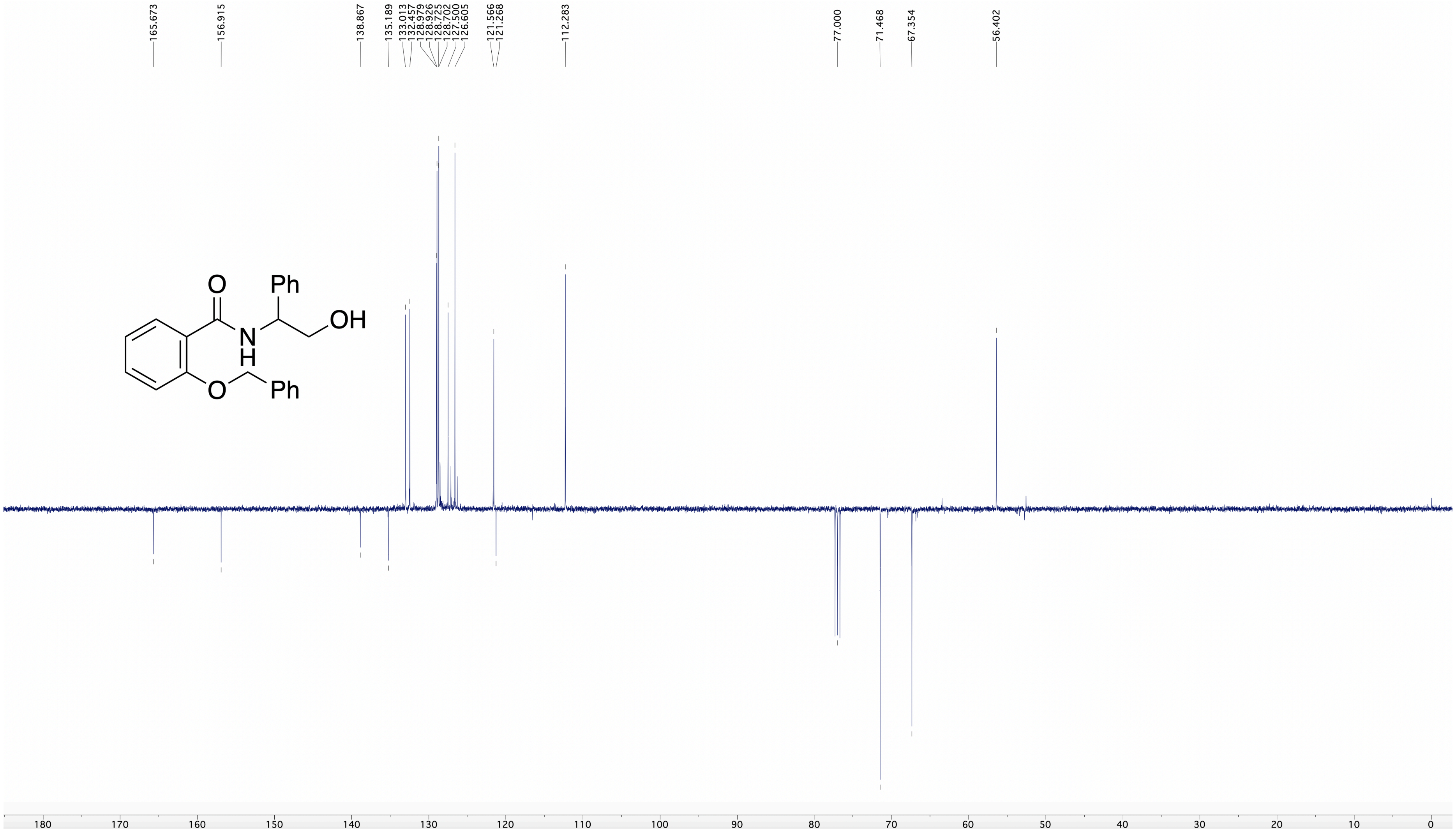

Figure S11. 500 MHz ^1H NMR spectrum of **27**

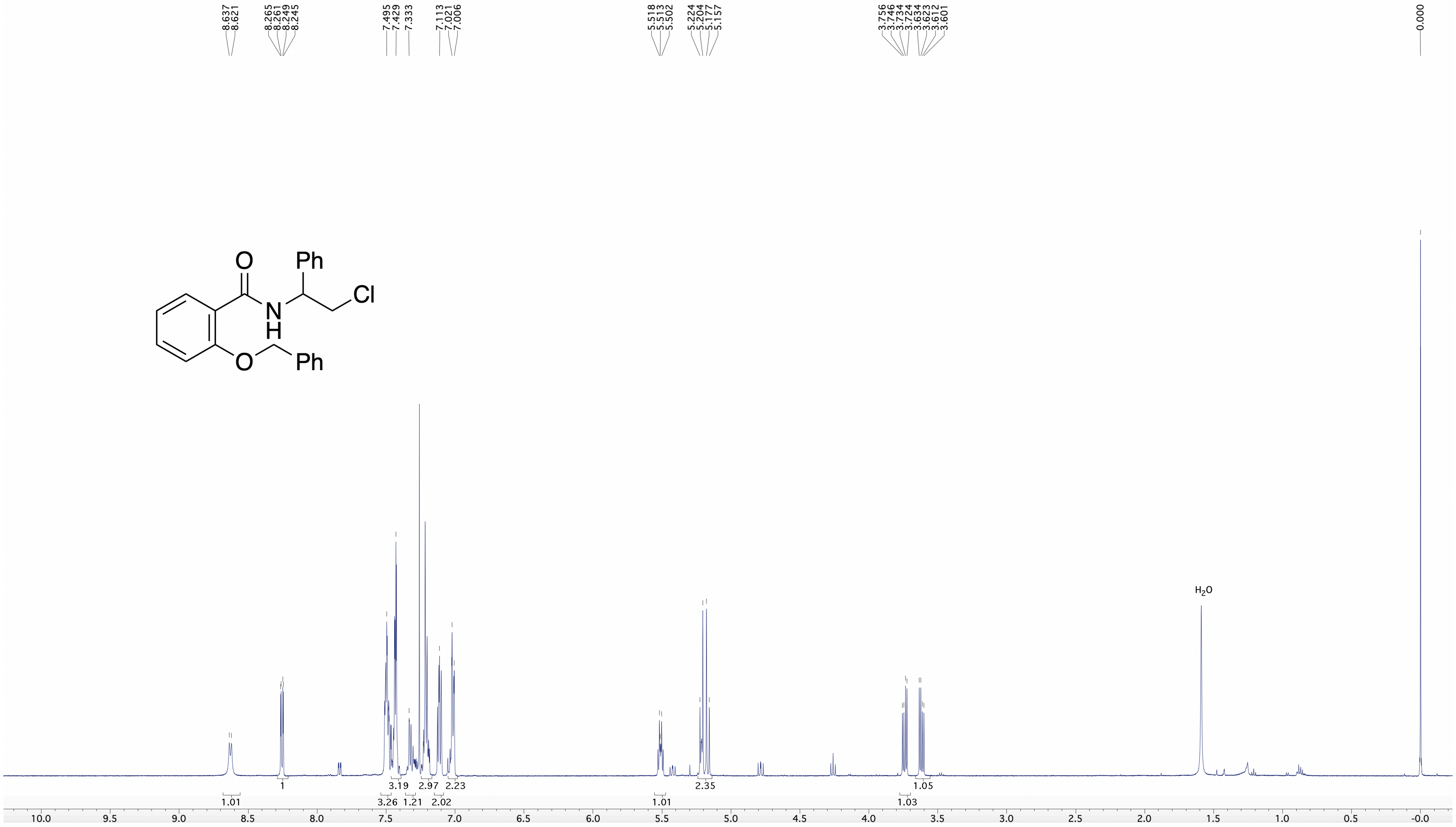

Figure S12. 125 MHz DEPTQ ^{13}C NMR spectrum of **27**

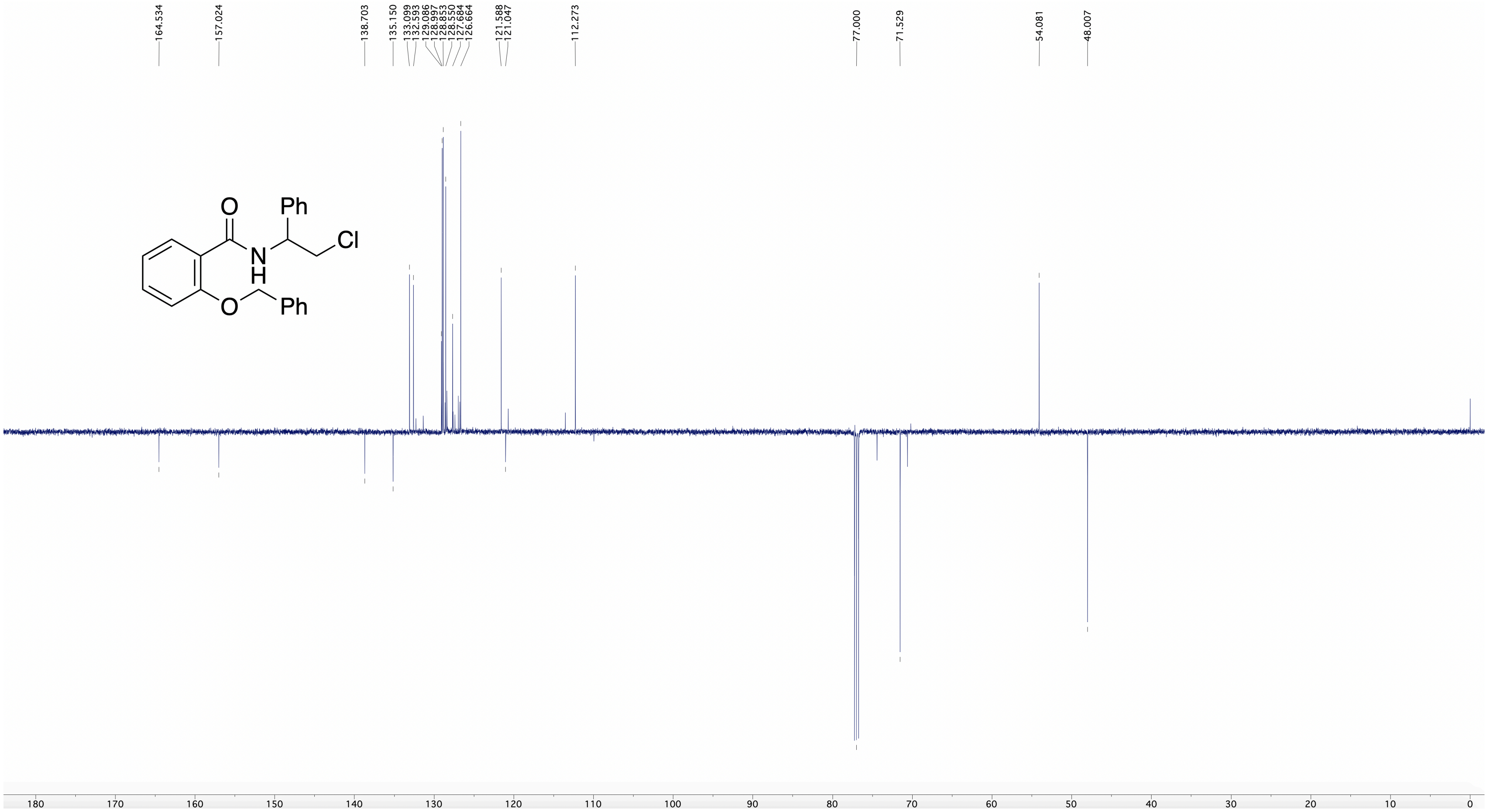

Figure S13. 500 MHz ^{1}H NMR spectrum of **28**

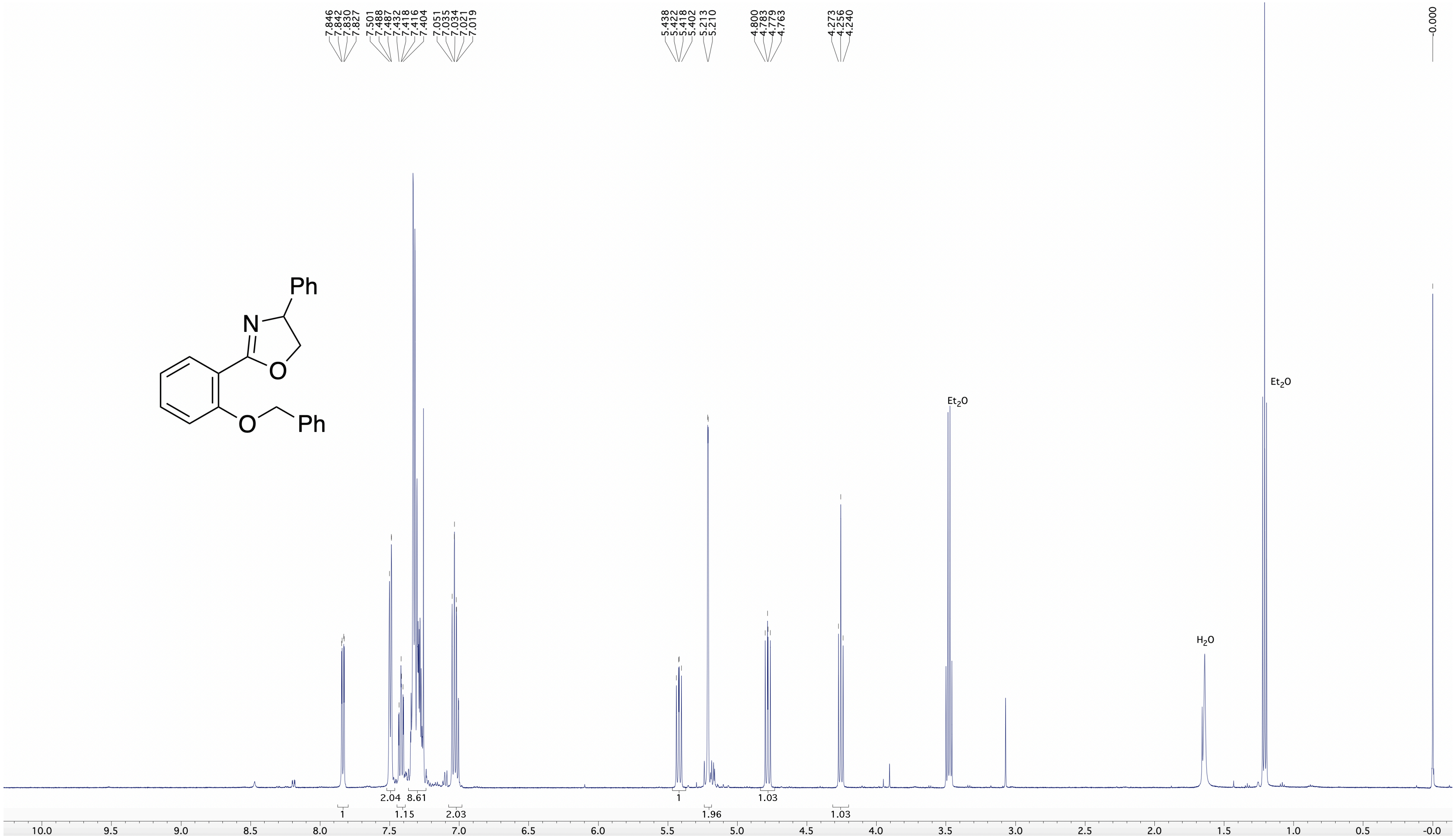

Figure S14. 125 MHz DEPTQ ^{13}C NMR spectrum of **28**

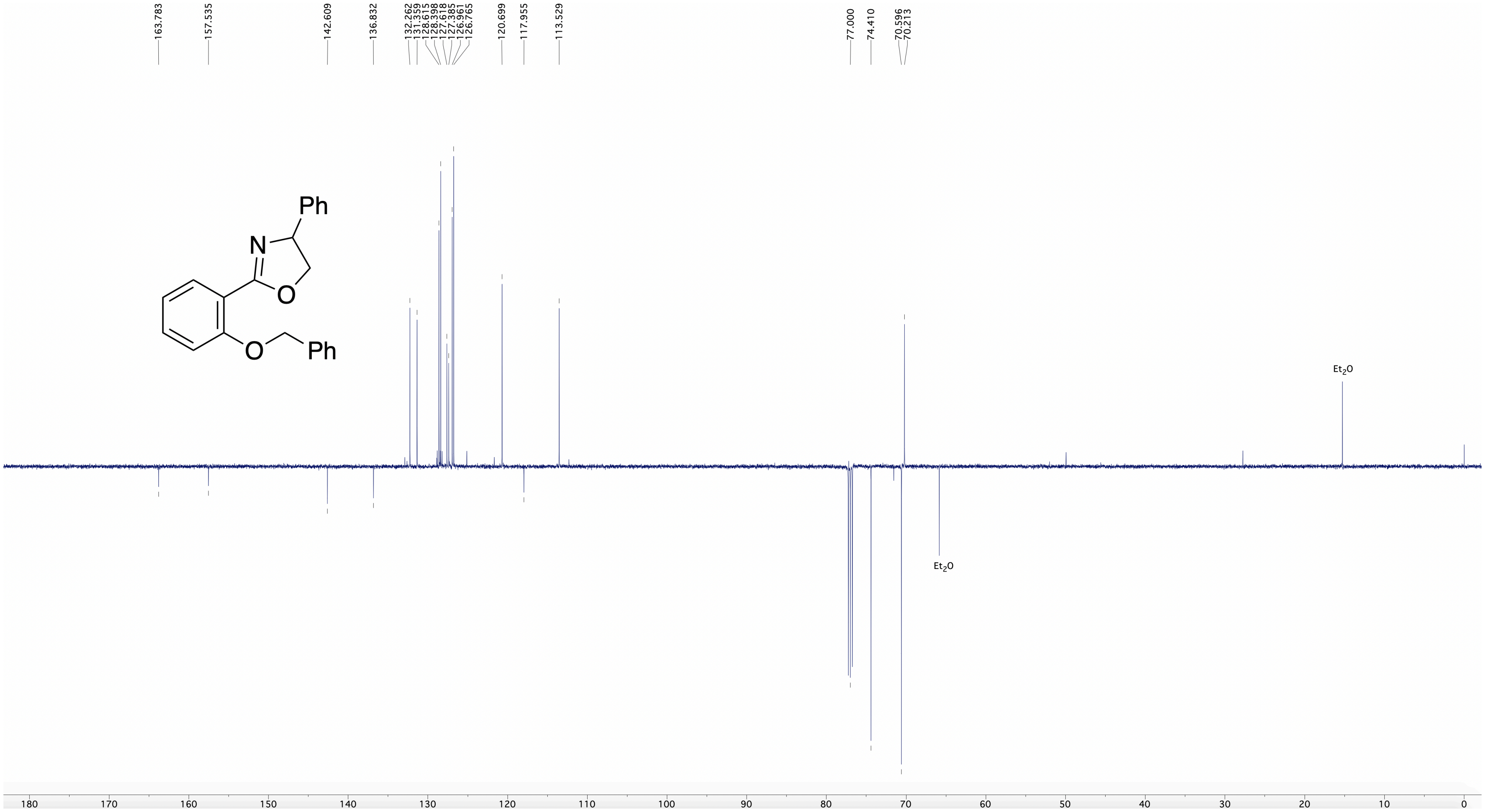

Figure S15. 100 MHz DEPTQ ^{13}C NMR spectrum of **29**

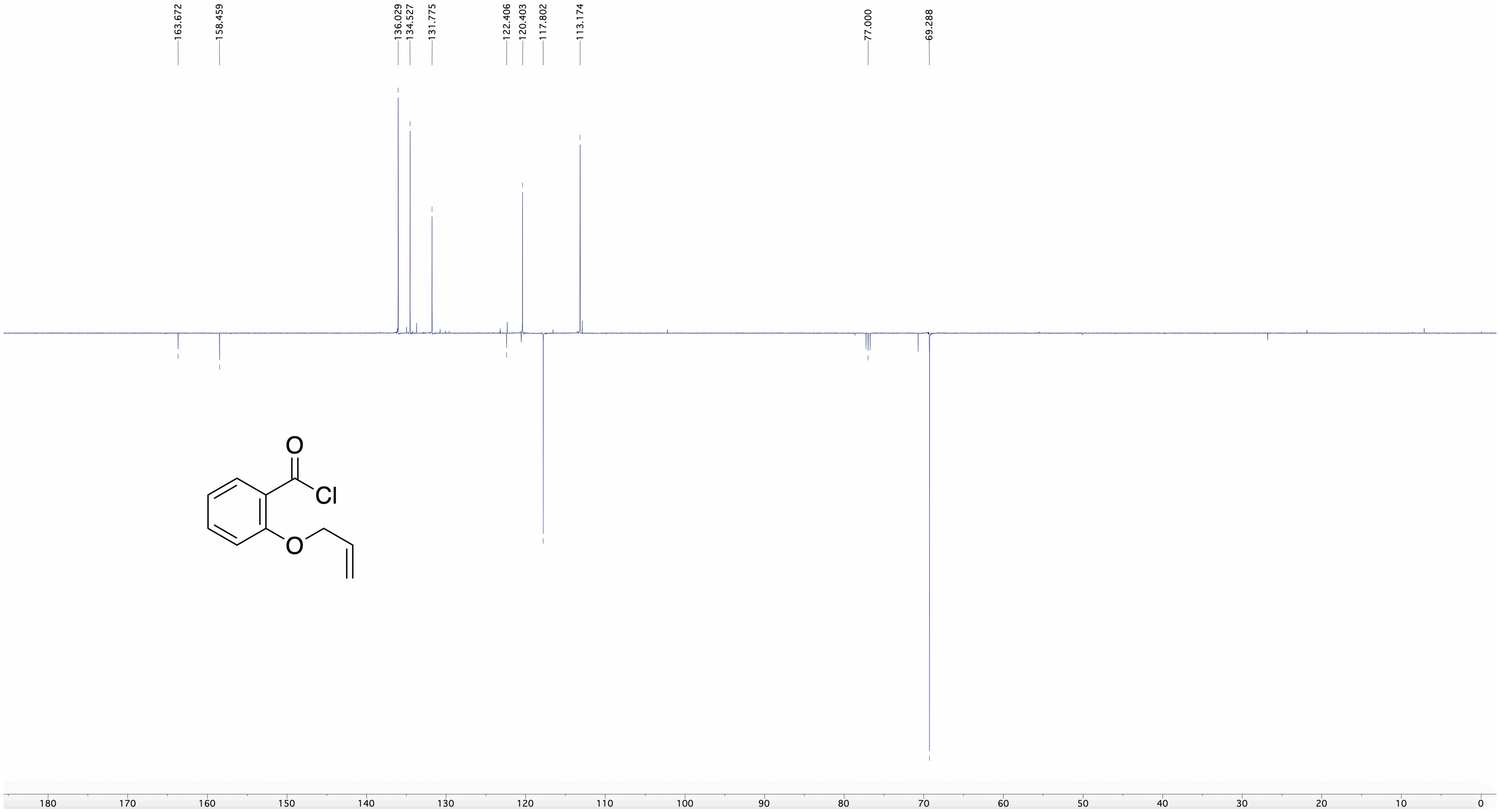

Figure S16. 400 MHz ^1H NMR spectrum of **30**

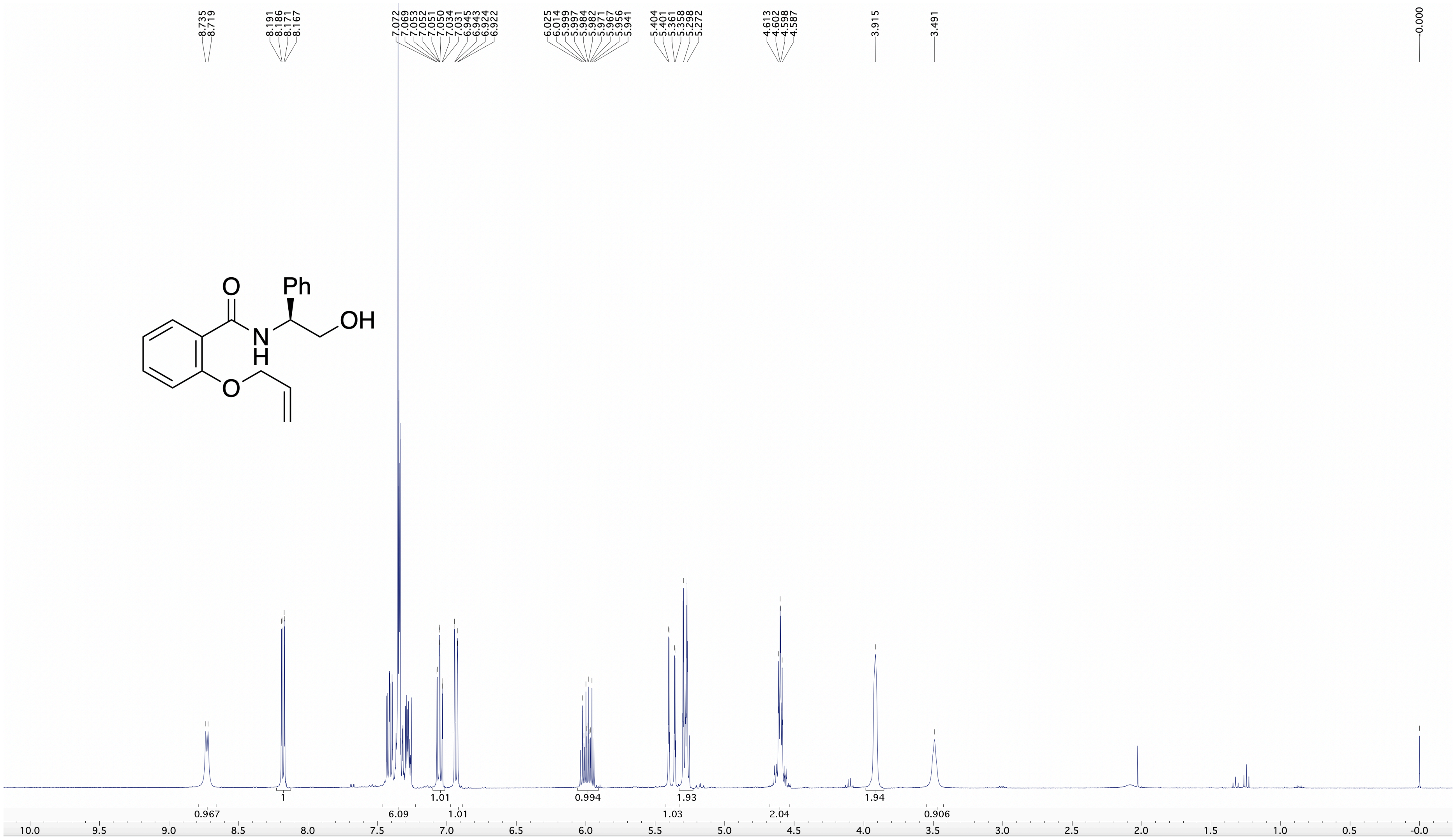

Figure S17. 100 MHz DEPTQ ^{13}C NMR spectrum of **30**

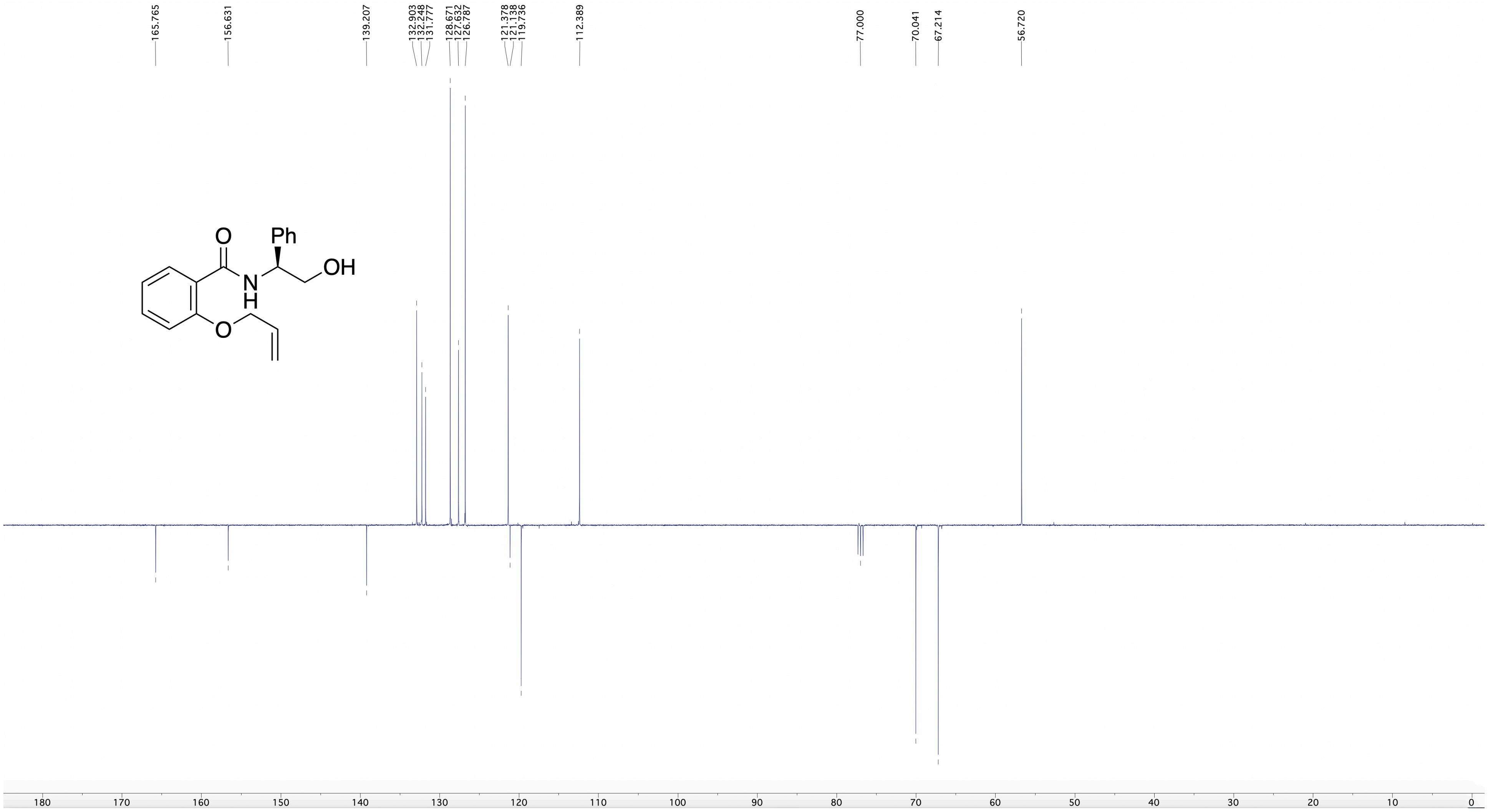

Figure S18. 400 MHz ^{1}H NMR spectrum of **31**

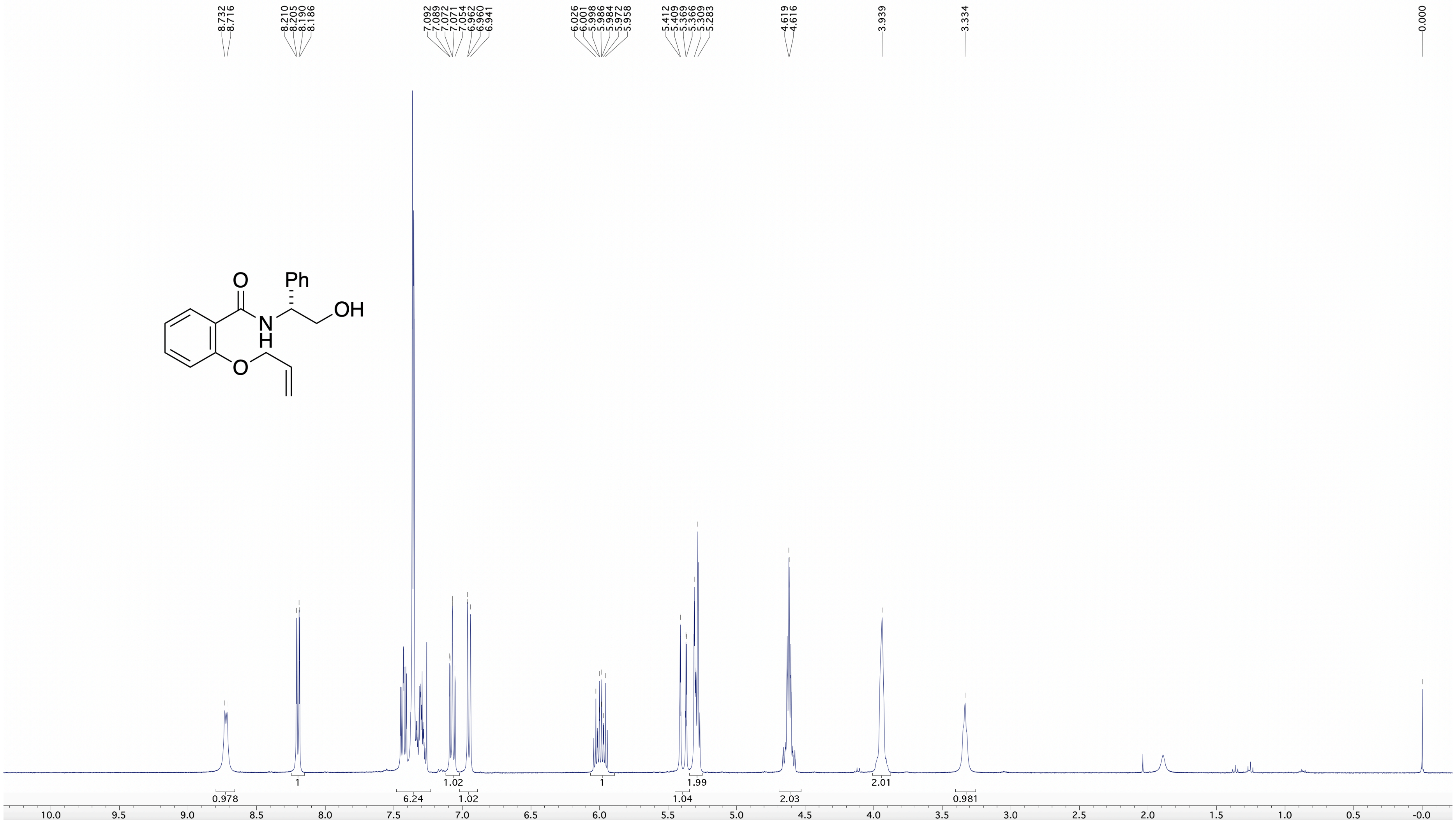

Figure S19. 100 MHz DEPTQ ^{13}C NMR spectrum of **31**

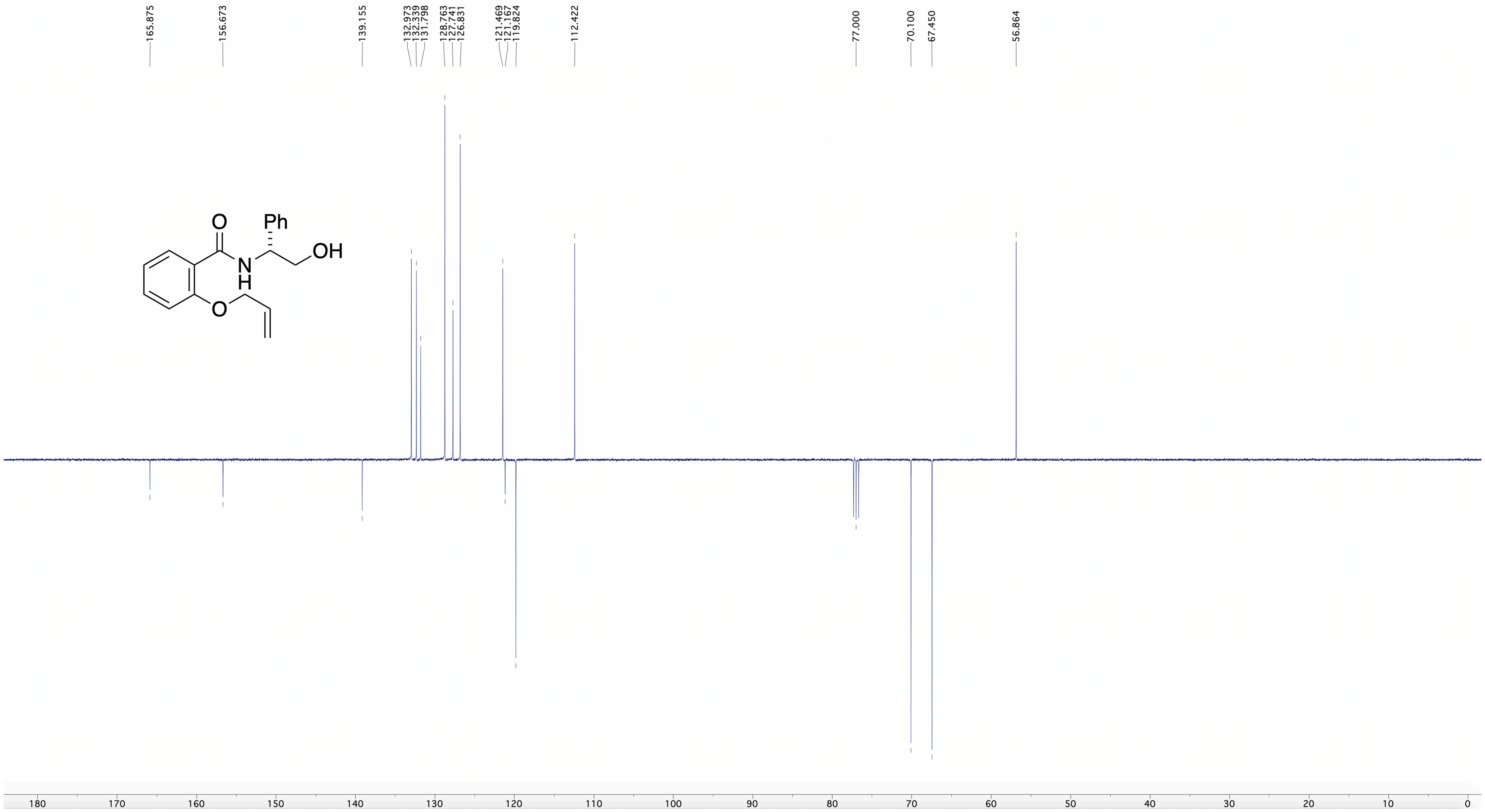

Figure S20. 400 MHz ^{1}H NMR spectrum of **32**

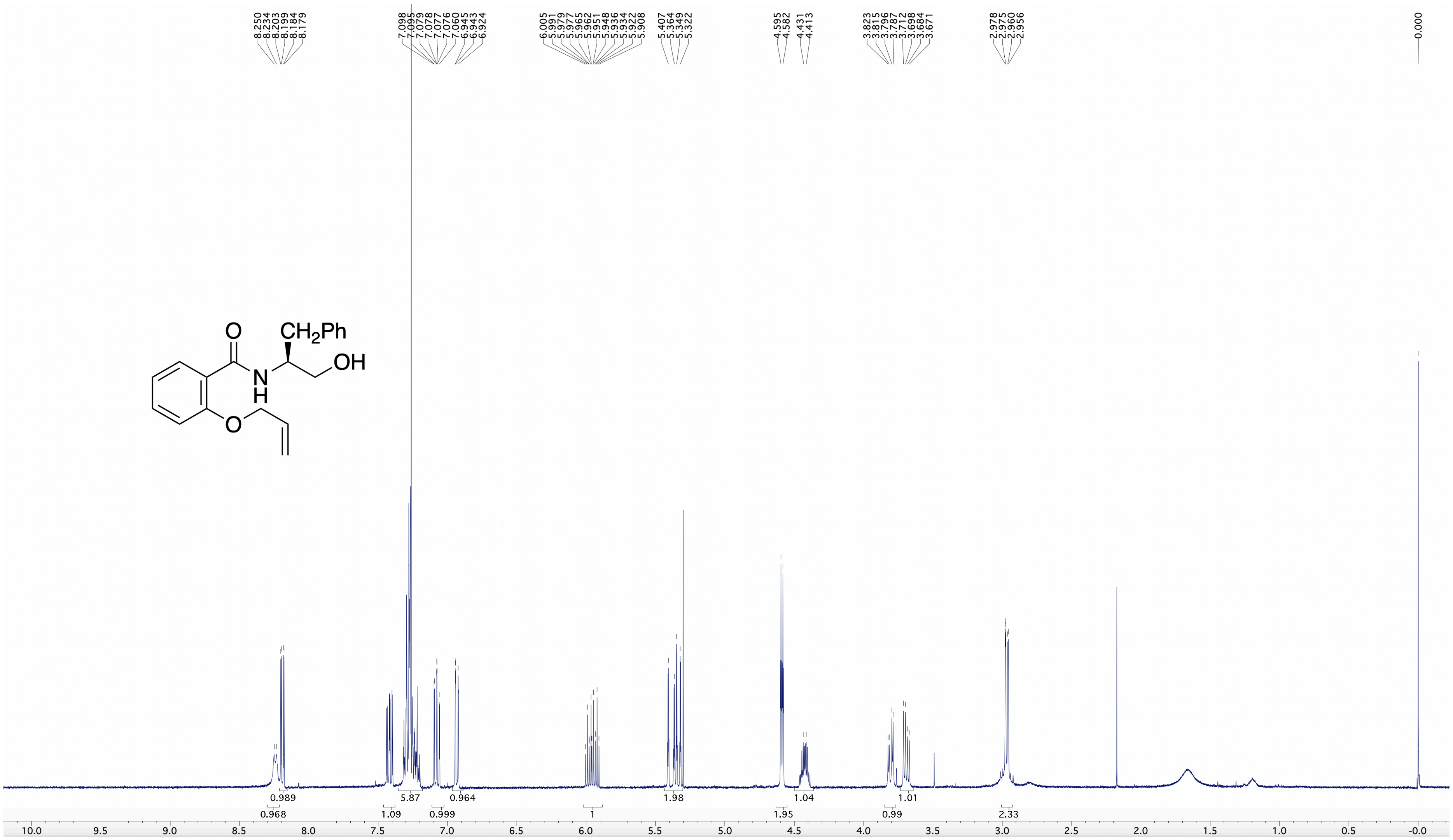

Figure S21. 100 MHz DEPTQ ^{13}C NMR spectrum of **32**

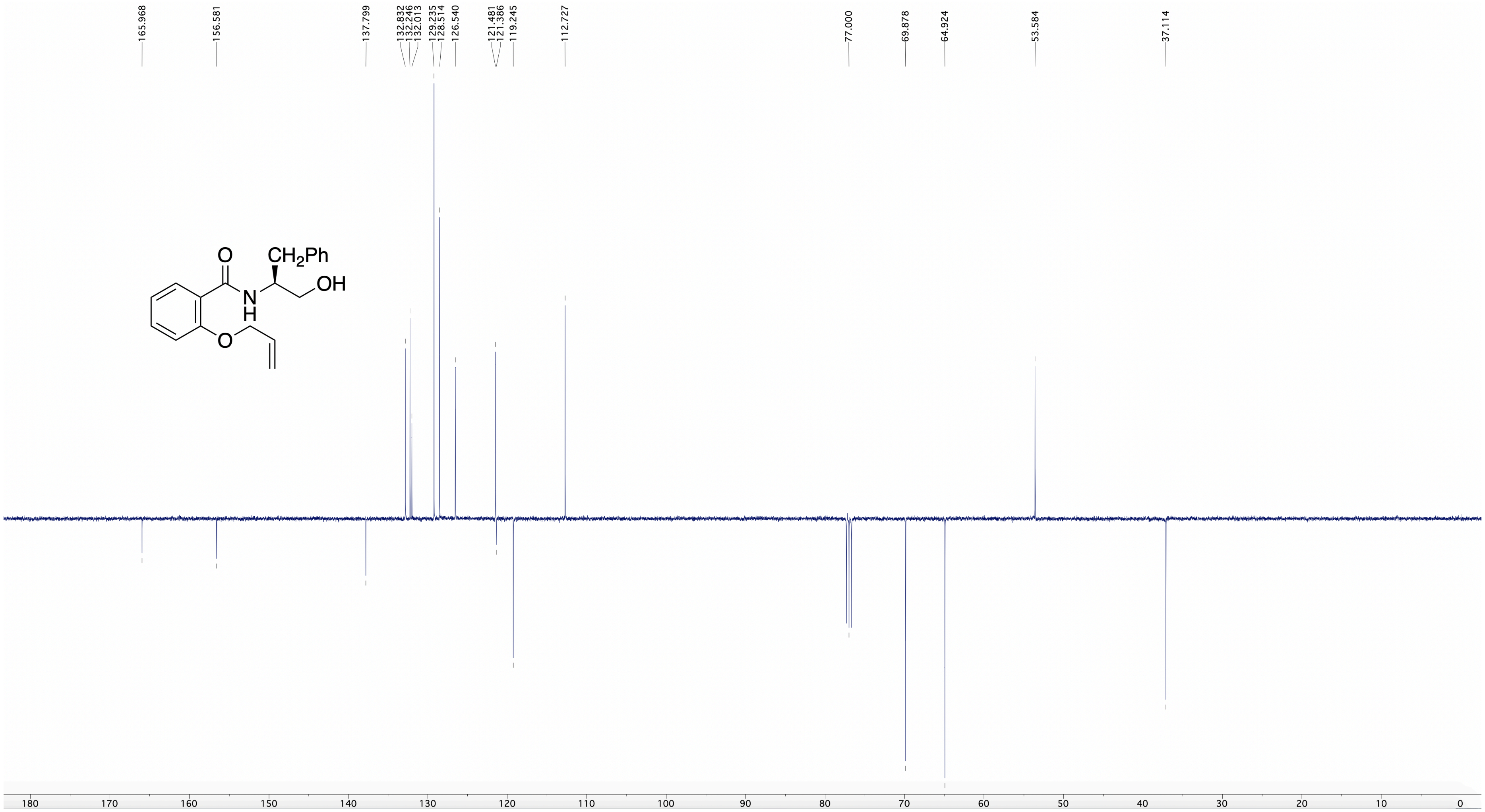

Figure S22. 400 MHz ^{1}H NMR spectrum of **33**

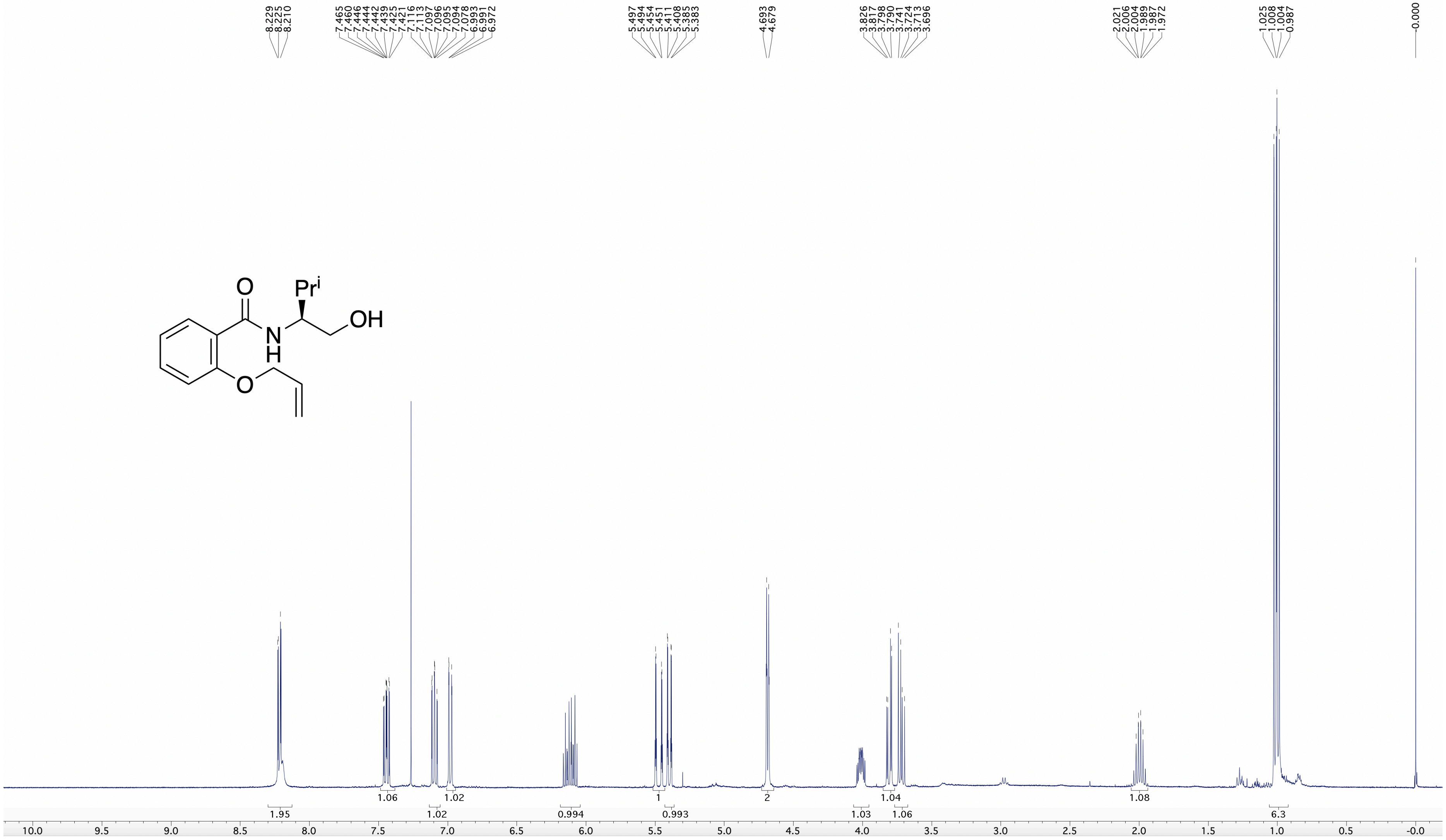

Figure S23. 100 MHz DEPTQ ^{13}C NMR spectrum of **33**

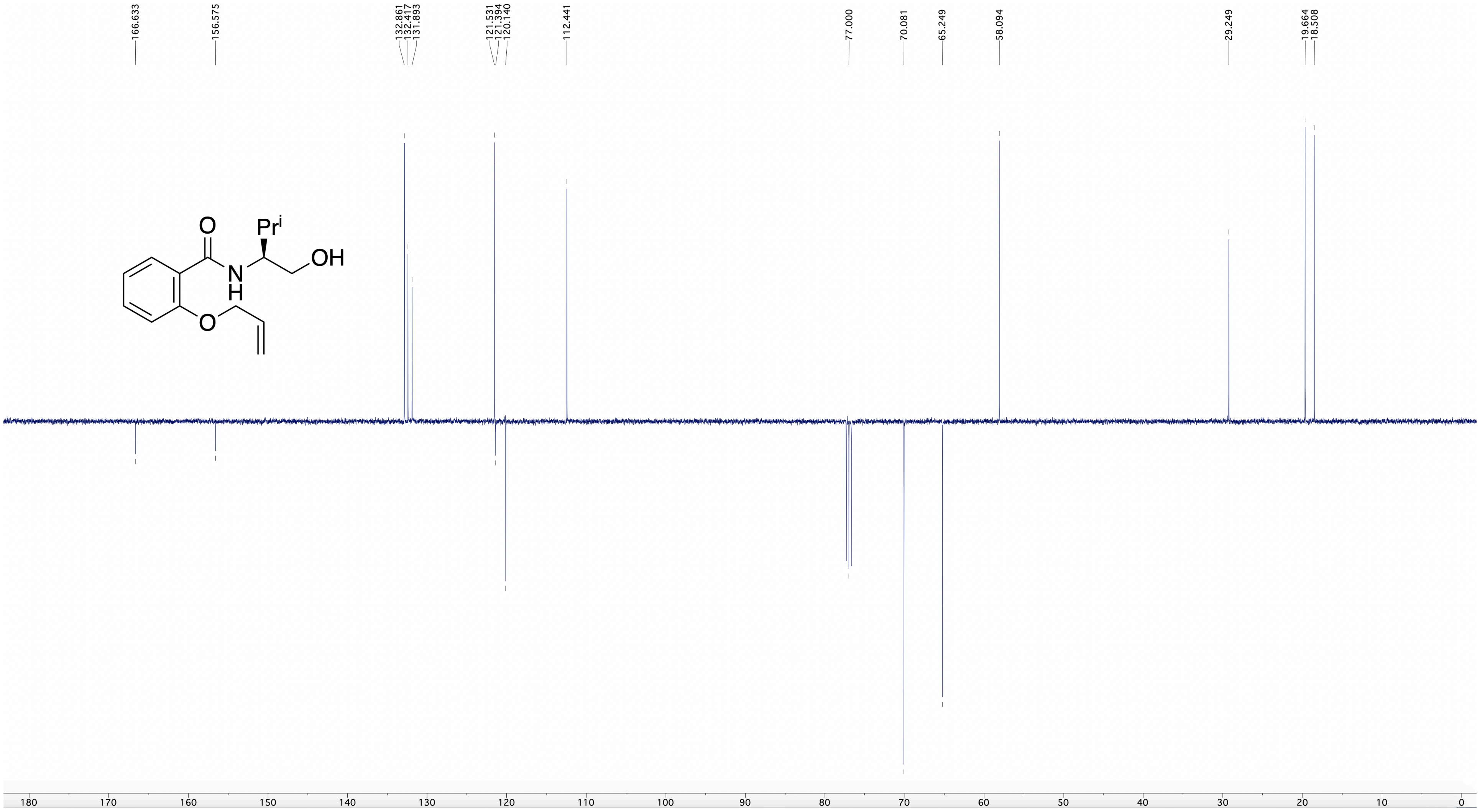

Figure S24. 400 MHz ^{1}H NMR spectrum of **34**

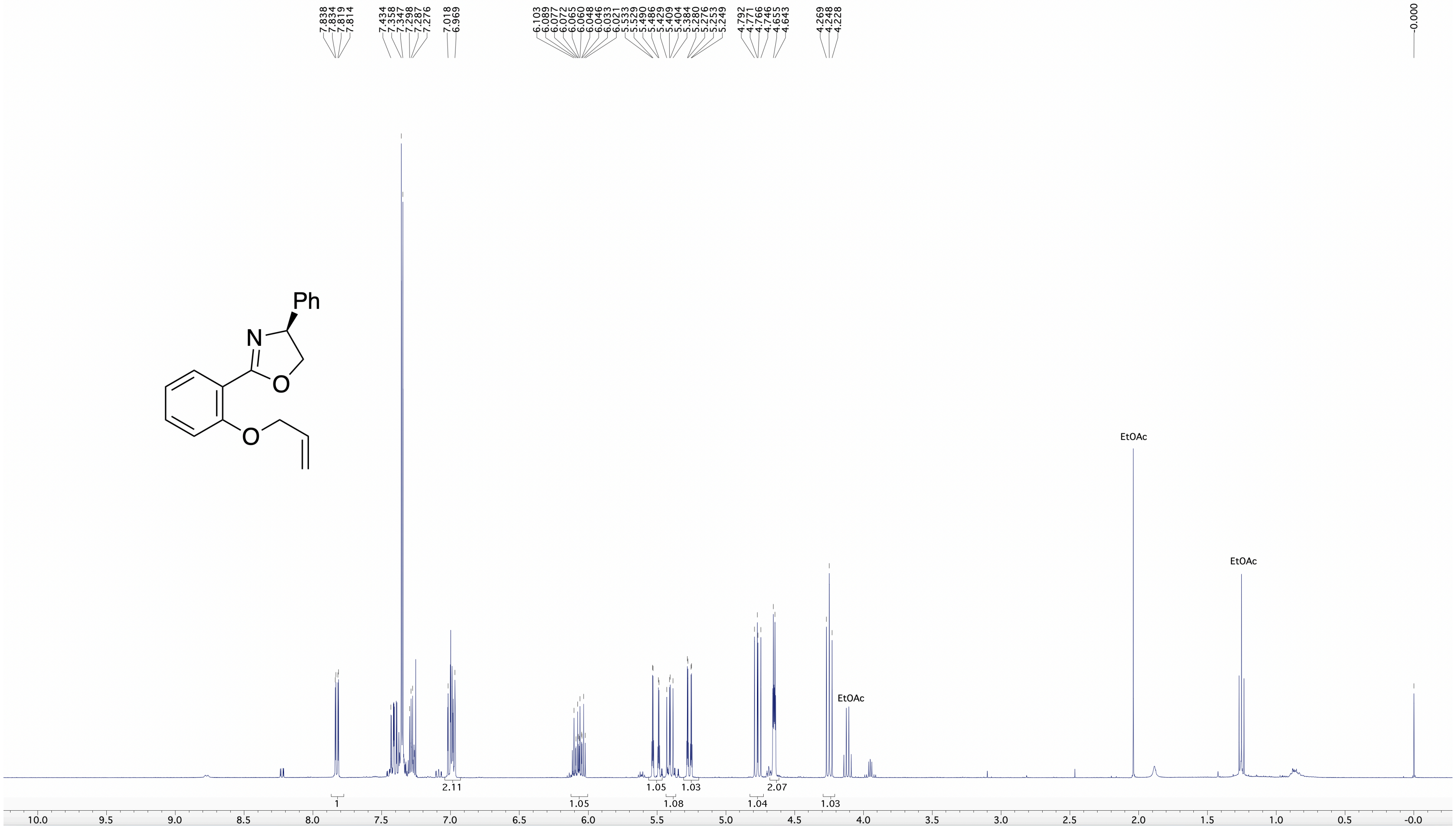

Figure S25. 100 MHz DEPTQ ^{13}C NMR spectrum of **34**

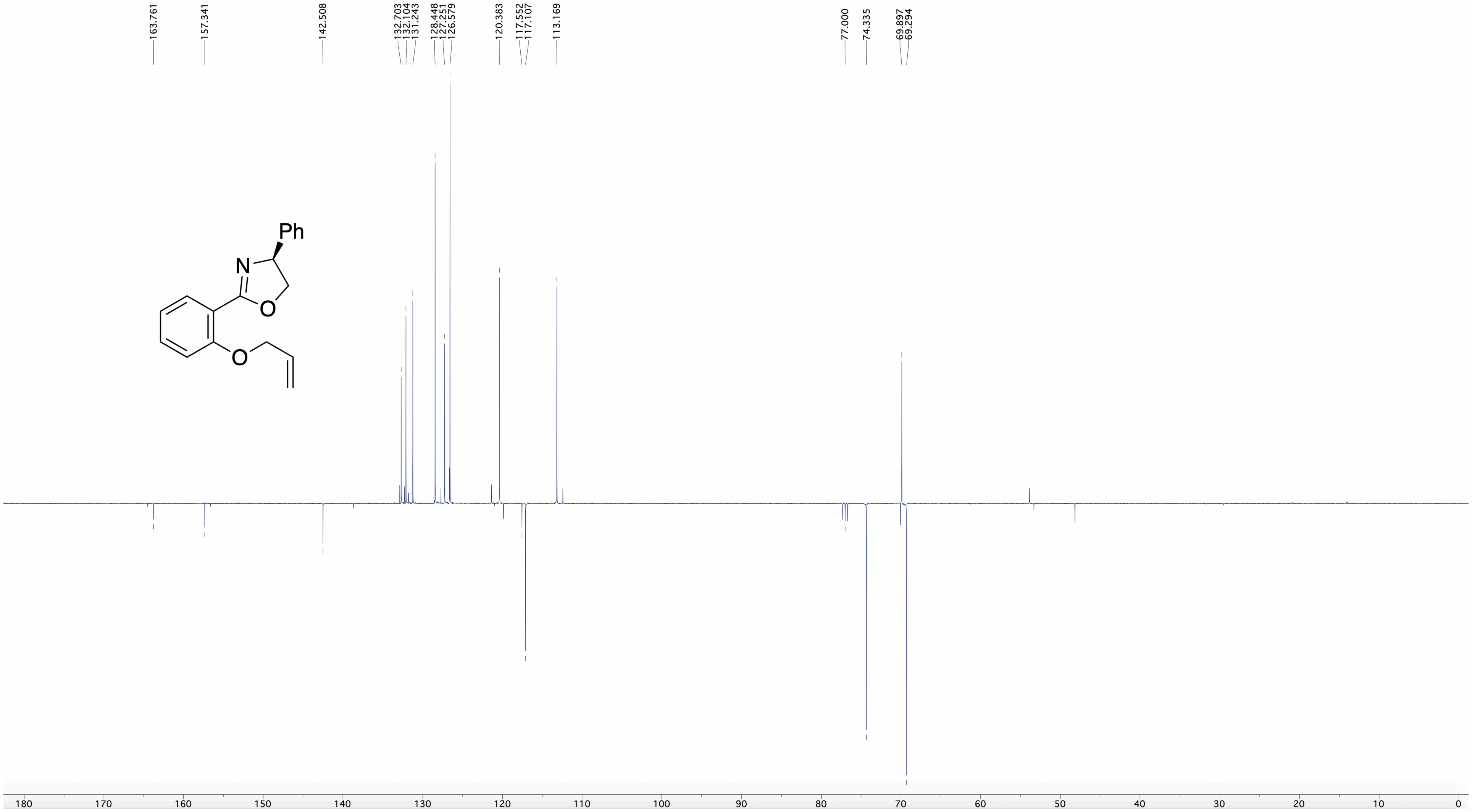

Figure S26. 400 MHz ^{1}H NMR spectrum of **35**

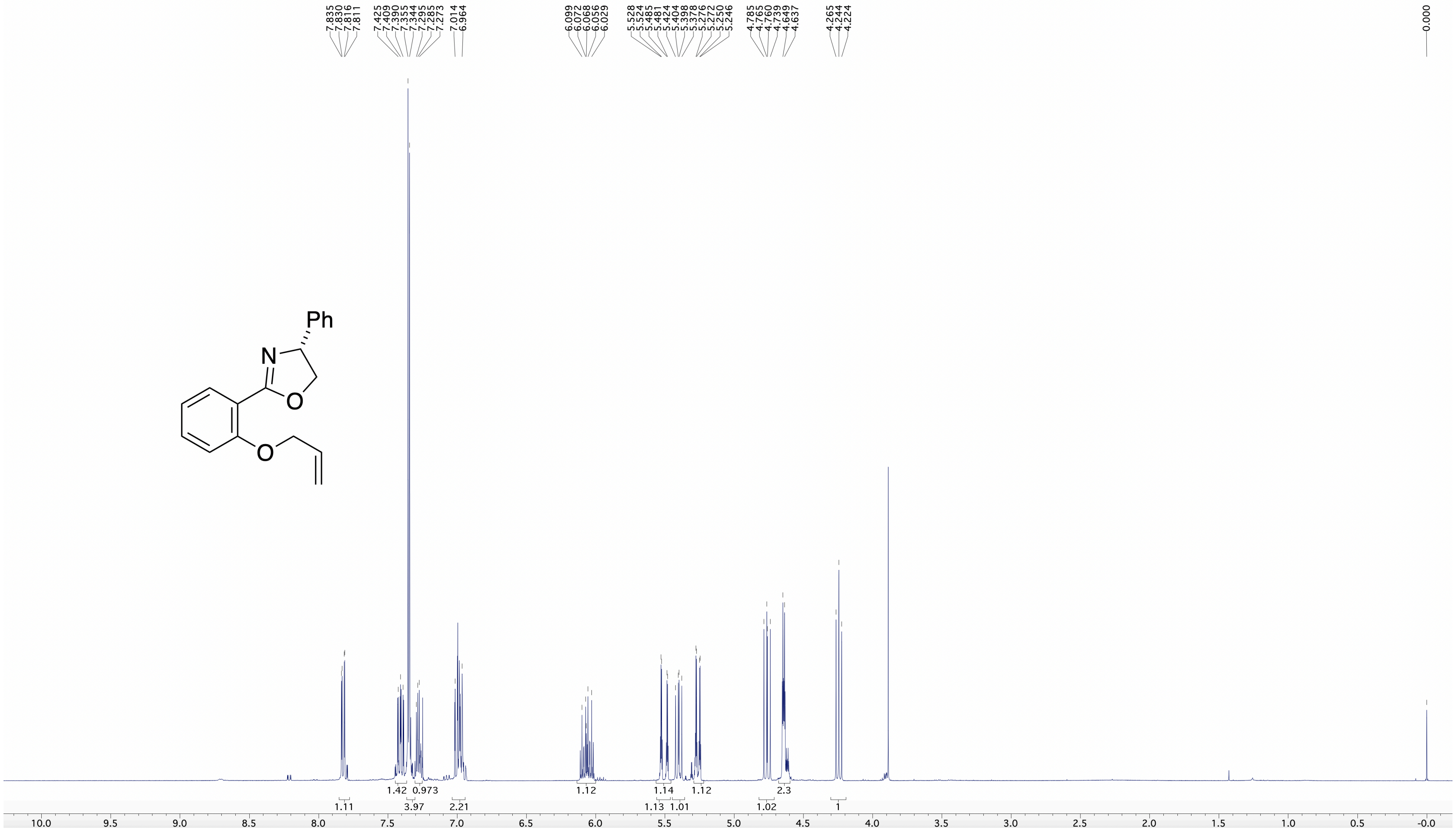

Figure S27. 100 MHz DEPTQ ^{13}C NMR spectrum of **35**

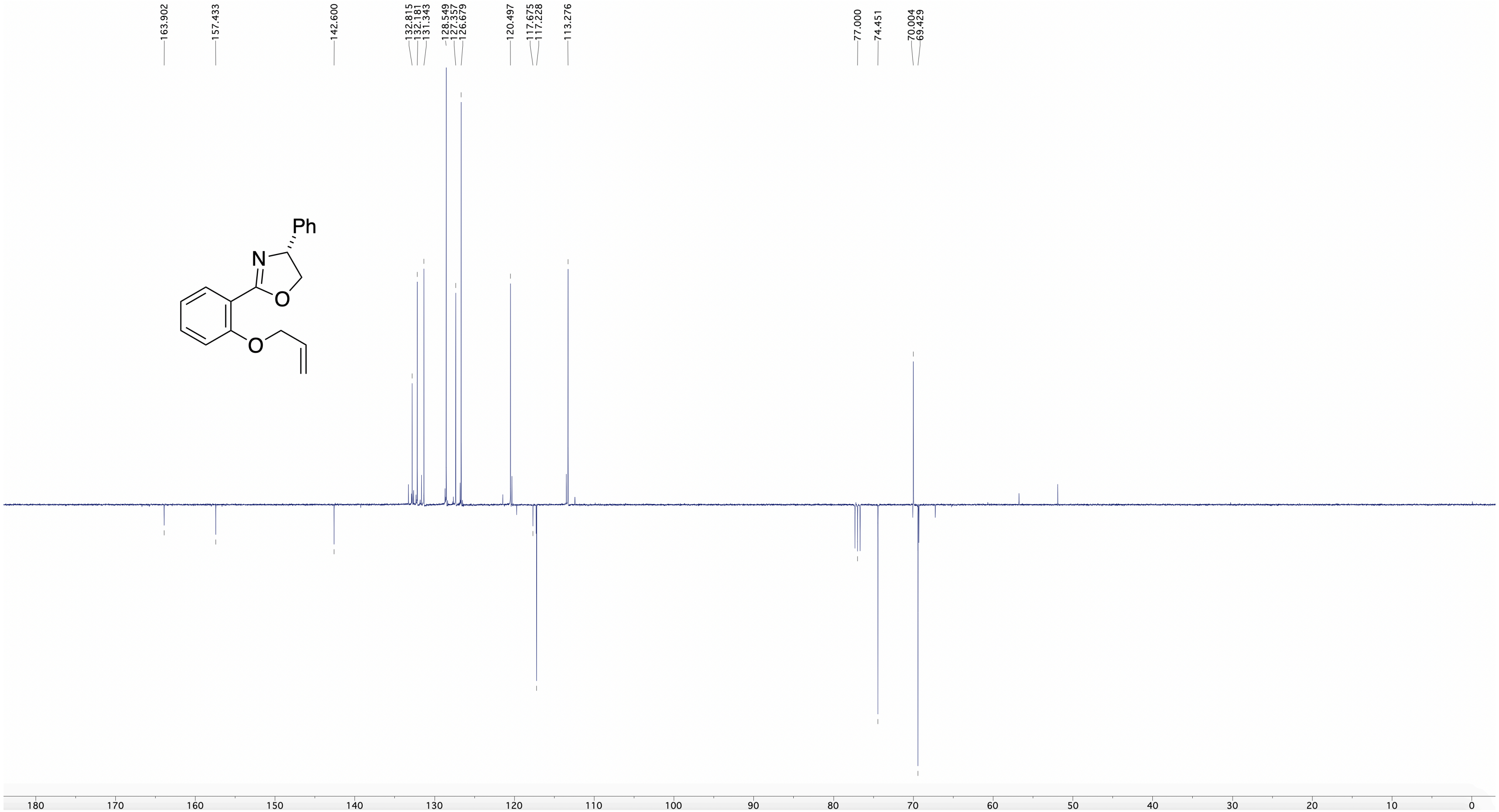

Figure S28. 400 MHz ^{1}H NMR spectrum of **36**

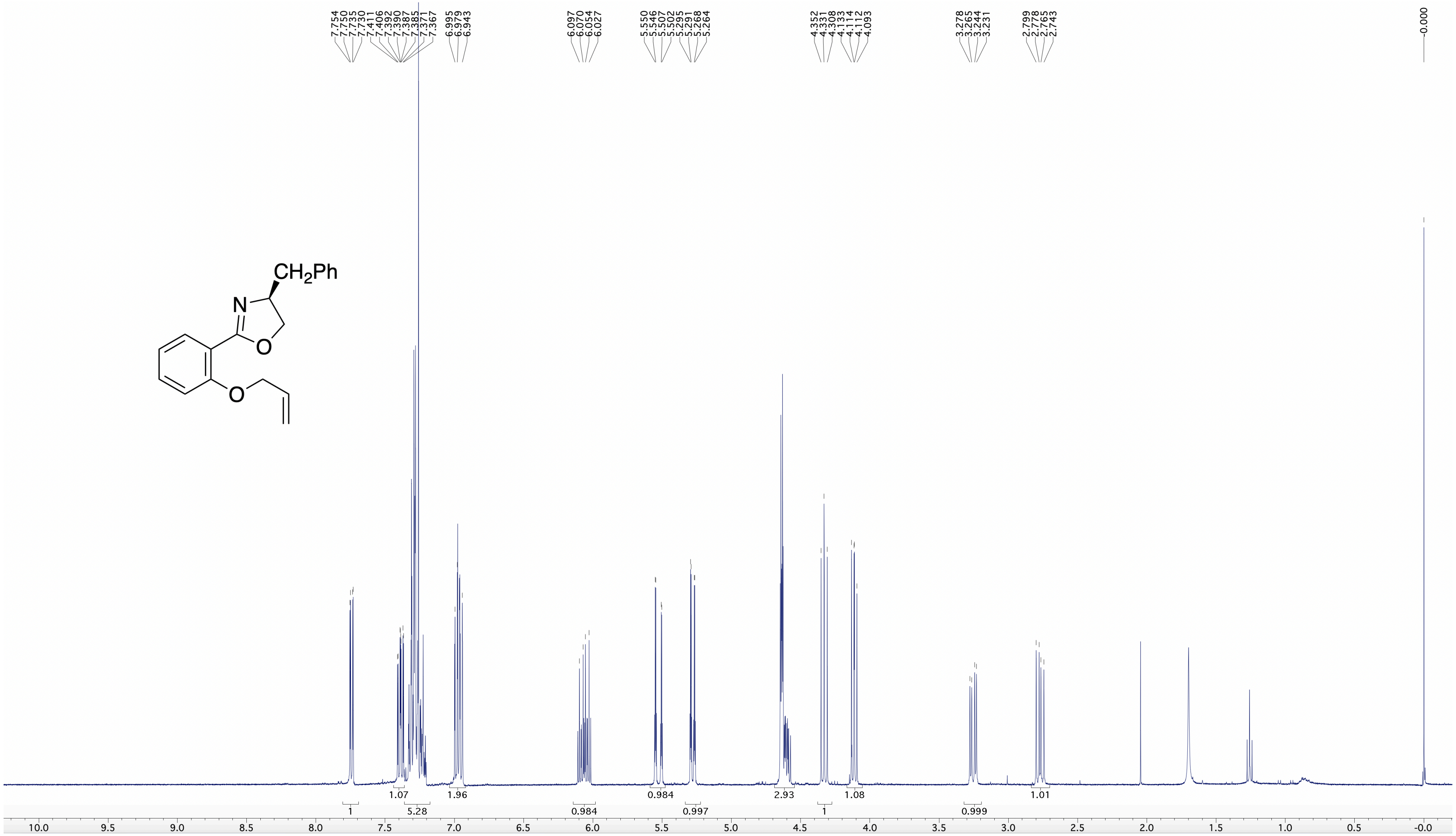

Figure S29. 100 MHz DEPTQ ^{13}C NMR spectrum of **36**

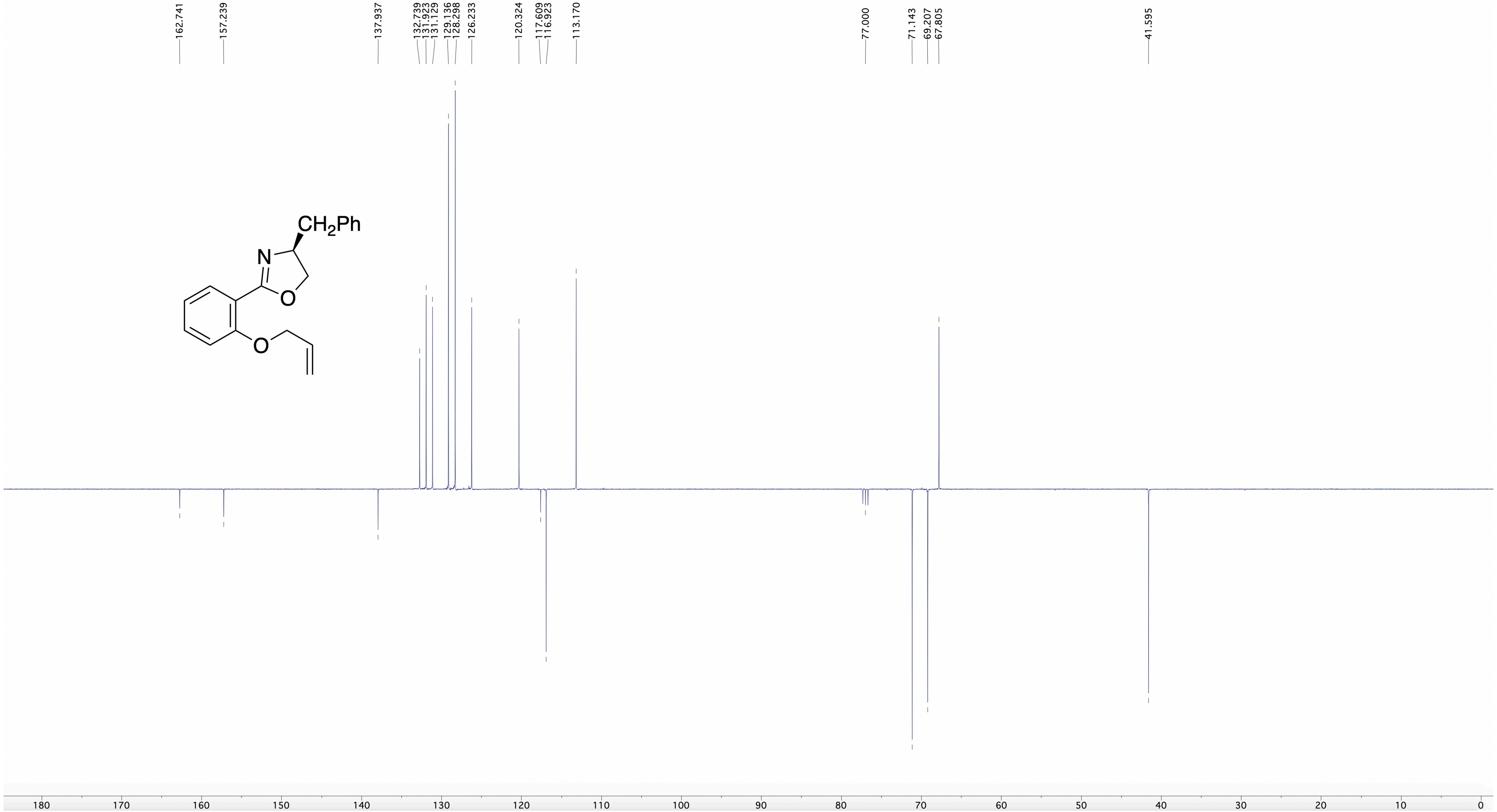

Figure S30. 400 MHz ^{1}H NMR spectrum of **37**

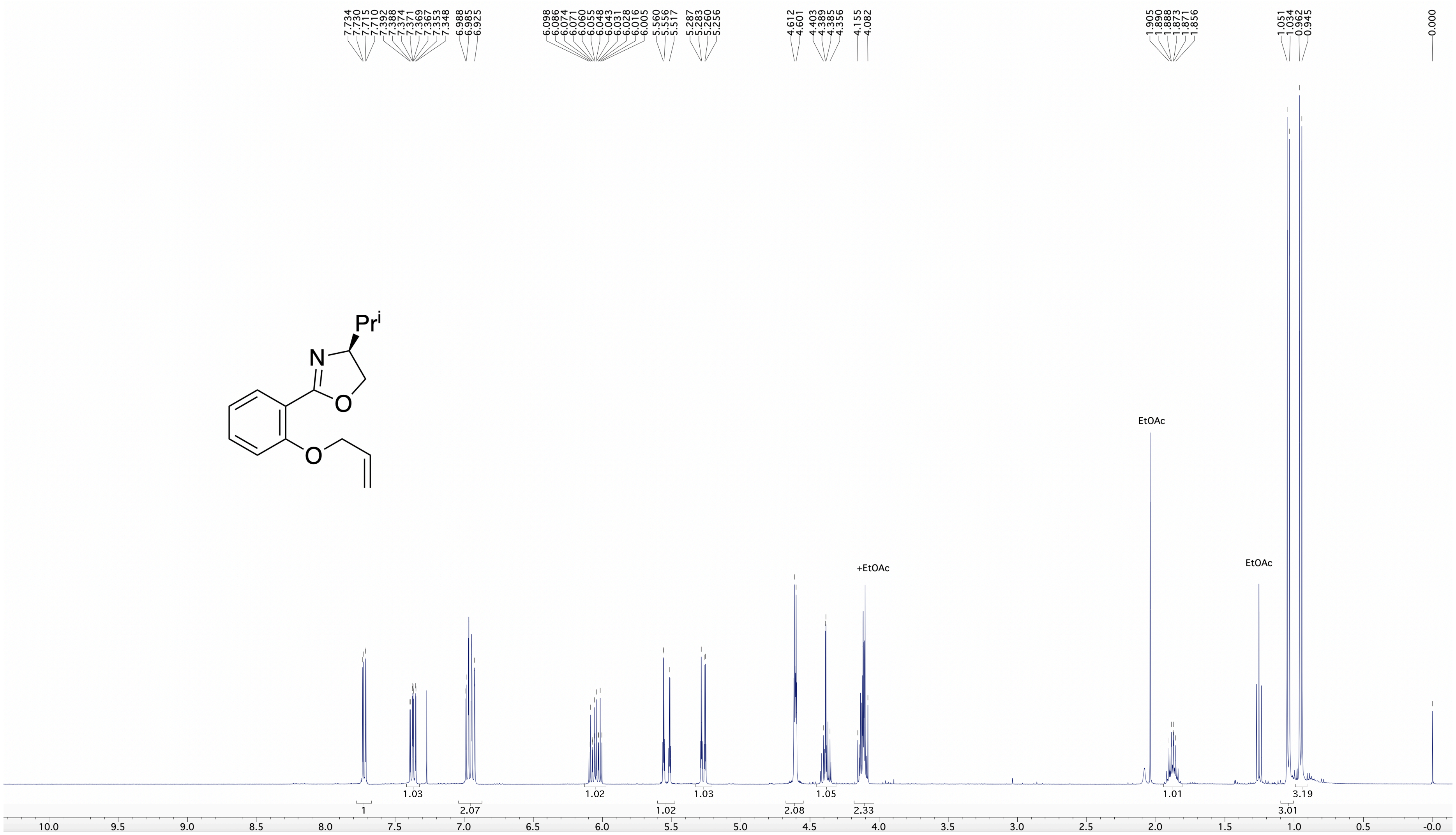

Figure S31. 100 MHz DEPTQ ^{13}C NMR spectrum of **37**

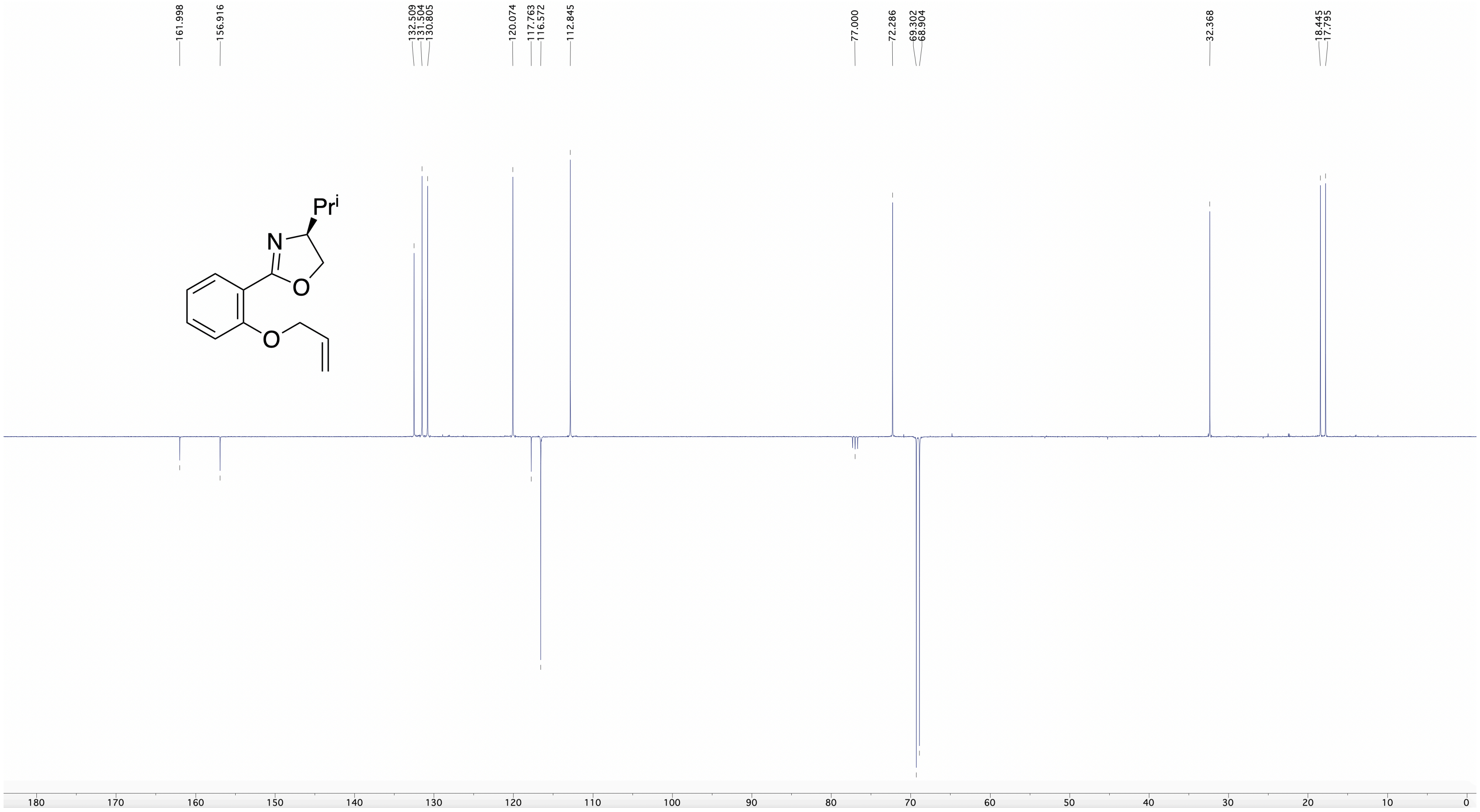

Figure S32. 400 MHz ^{1}H NMR spectrum of **42**

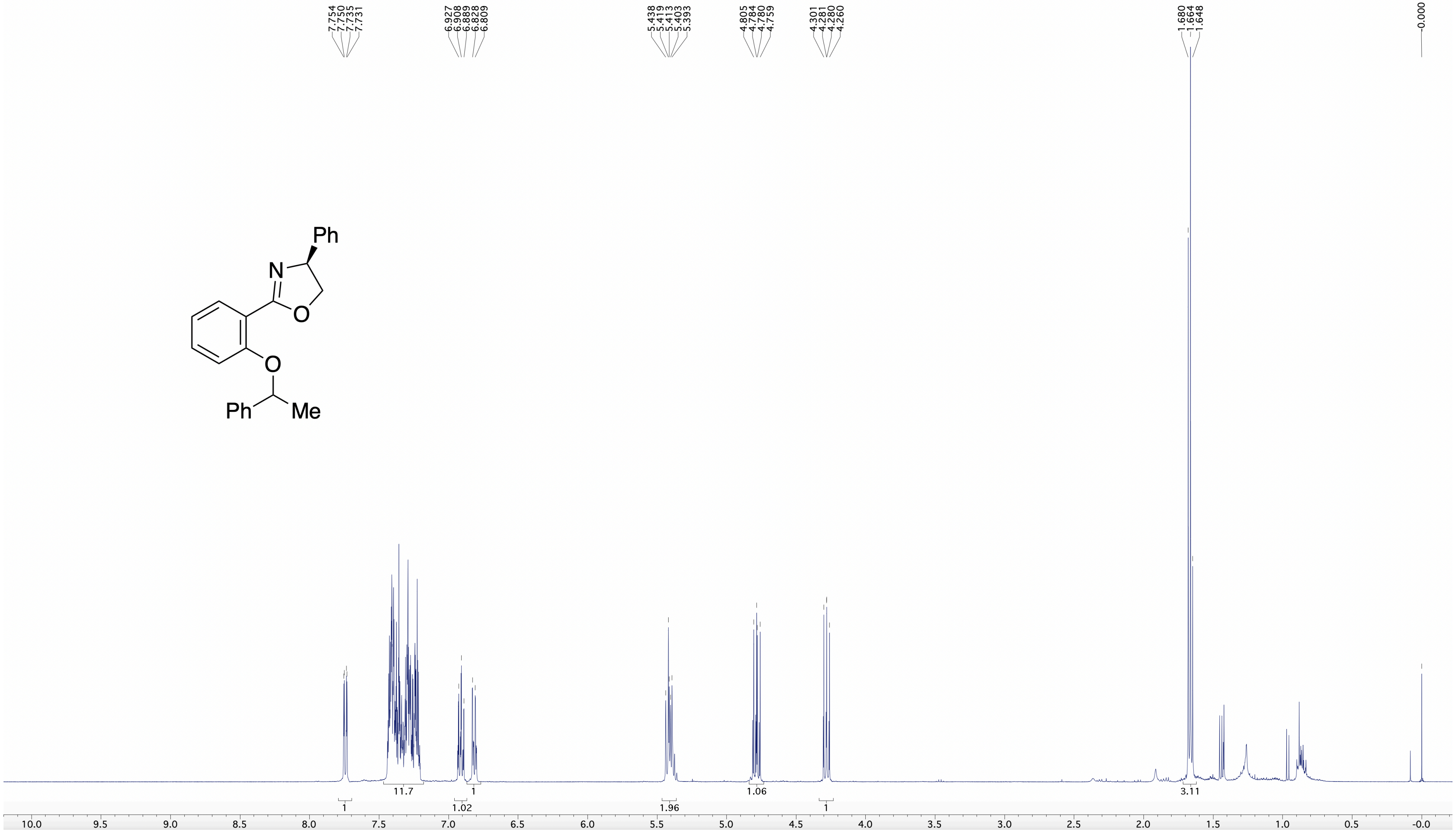

Figure S33. 100 MHz DEPTQ ^{13}C NMR spectrum of **42**

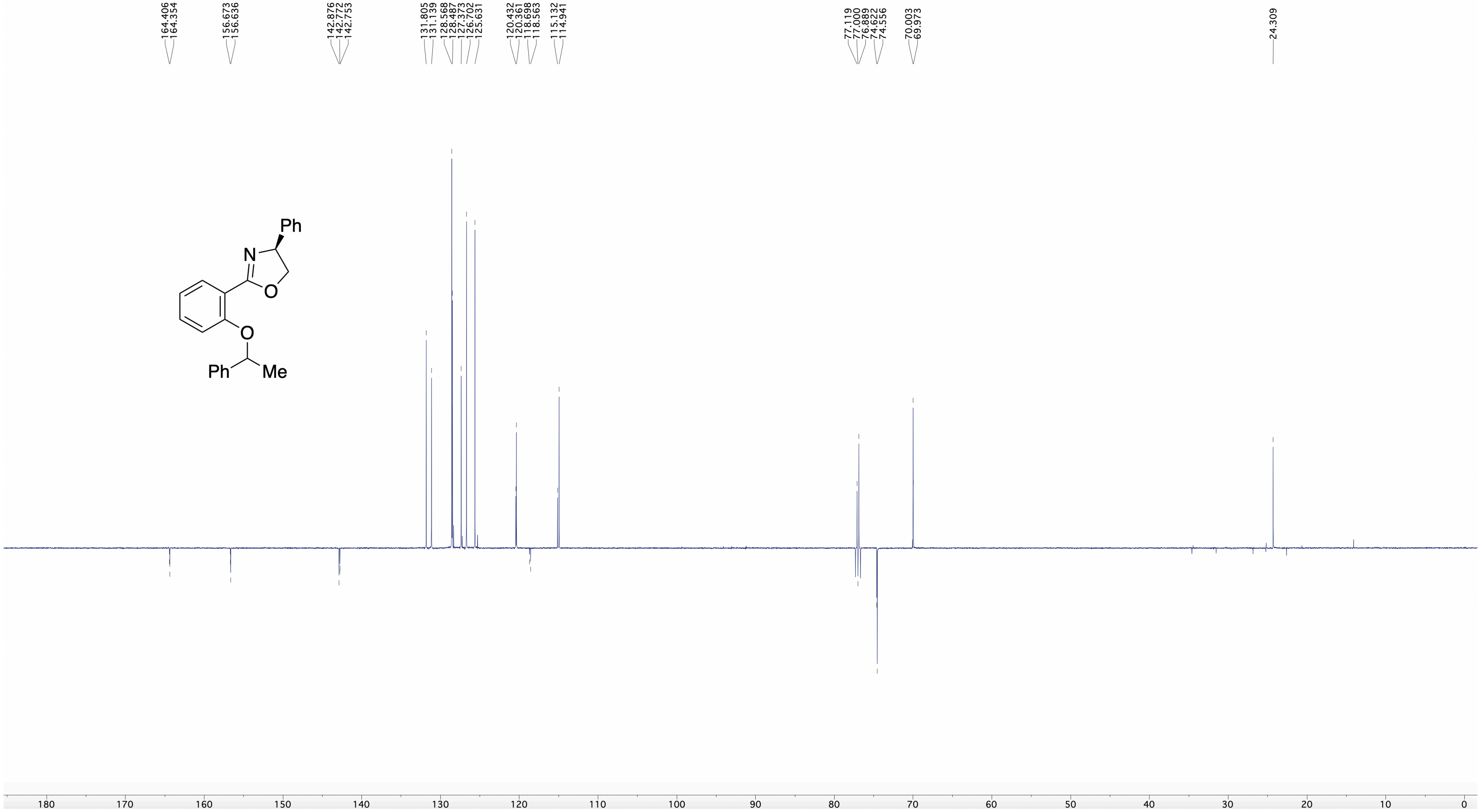

Figure S34. 400 MHz ^{1}H NMR spectrum of **43**

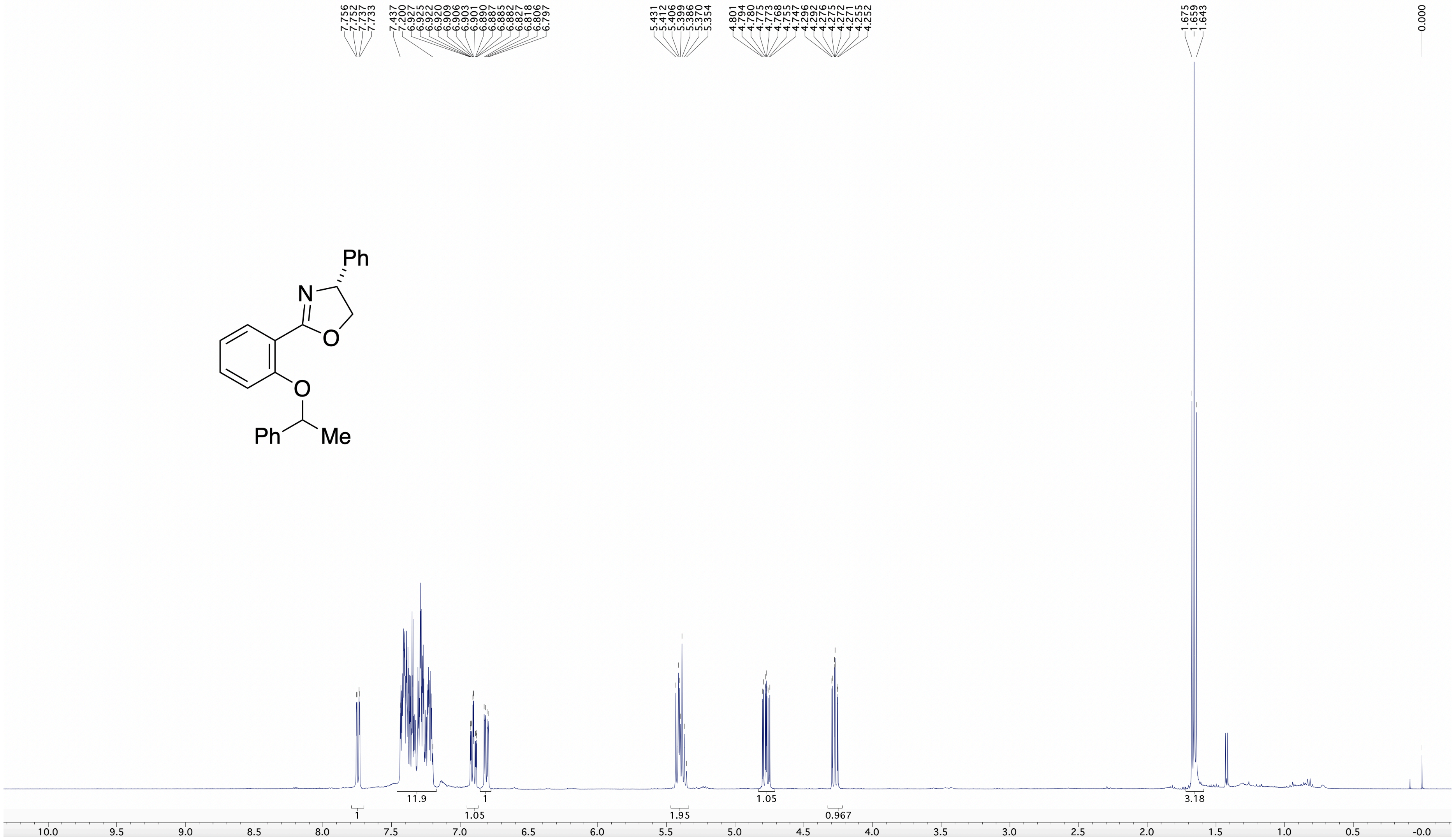

Figure S35. 100 MHz DEPTQ ^{13}C NMR spectrum of **43**

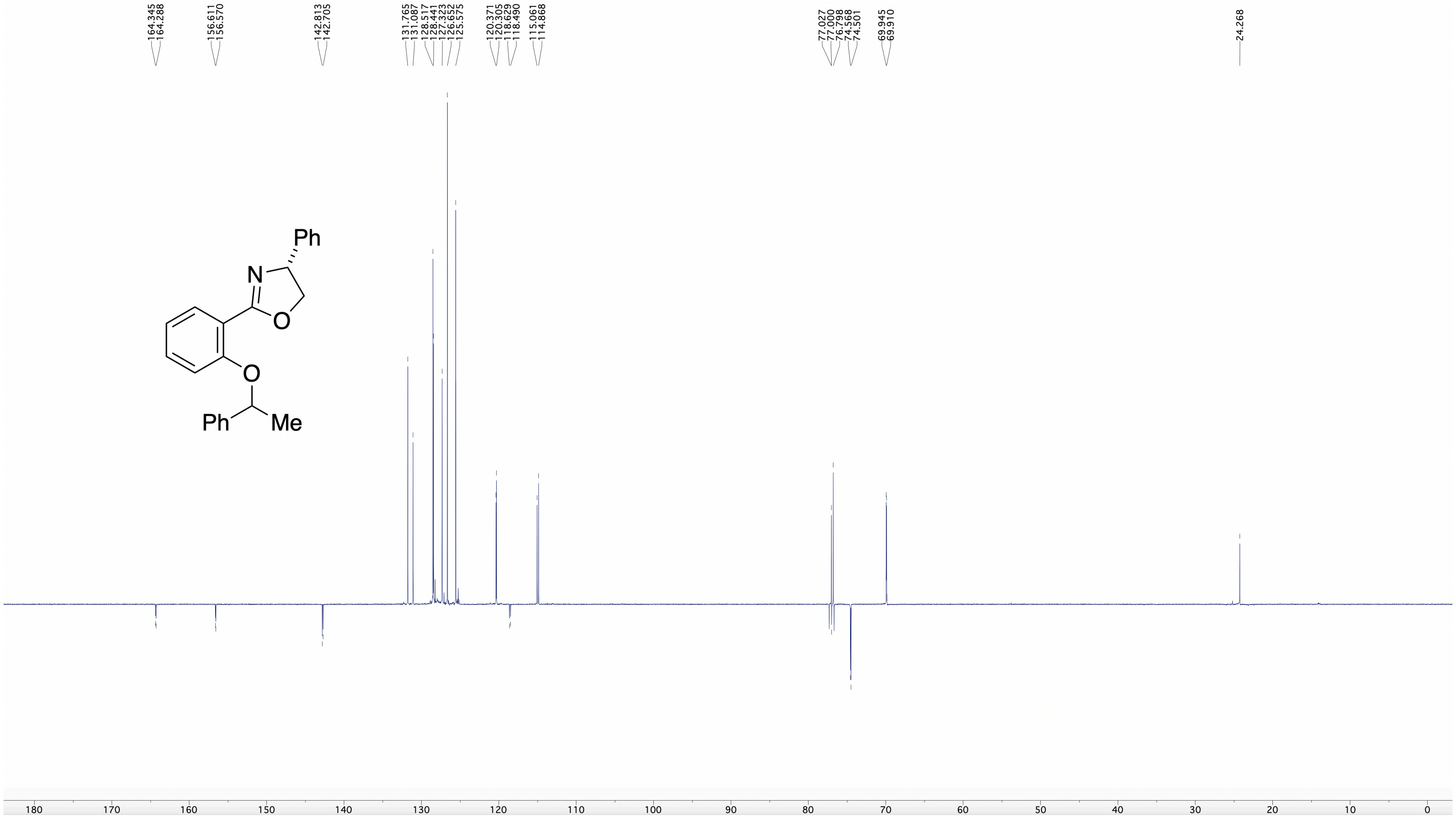

Figure S36. 400 MHz ^{1}H NMR spectrum of **44**

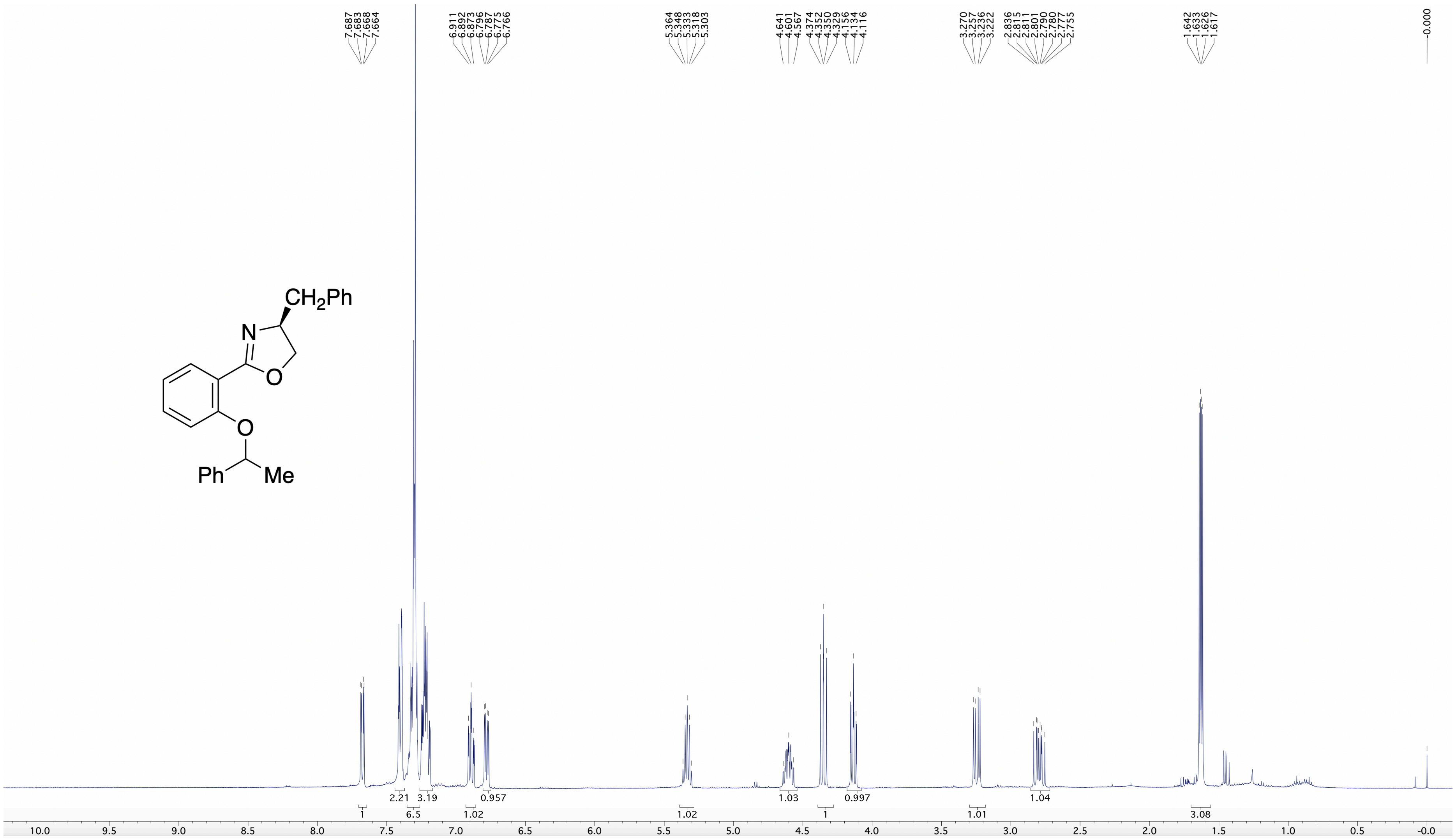

Figure S37. 10 MHz DEPTQ ^{13}C NMR spectrum of **44**

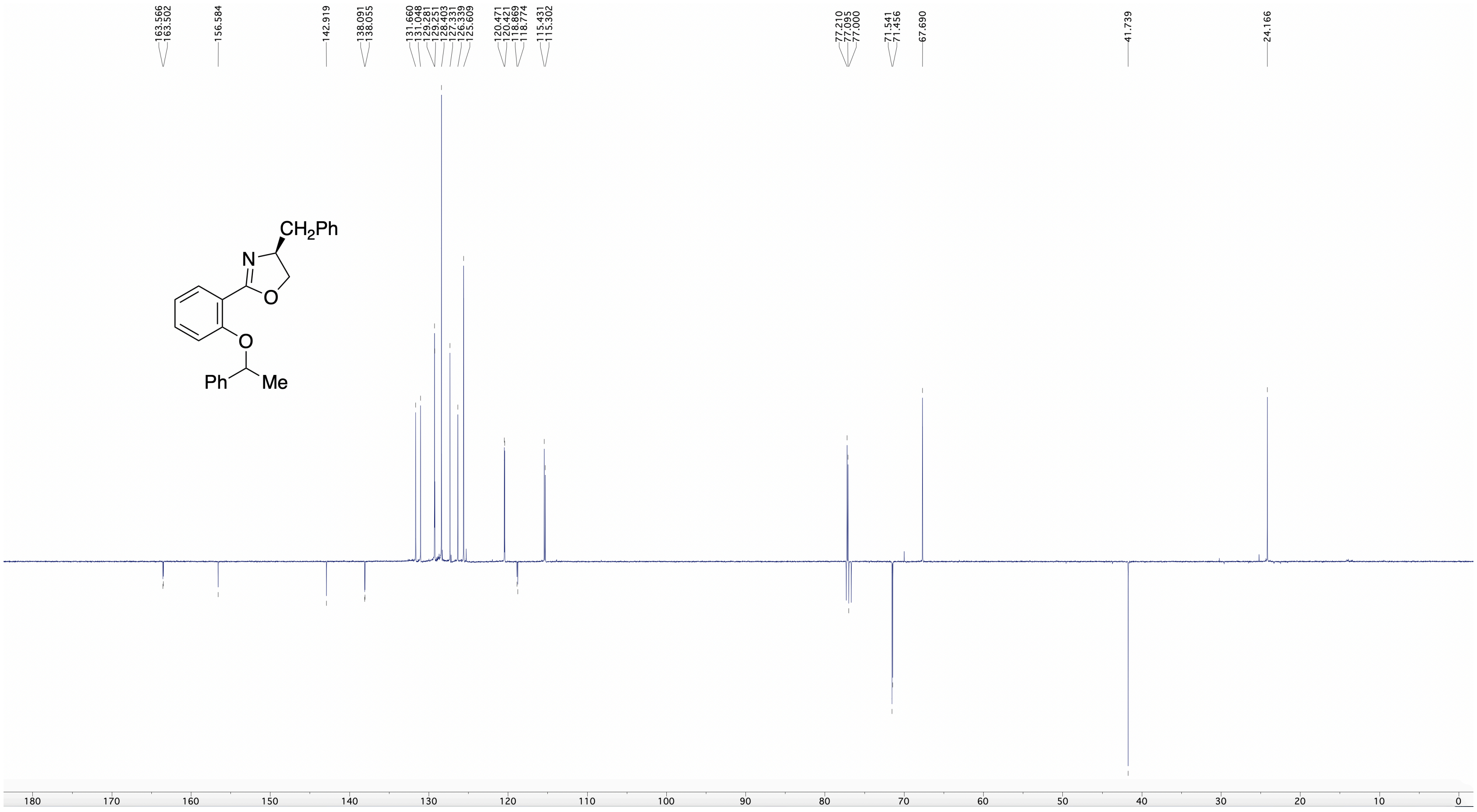

Figure S38. 400 MHz ^{1}H NMR spectrum of **45**

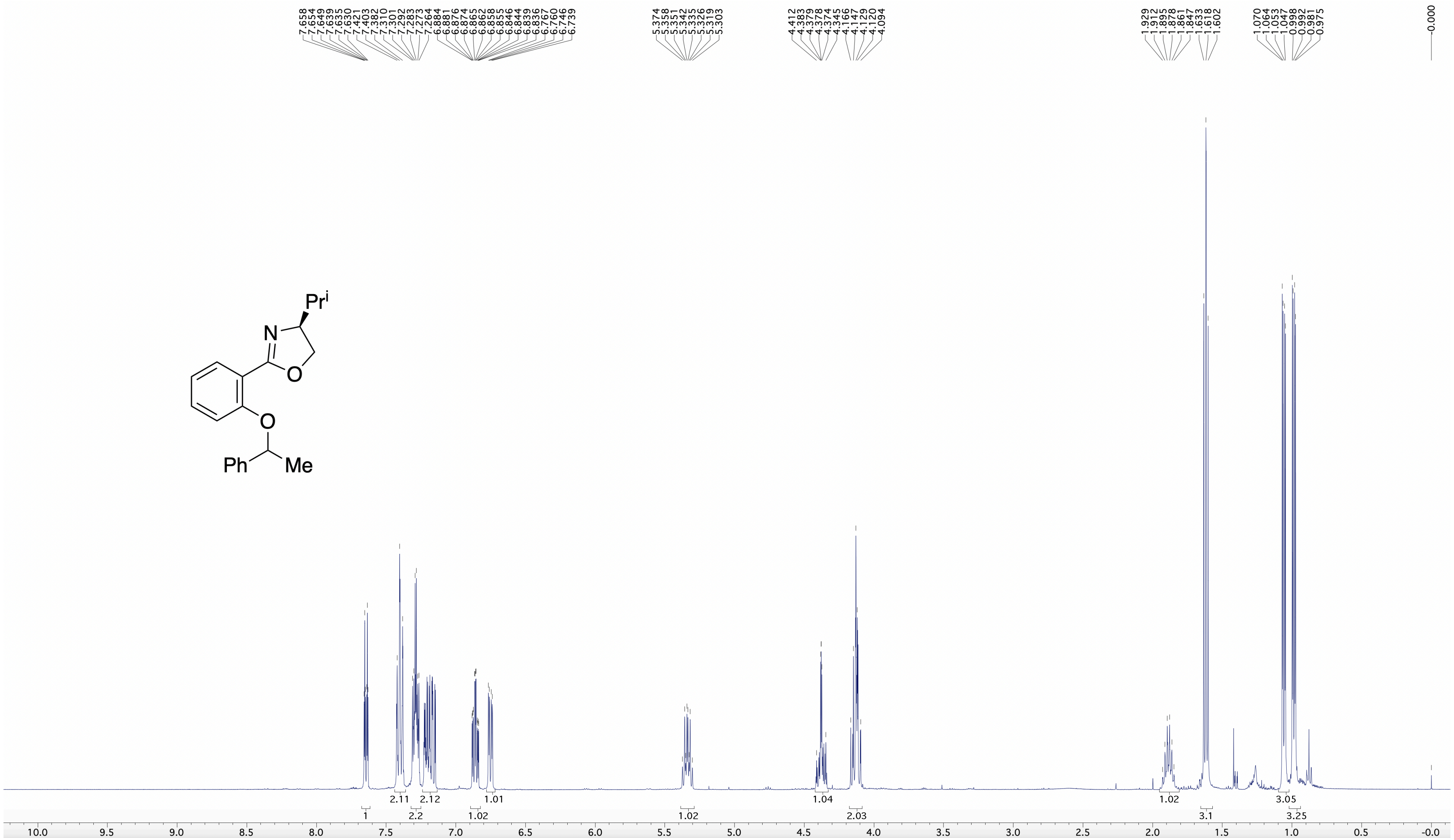

Figure S39. 100 MHz DEPTQ ^{13}C NMR spectrum of **45**

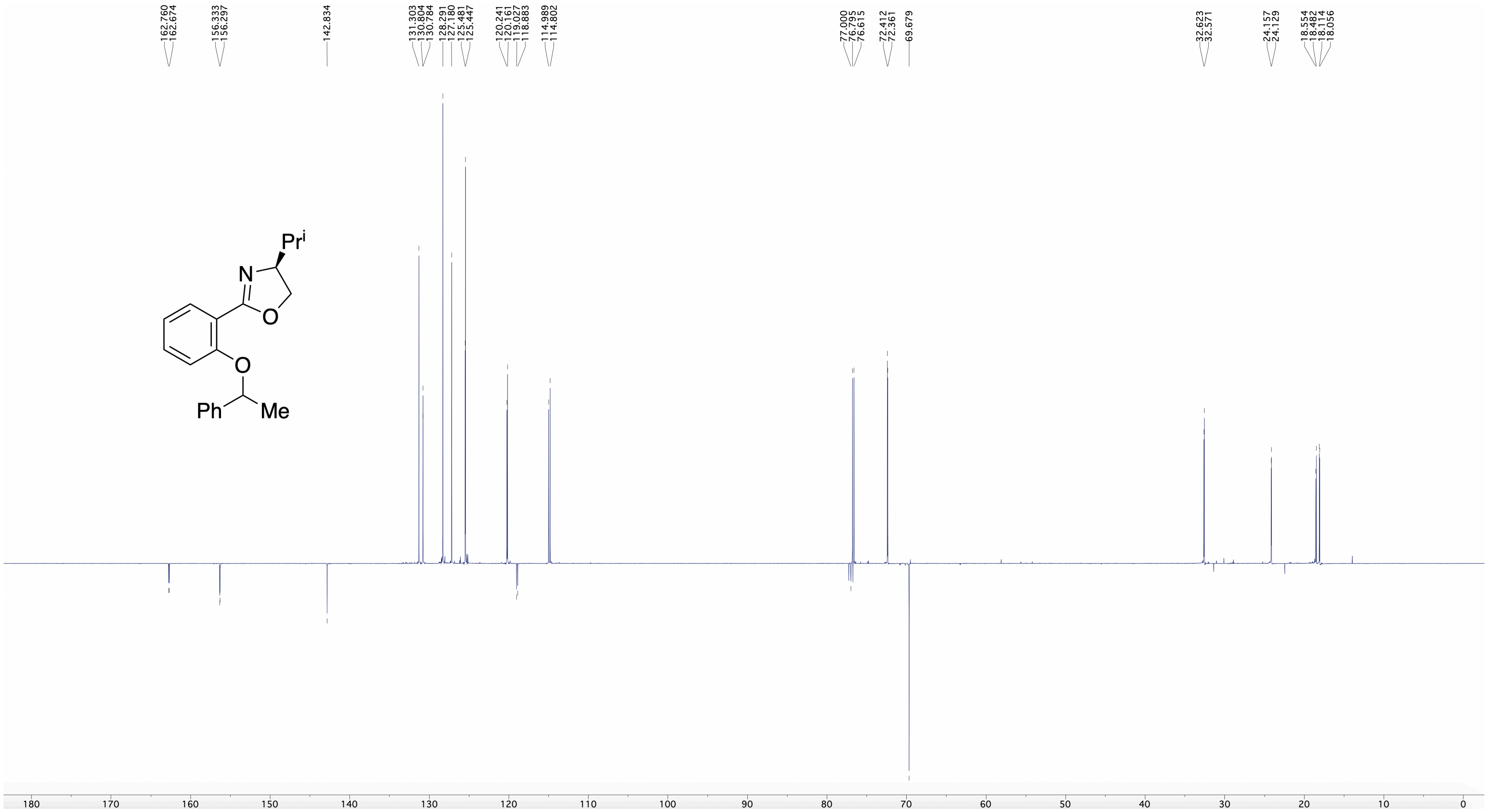

Figure S40. 100 MHz DEPTQ ^{13}C NMR spectrum of **46**

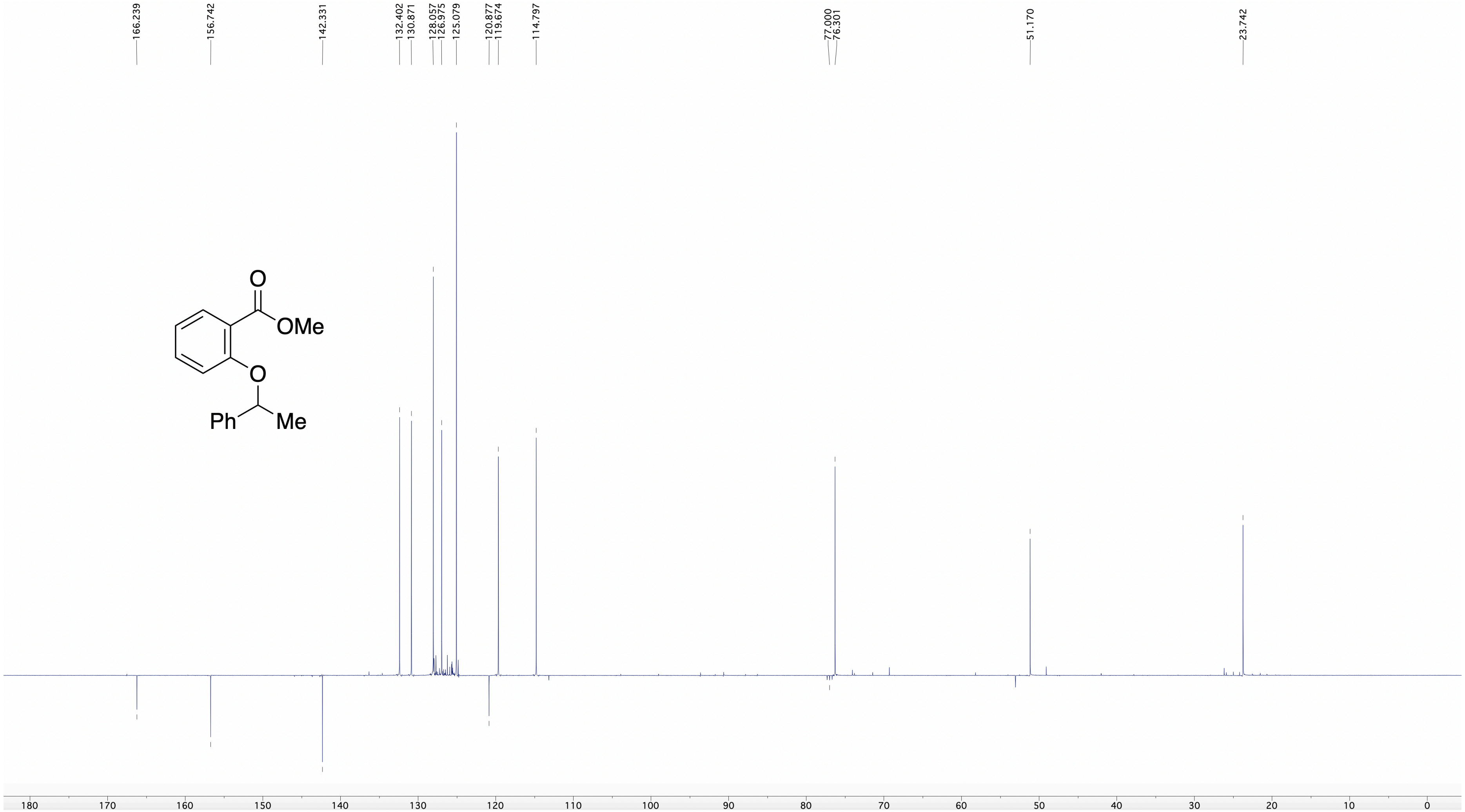

Figure S41. 400 MHz ^{1}H NMR spectrum of **47**

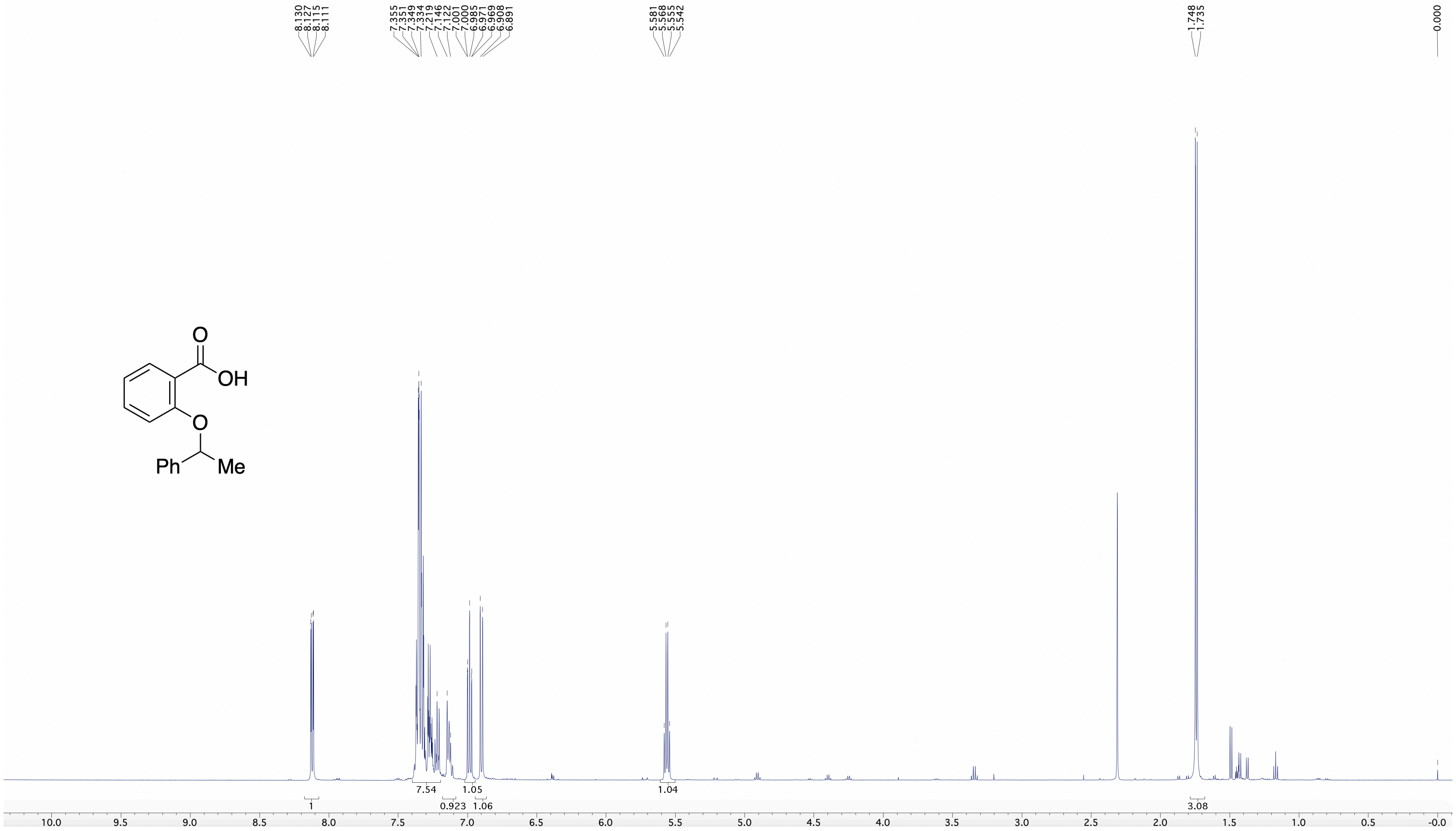

Figure S42. 100 MHz DEPTQ ^{13}C NMR spectrum of **47**

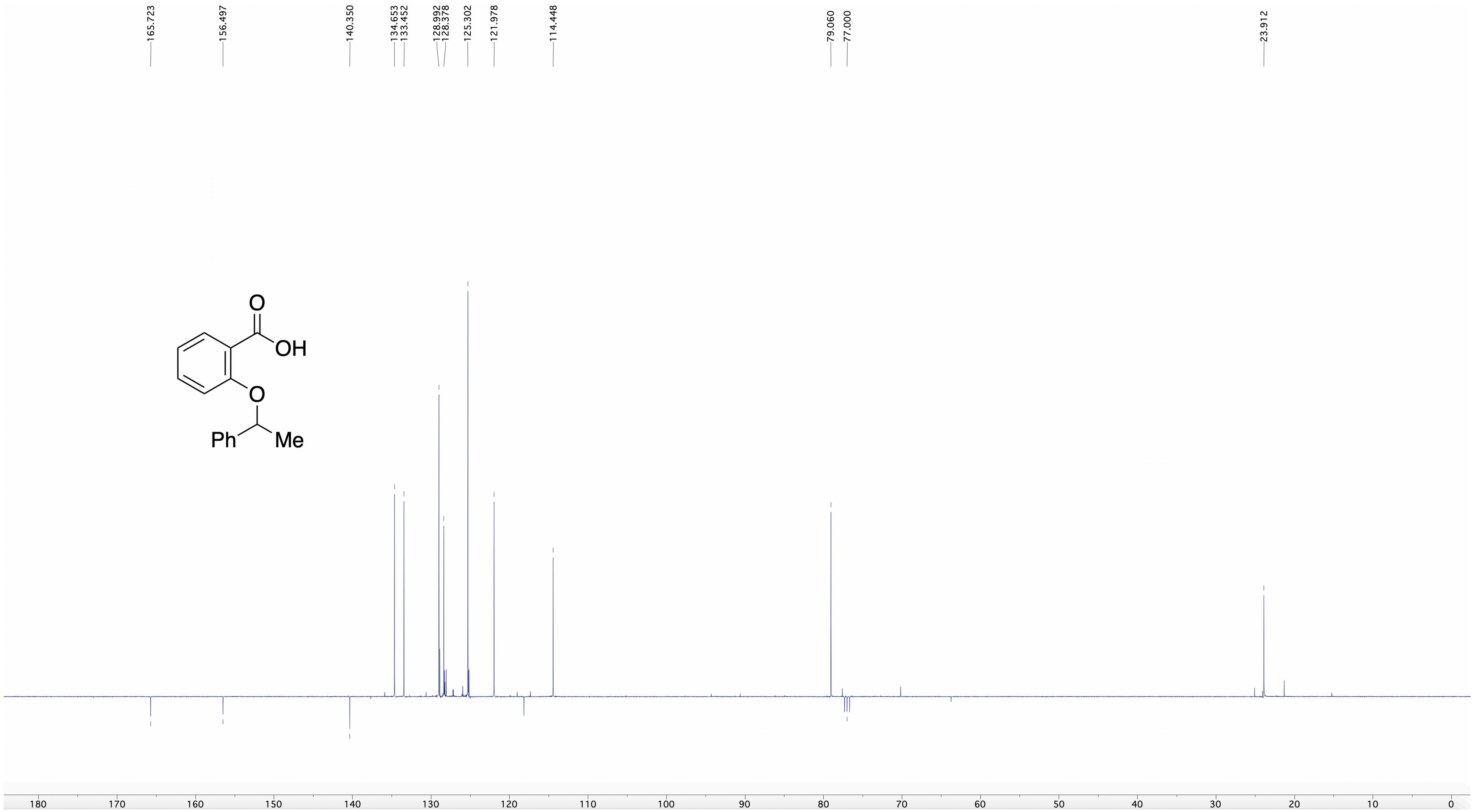

Figure S43. 400 MHz ^{1}H NMR spectrum of **48**

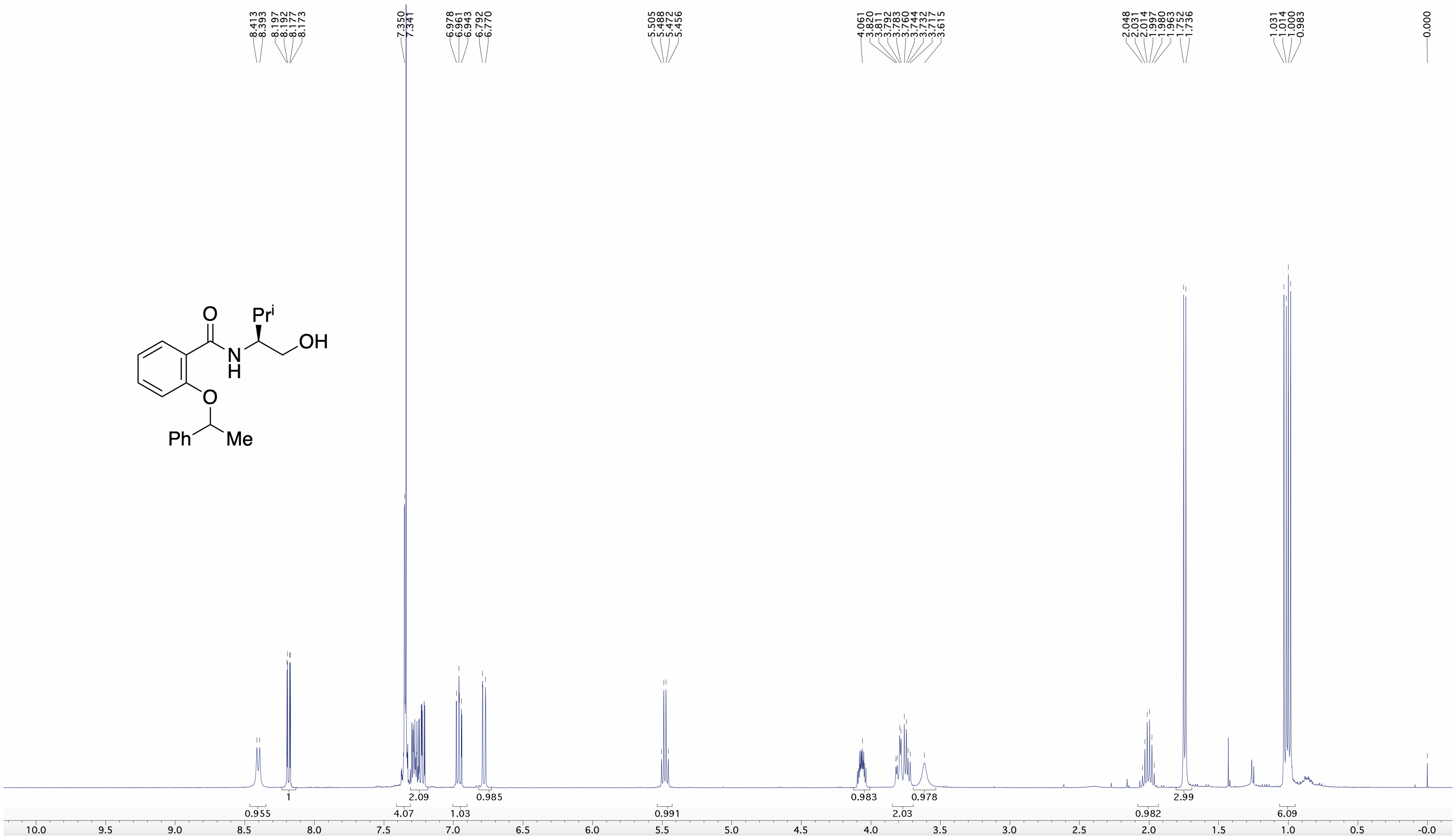

Figure S44. 125 MHz DEPTQ ^{13}C NMR spectrum of **48**

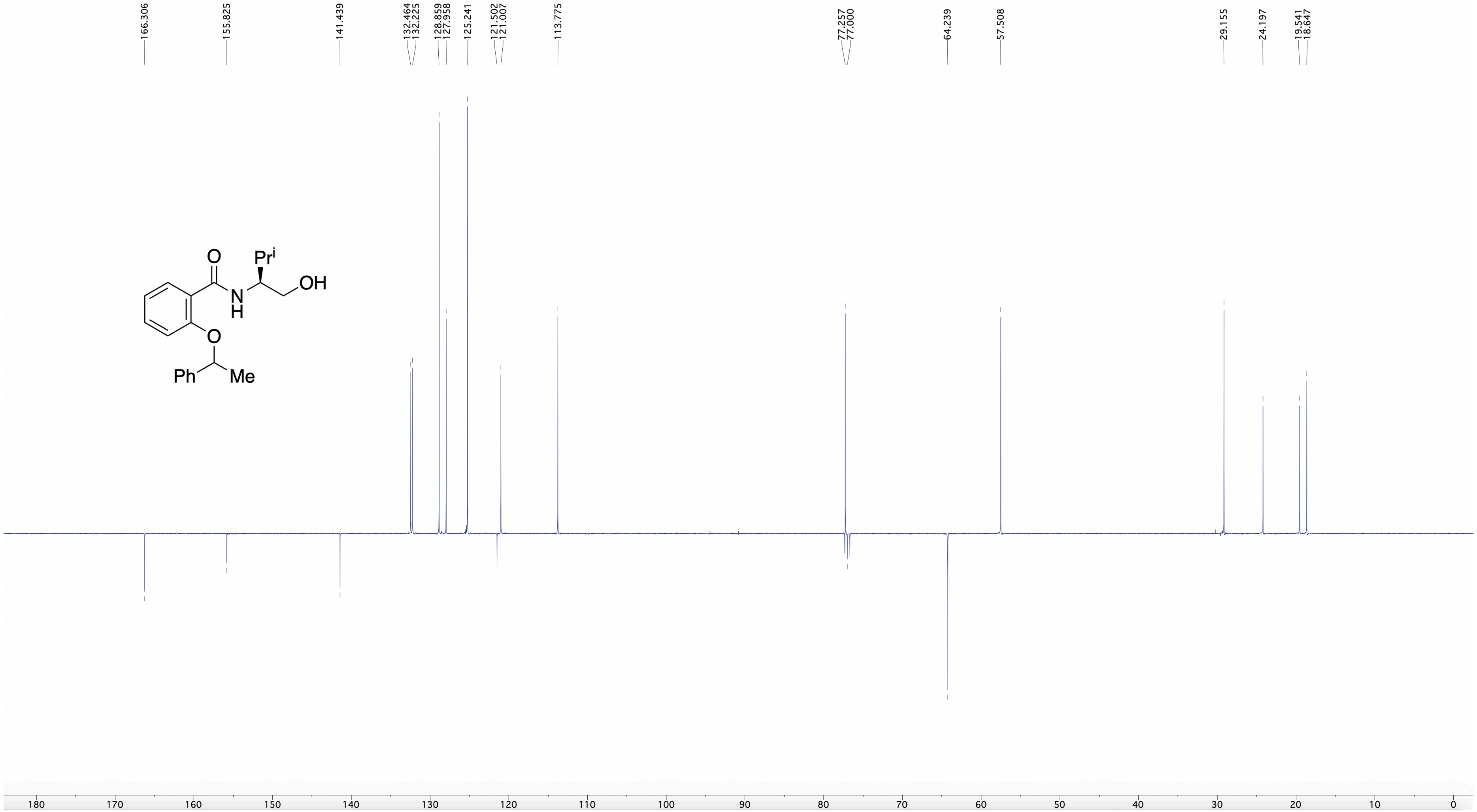

Figure S45. 100 MHz DEPTQ ^{13}C NMR spectrum of **49**

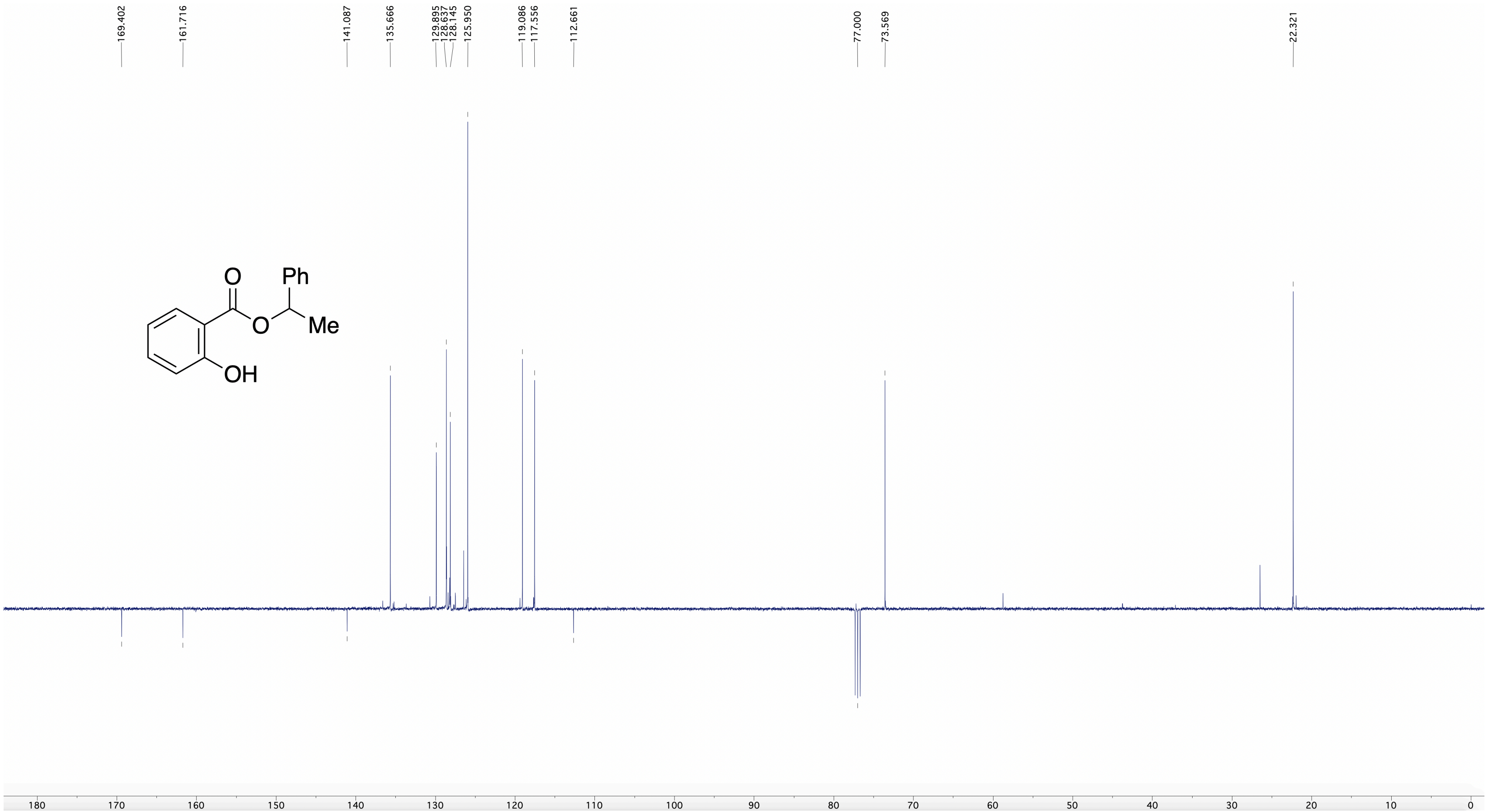

Figure S46. 400 MHz ^{1}H NMR spectrum of **50**

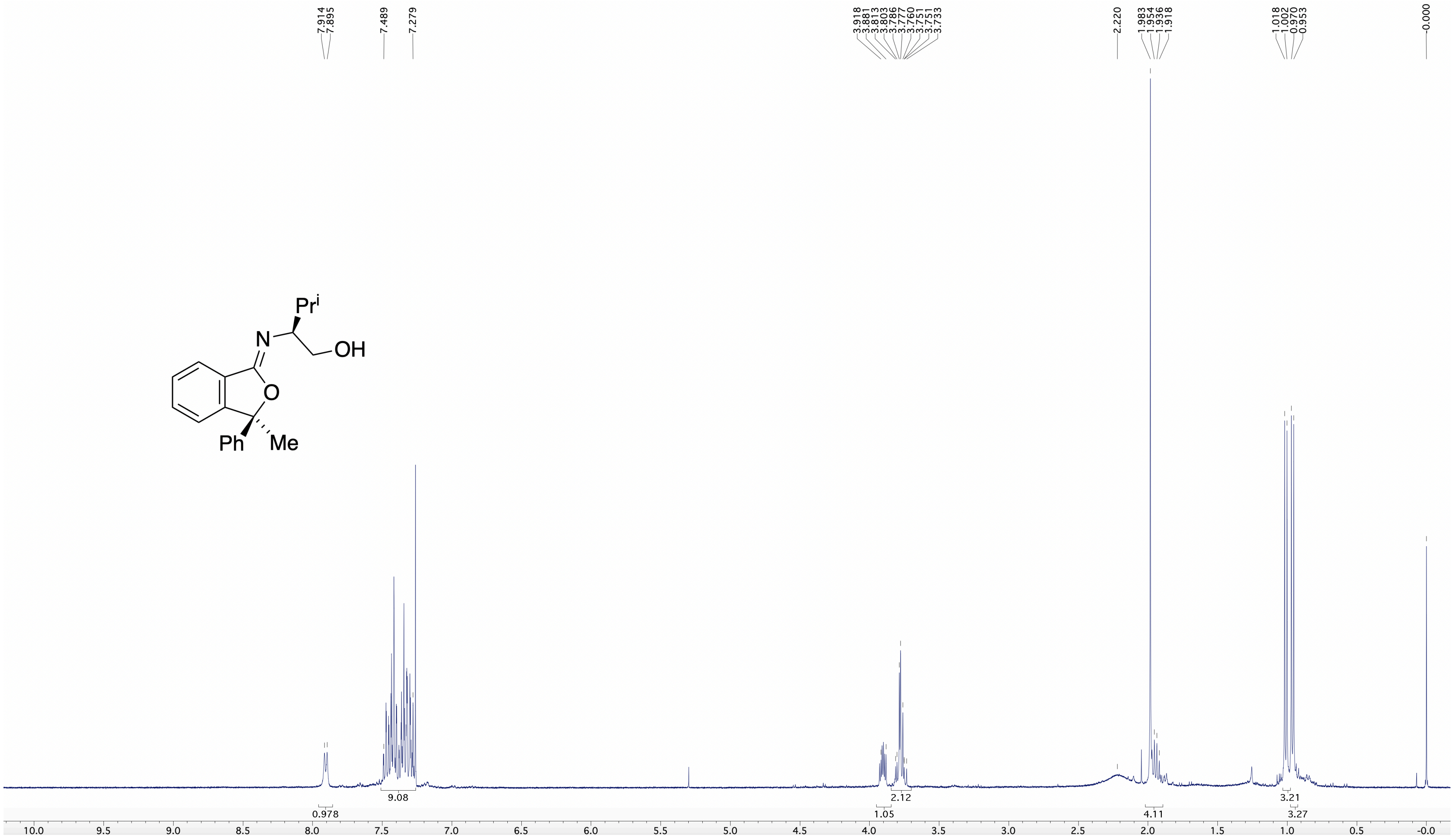

Figure S47. 125 MHz DEPTQ ^{13}C NMR spectrum of **50**

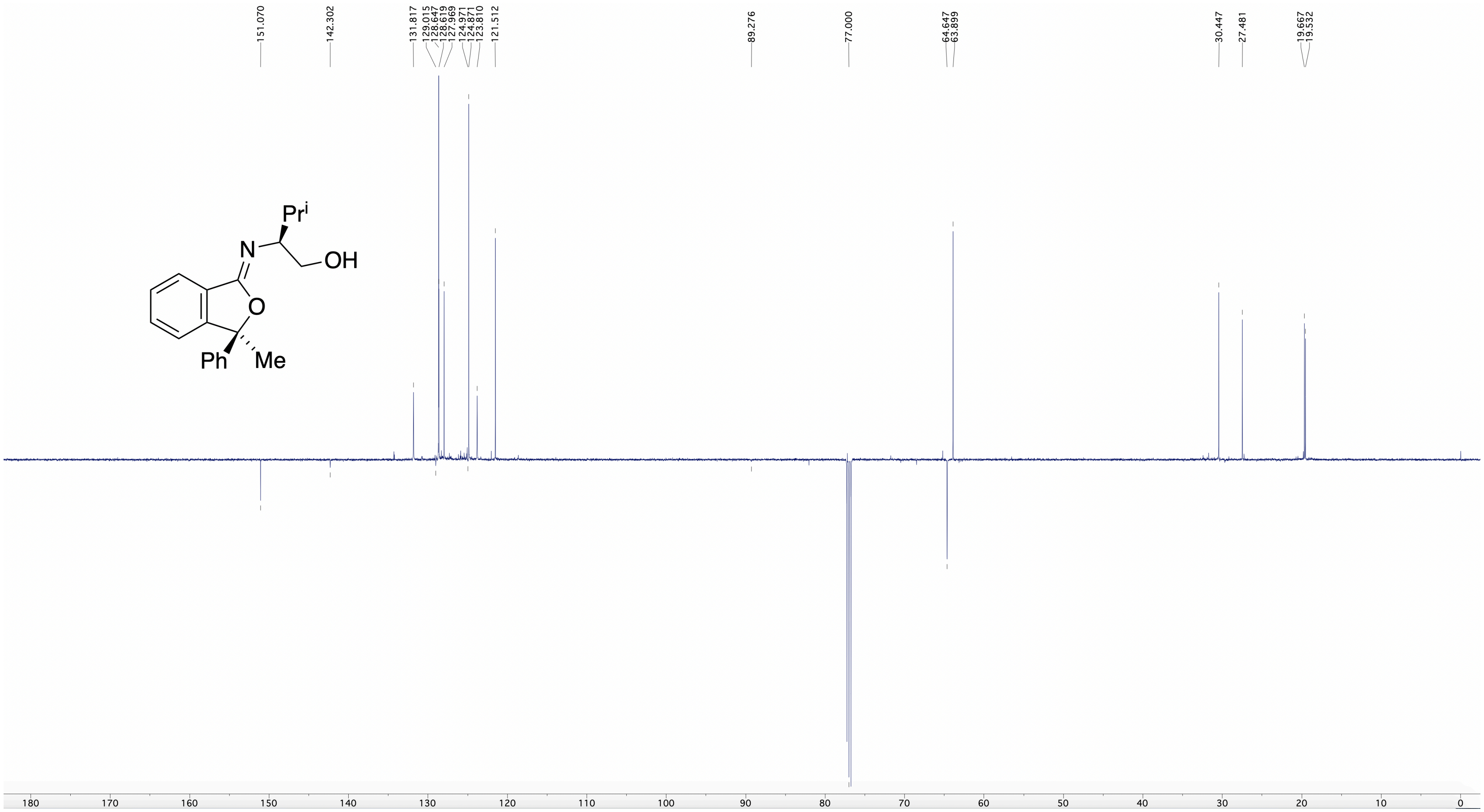

Figure S48. 300 MHz ^{1}H NMR spectrum of **54** (and **54a**)

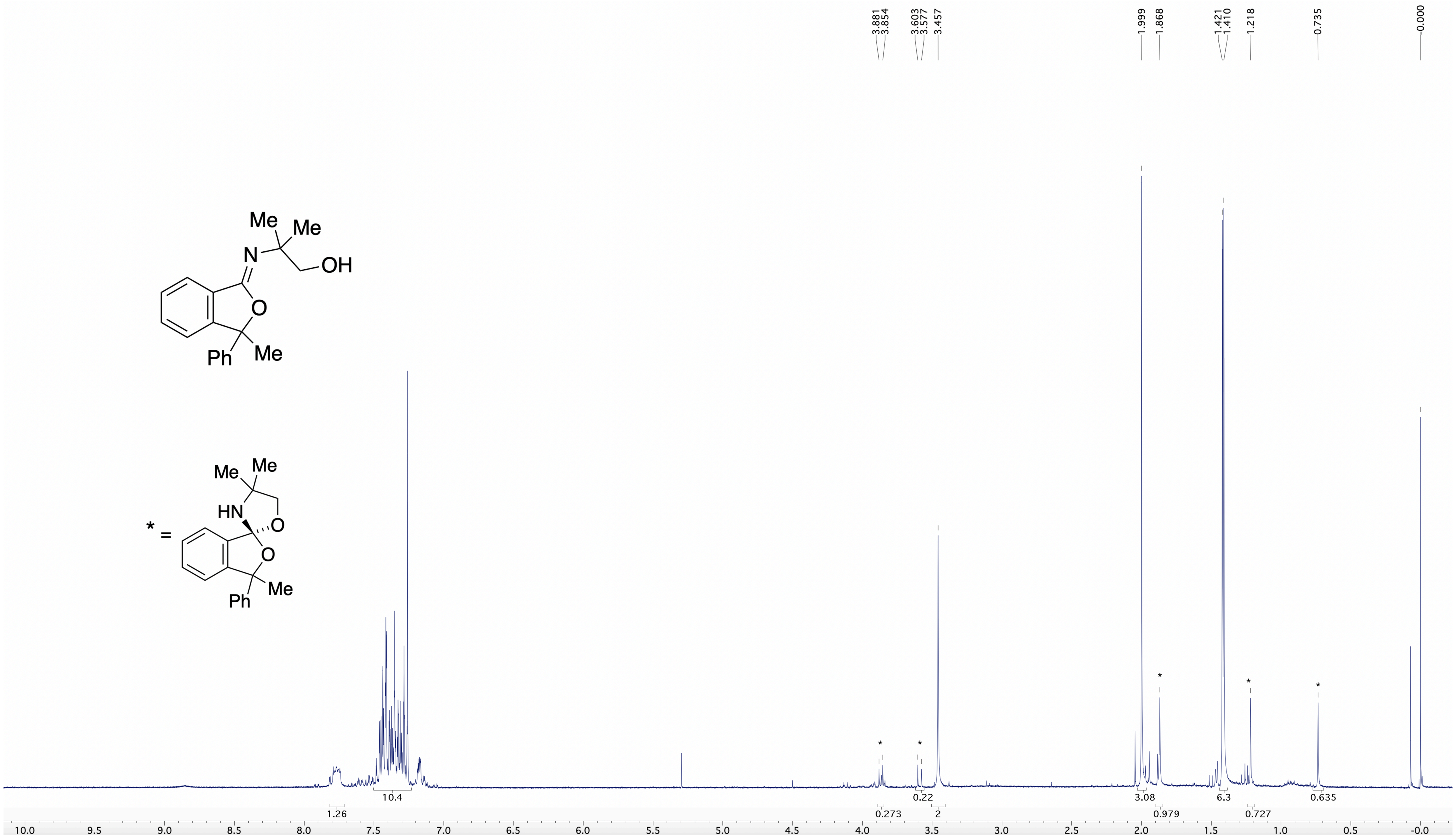

Figure S49. 125 MHz DEPTQ ^{13}C NMR spectrum of **54**

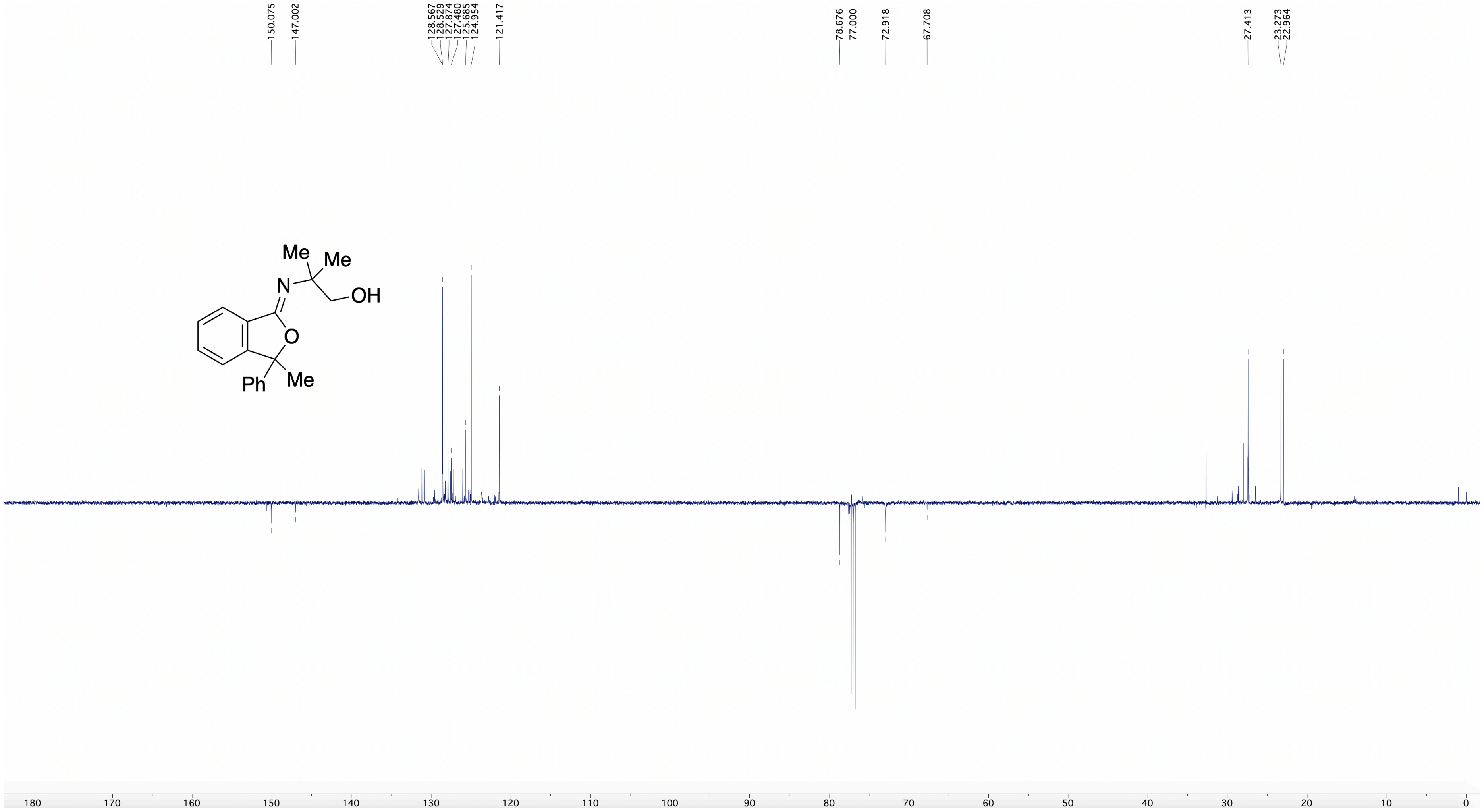

Figure S50. 400 MHz ^{1}H NMR spectrum of **55**

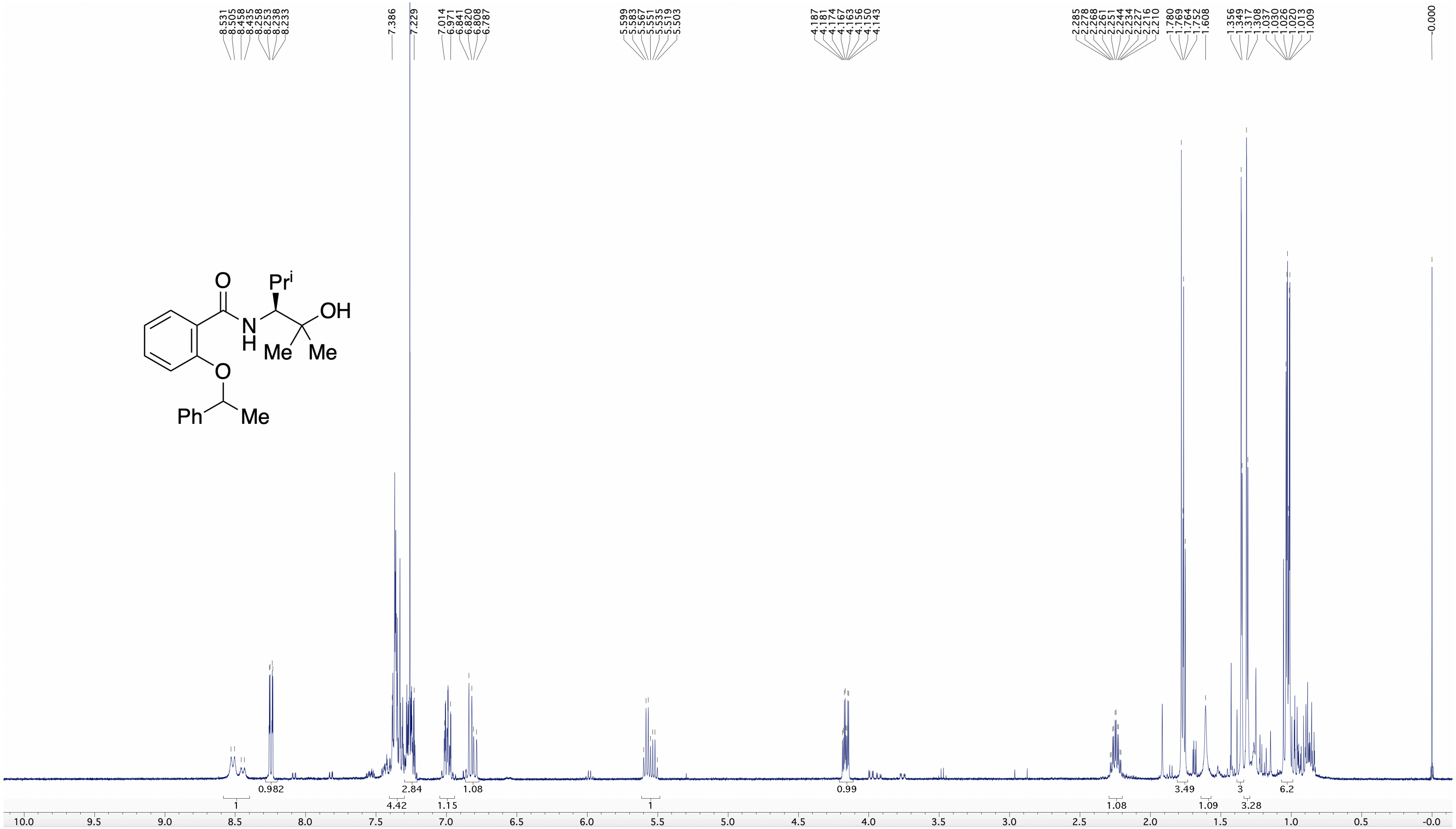

Figure S51. 125 MHz DEPTQ ^{13}C NMR spectrum of **55**

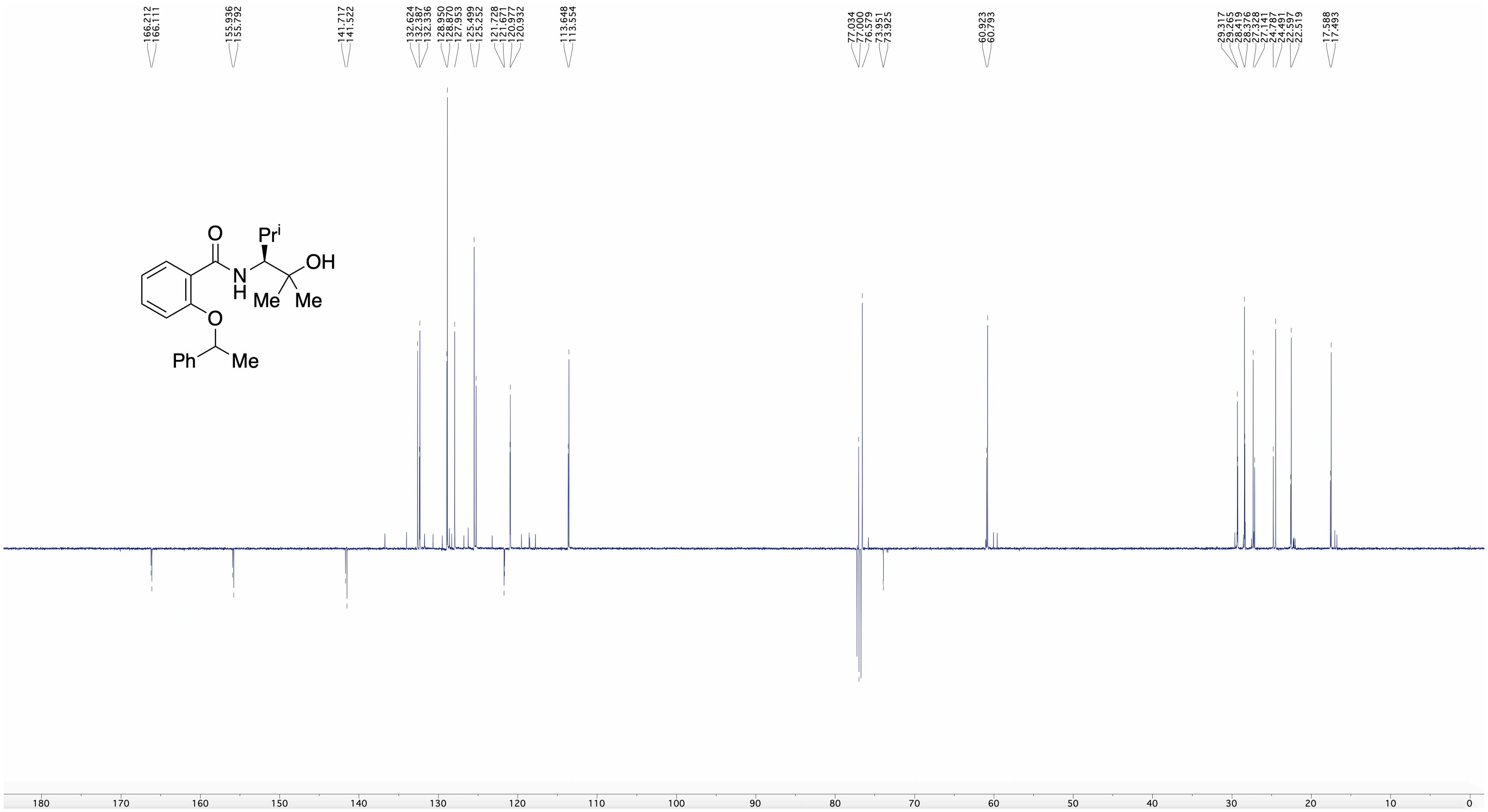

Figure S52. 400 MHz ^{1}H NMR spectrum of **56**

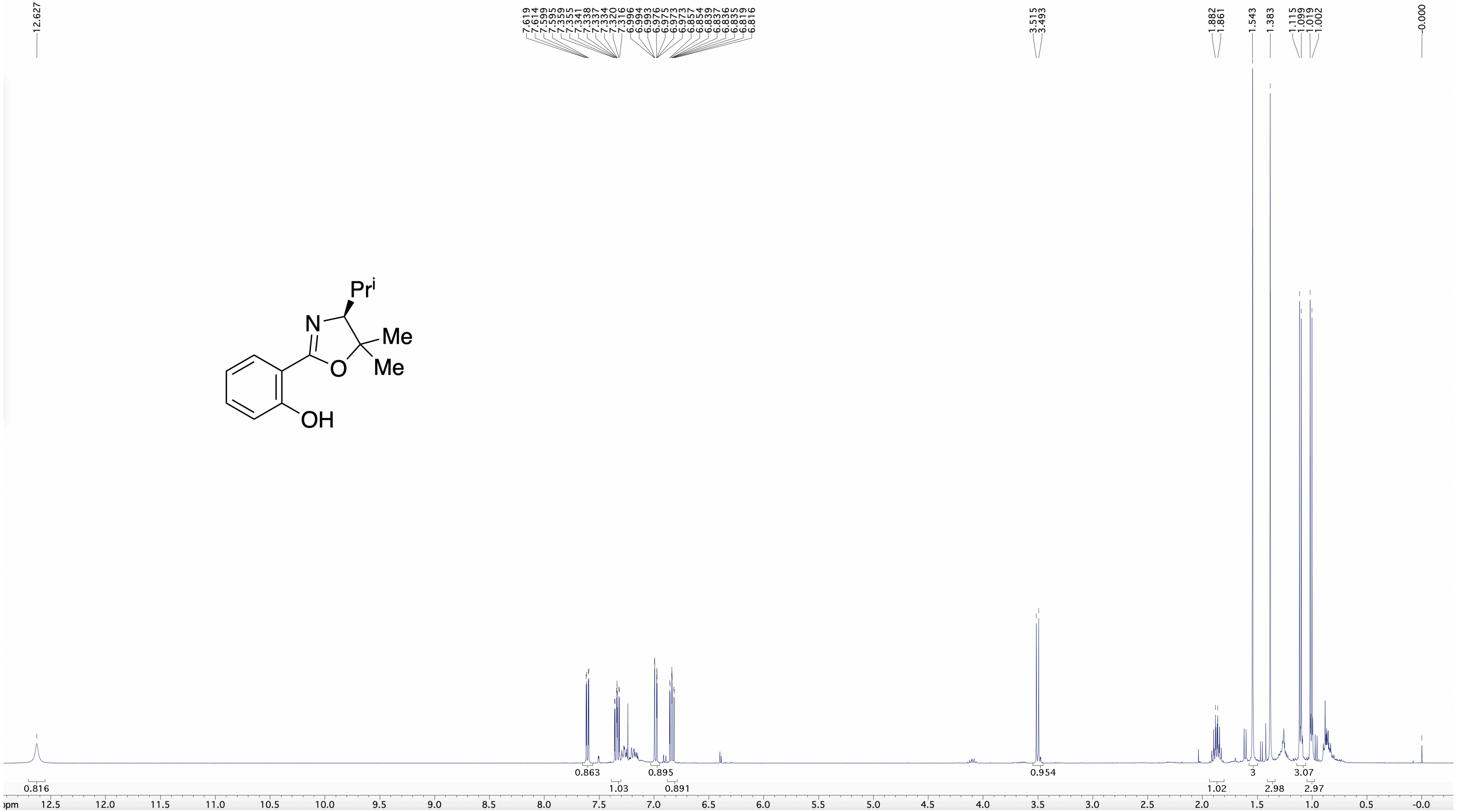

Figure S53. 100 MHz DEPTQ ^{13}C NMR spectrum of **56**

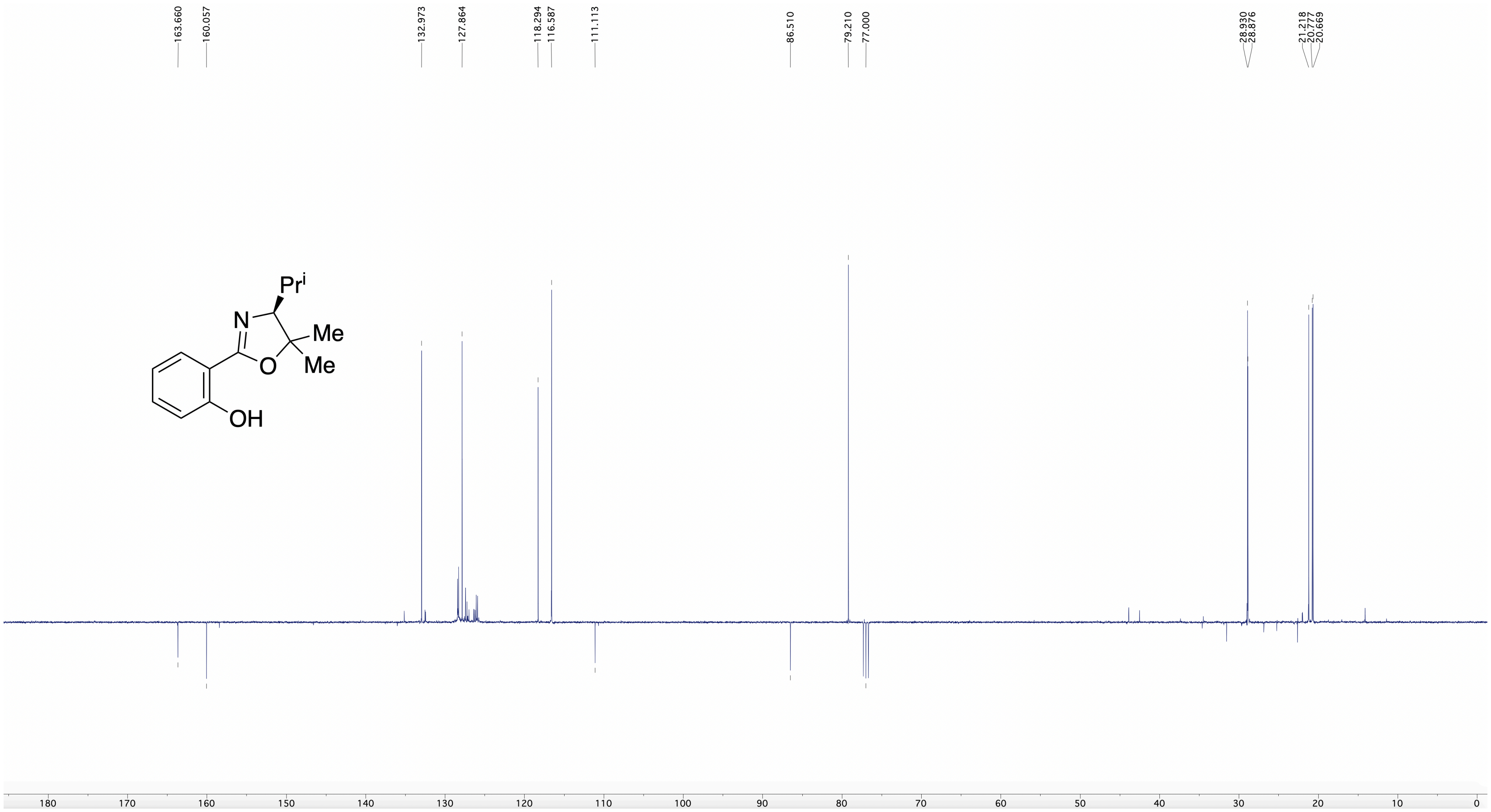

Figure S54. 400 MHz ^{1}H NMR spectrum of **57**

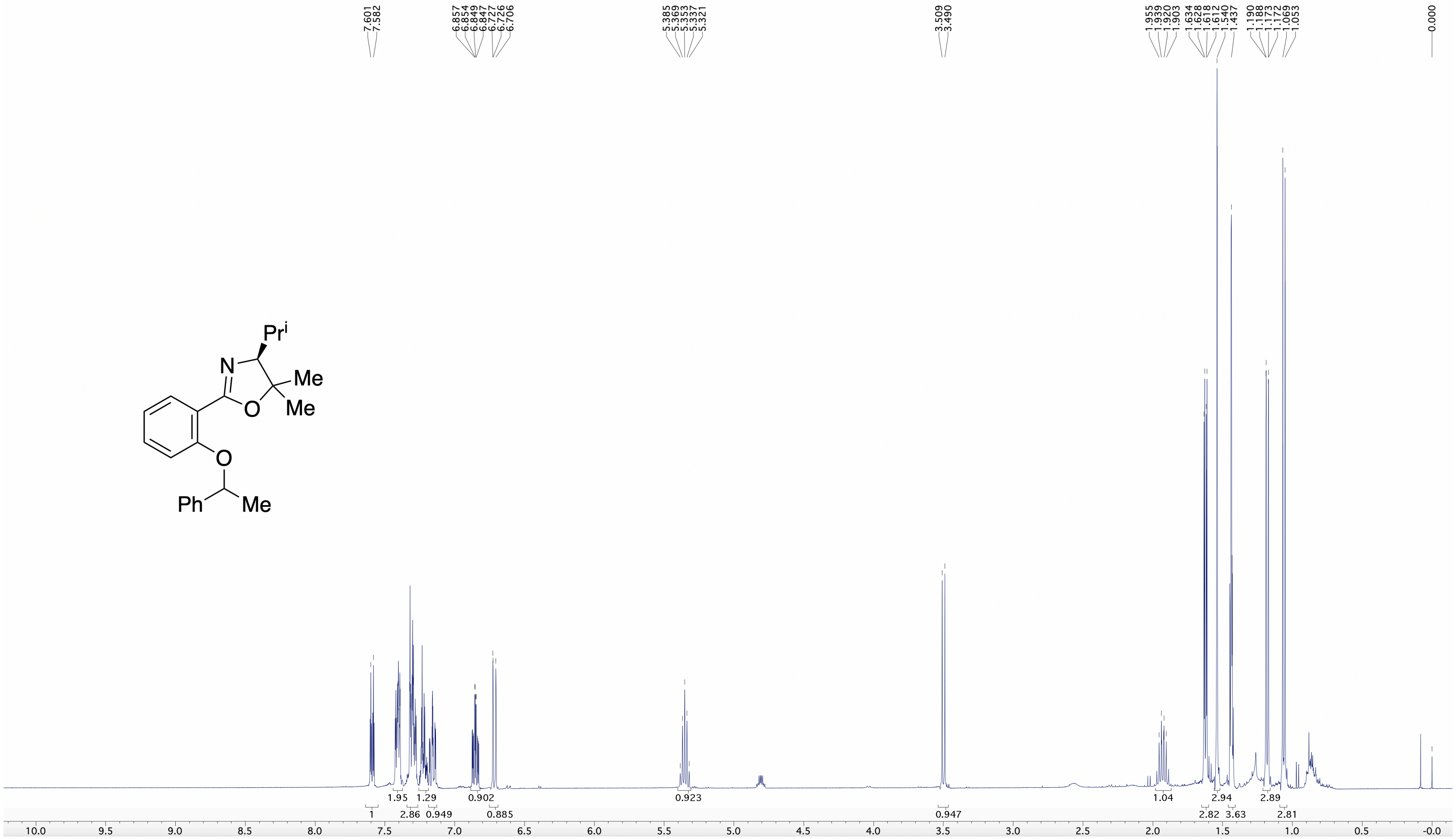

Figure S55. 100 MHz DEPTQ ^{13}C NMR spectrum of **57**

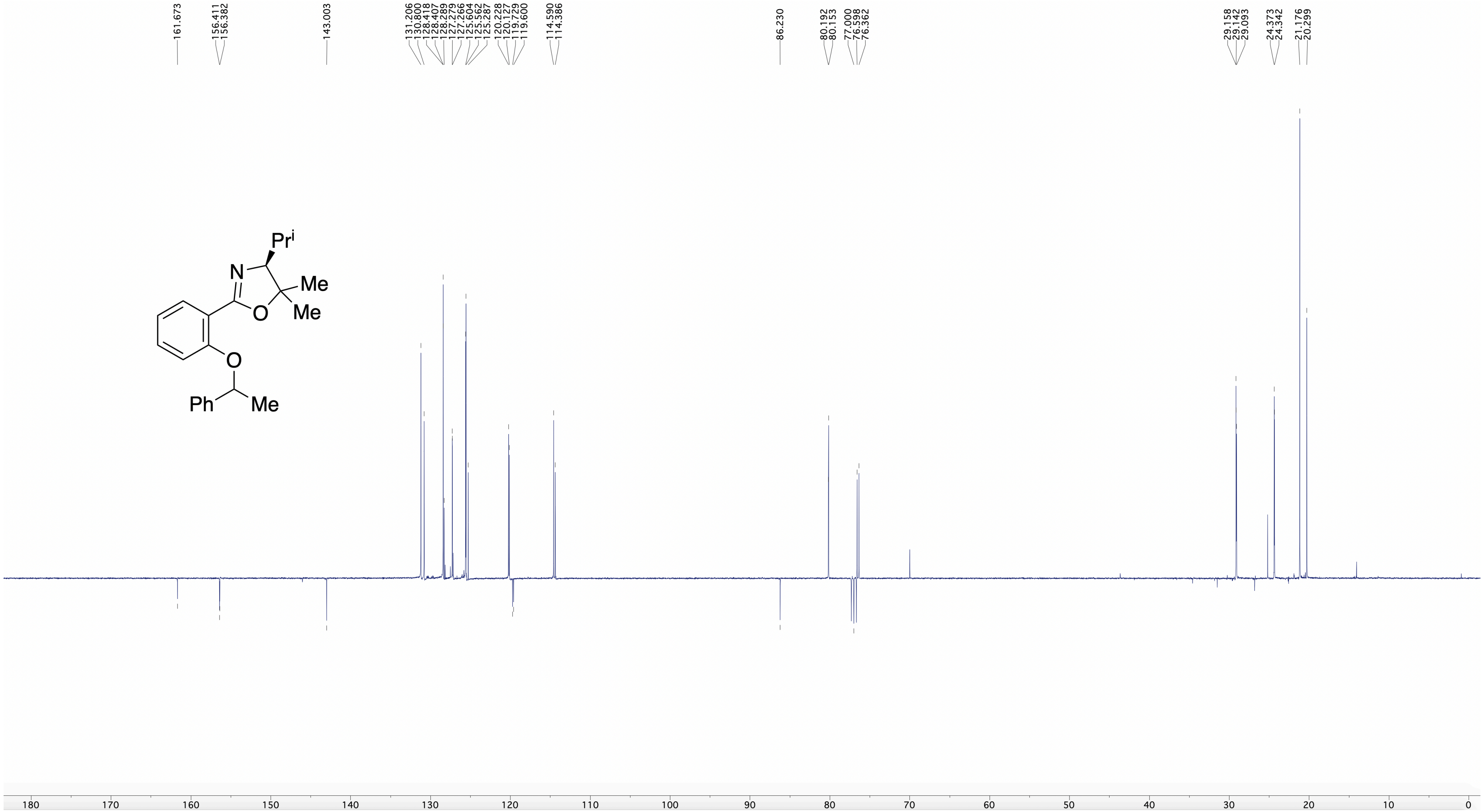

Figure S56. 400 MHz ^{1}H NMR spectrum of **58**

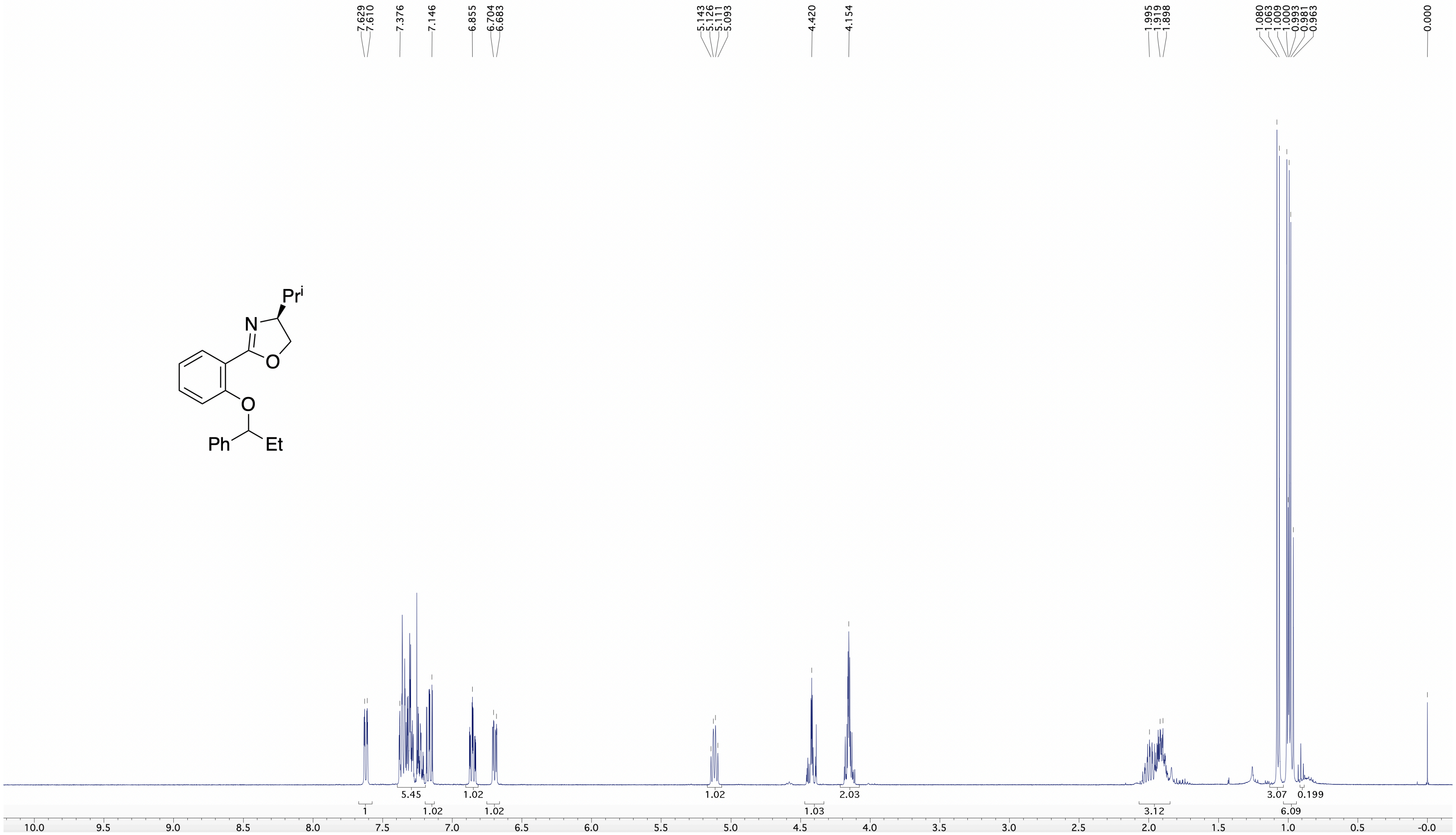

Figure S57. 100 MHz DEPTQ ^{13}C NMR spectrum of **58**

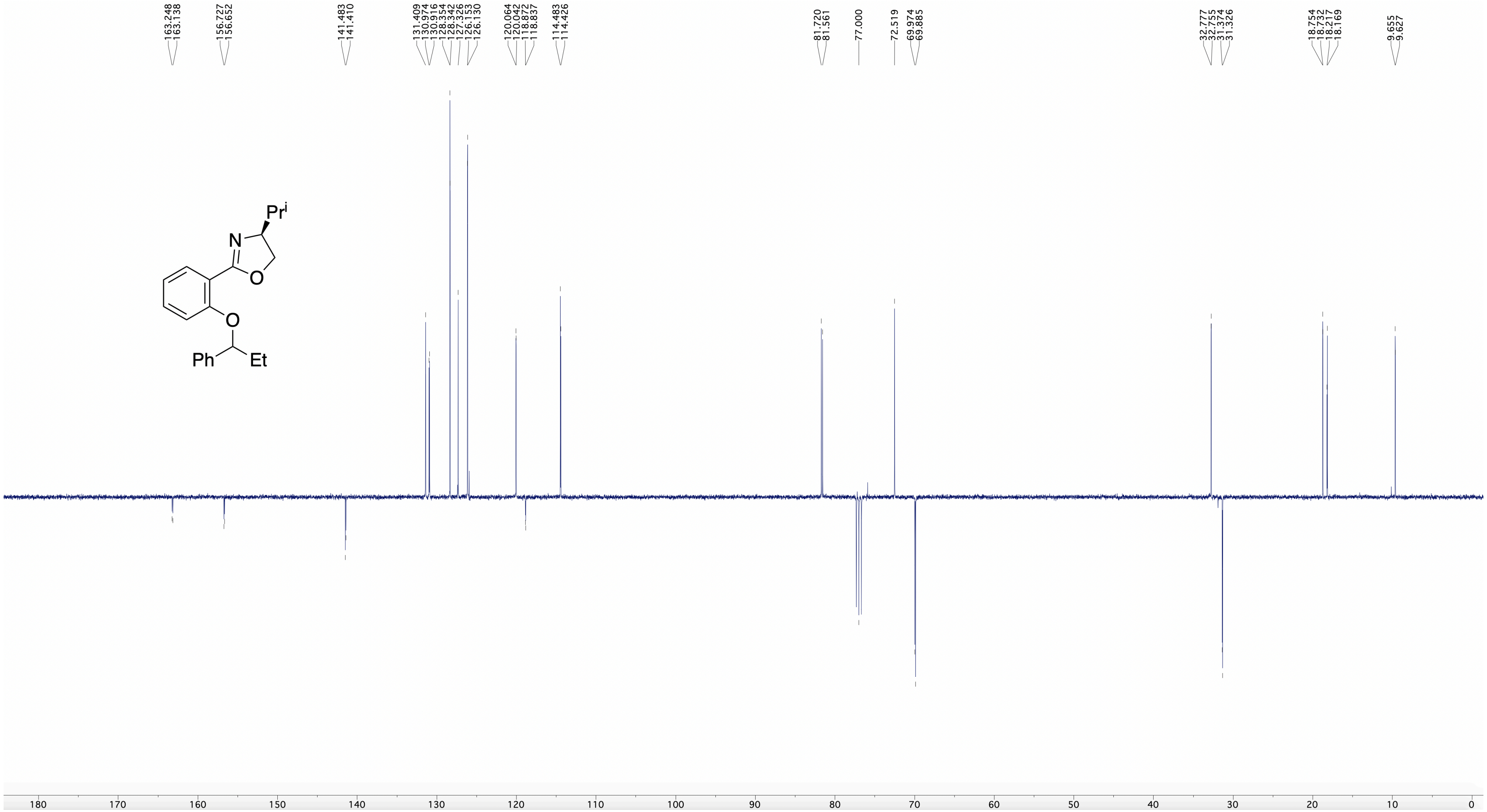

Figure S58. 400 MHz ^{1}H NMR spectrum of **59**

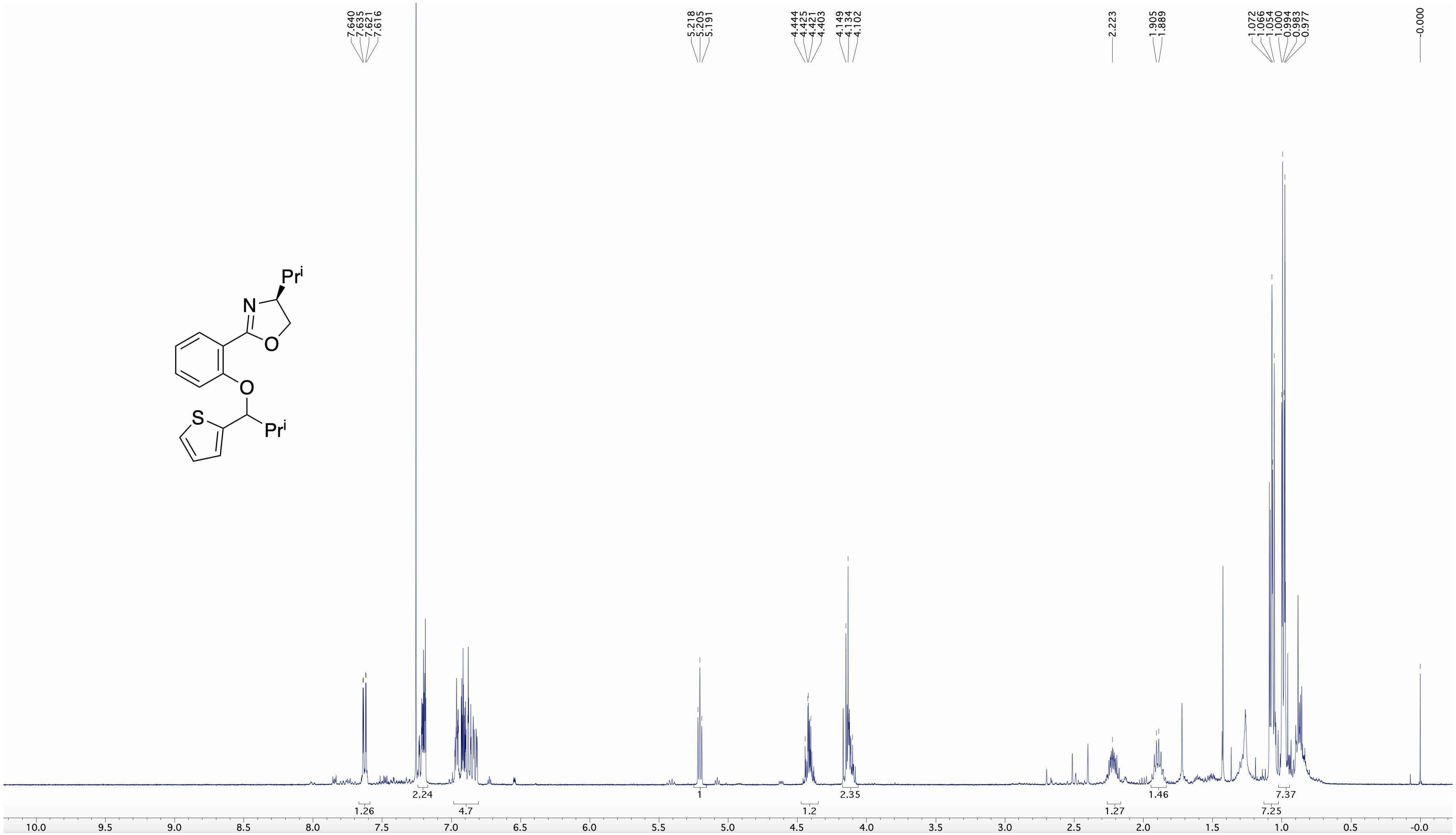

Figure S59. 100 MHz DEPTQ ^{13}C NMR spectrum of **59**

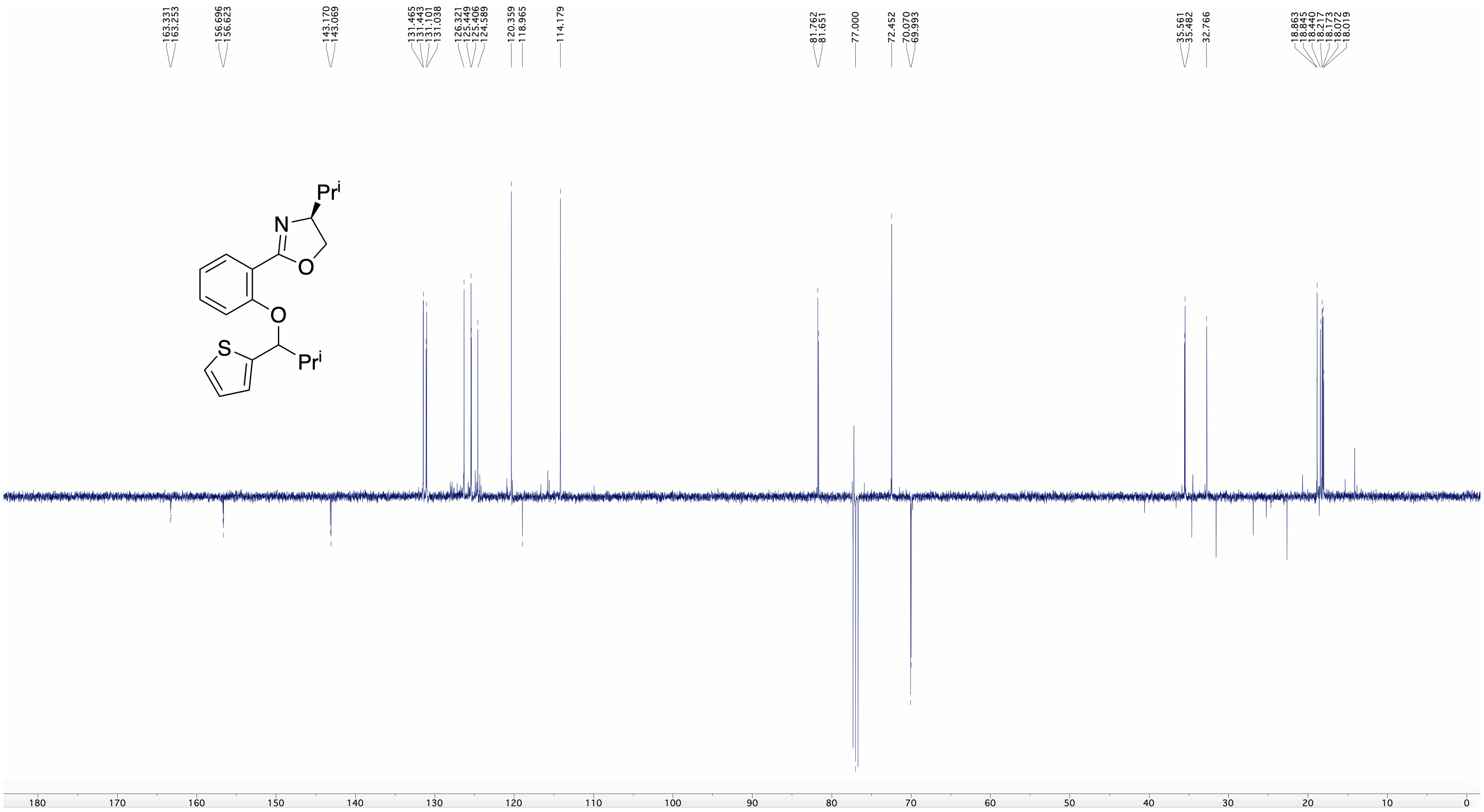

Figure S60. 400 MHz ^{1}H NMR spectrum of **60**

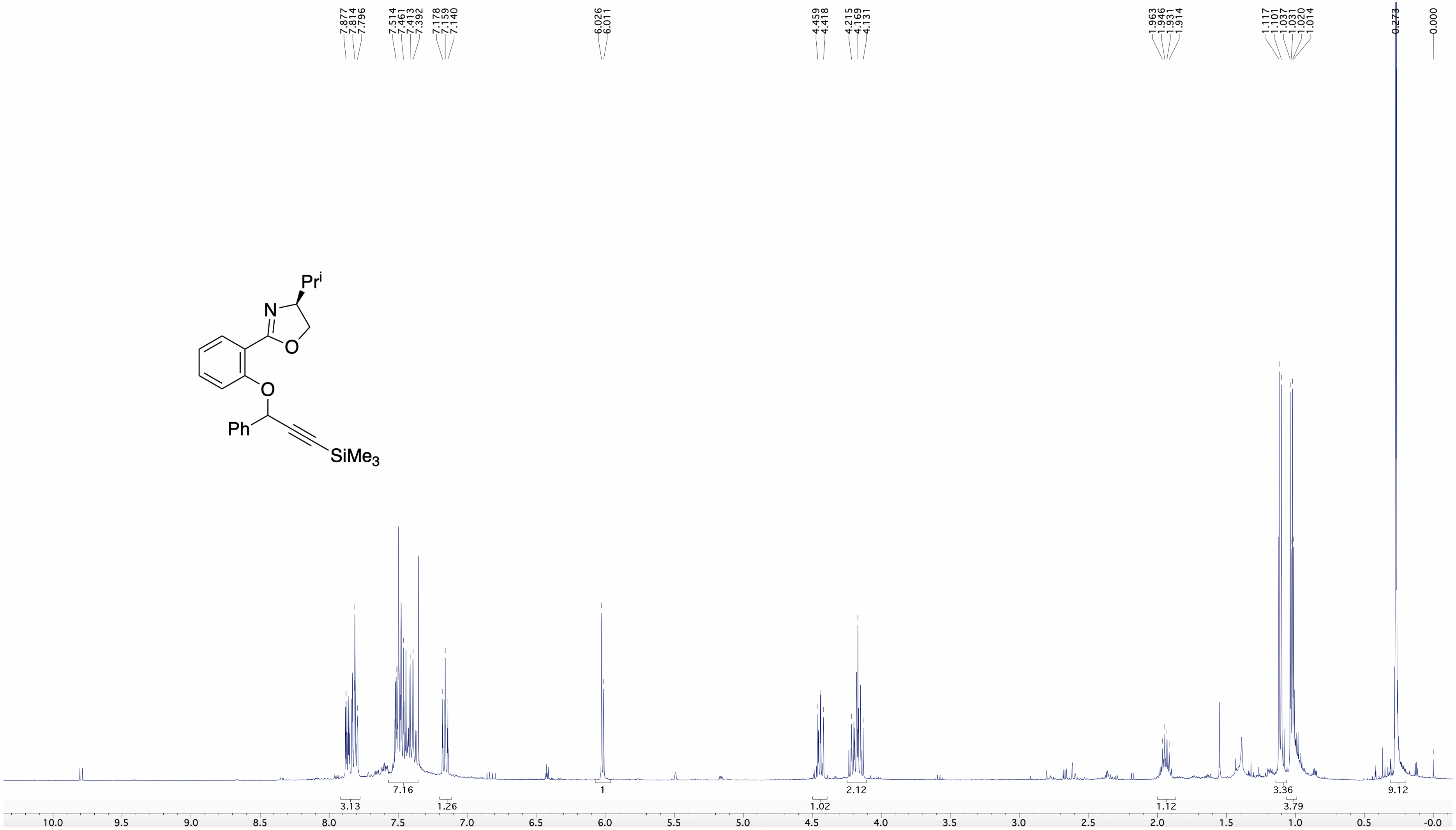

Figure S61. 100 MHz DEPTQ ^{13}C NMR spectrum of **60**

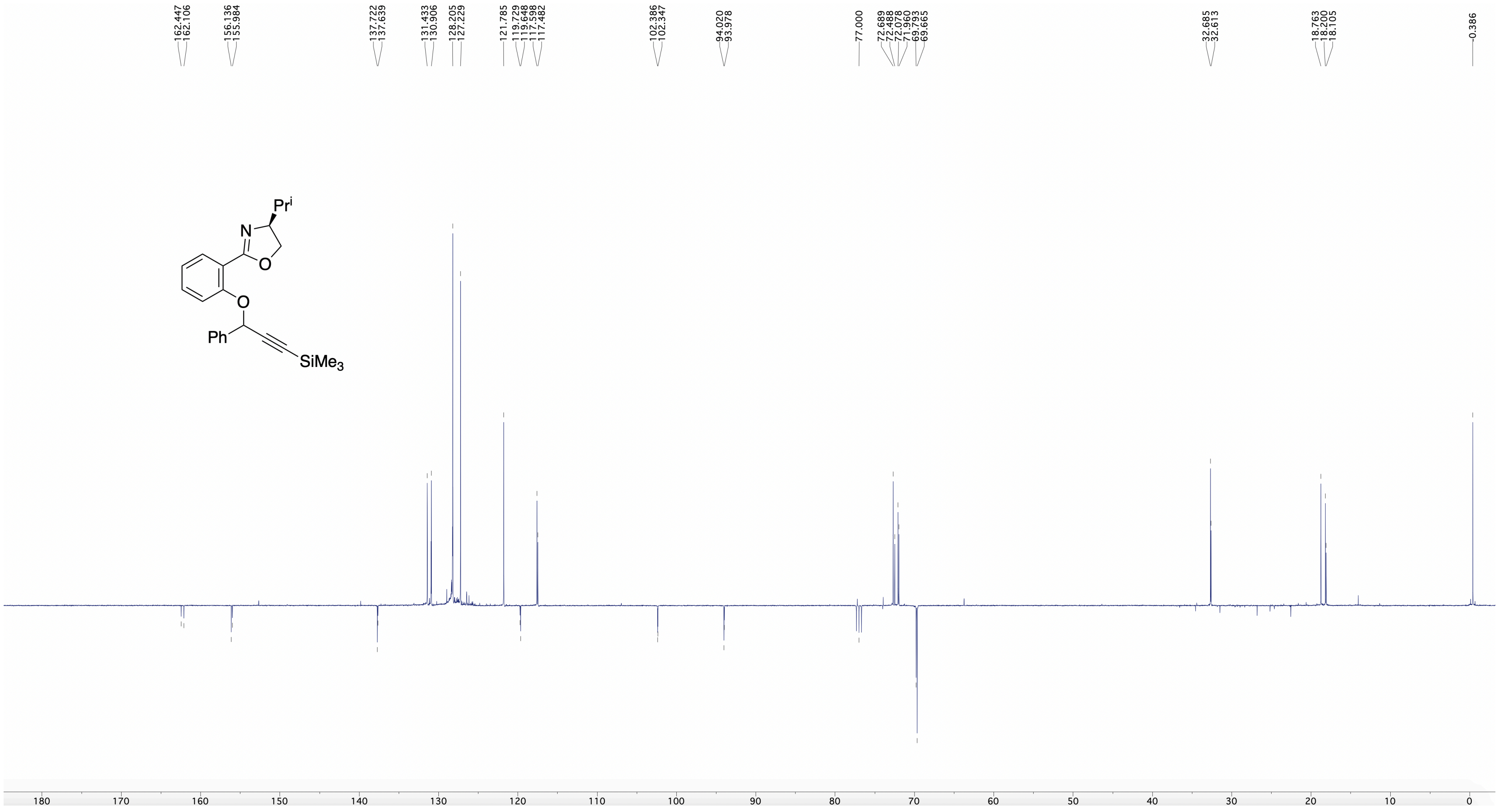

Figure S62. 400 MHz ^{1}H NMR spectrum of **61**

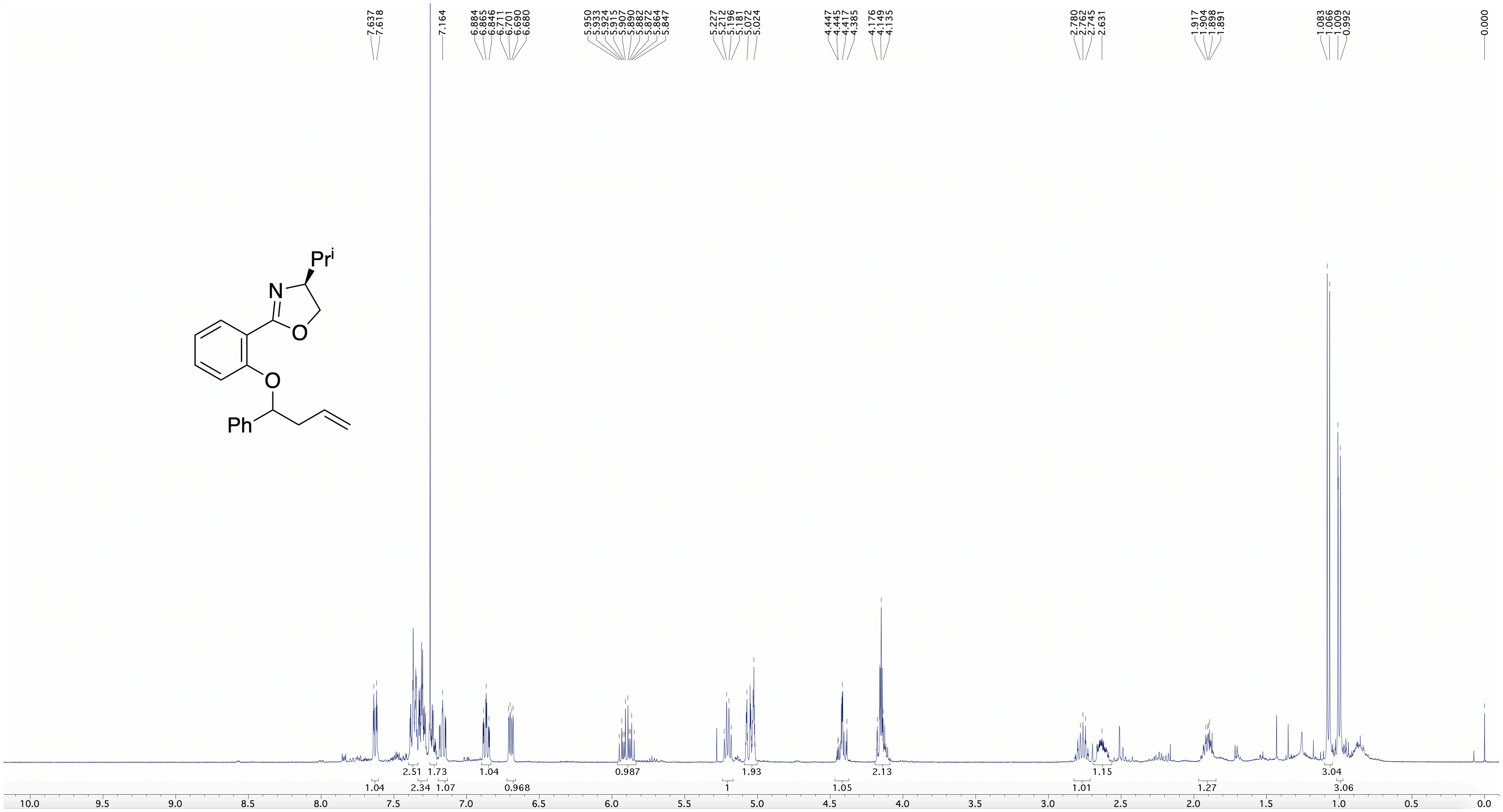

Figure S63. 100 MHz DEPTQ ^{13}C NMR spectrum of **61**

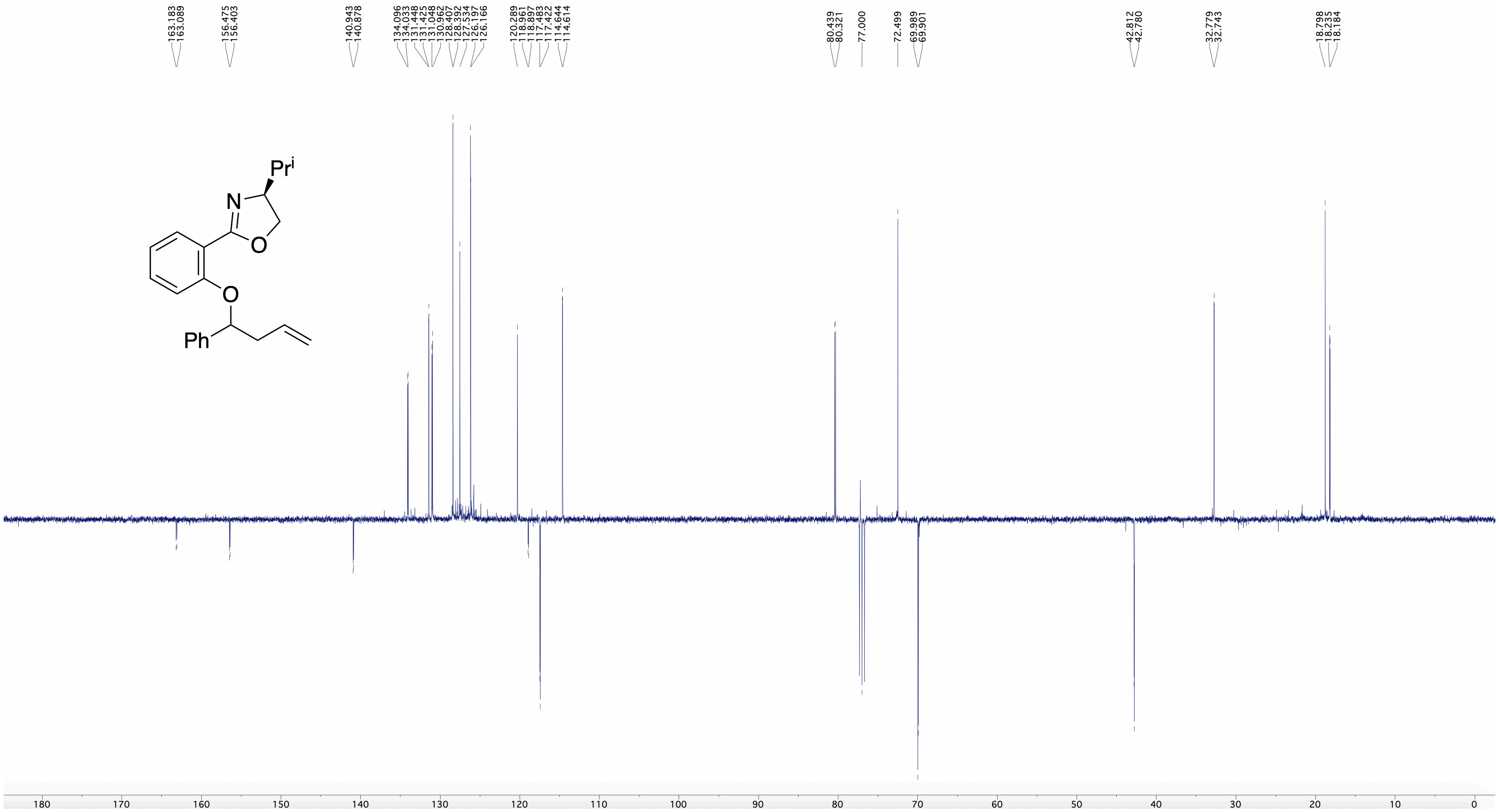

Figure S64. 400 MHz ^{1}H NMR spectrum of **63**

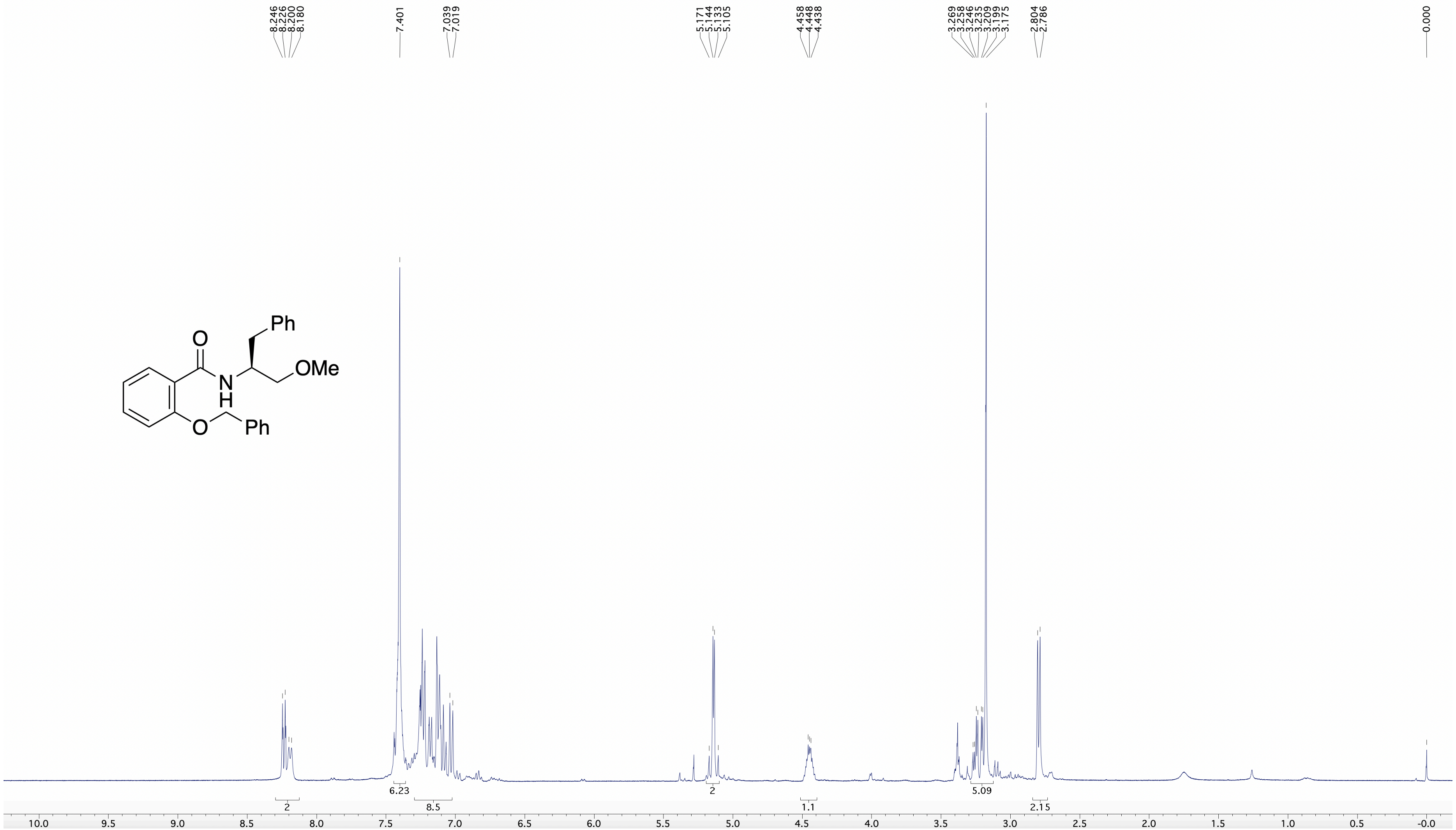

Figure S65. 100 MHz DEPTQ ^{13}C NMR spectrum of **63**

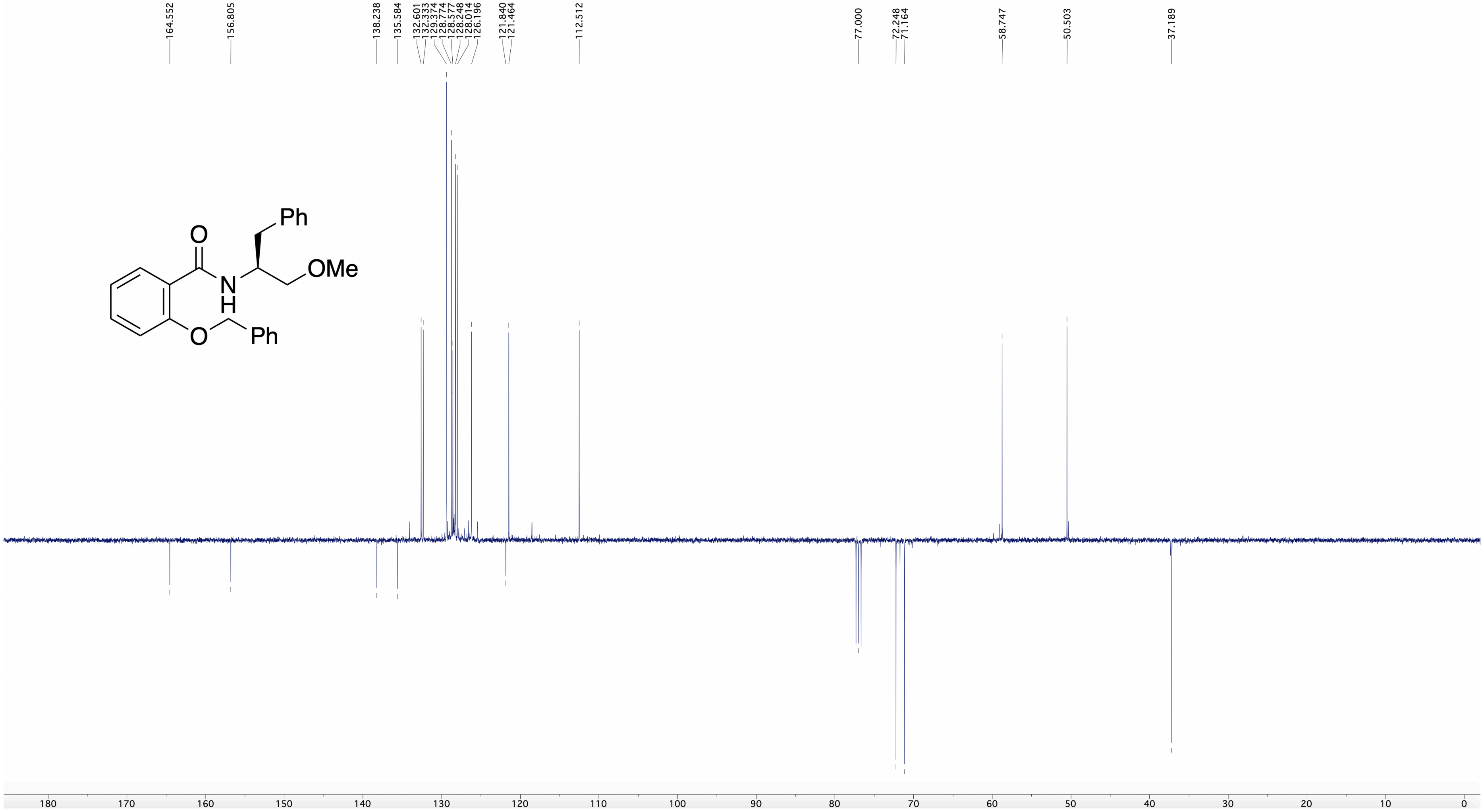

Supplement: Supplementary file 1 [file molecules-27-03186-s001.zip › molecules-1706496-supplementary.pdf]
